# Global Risk Factor Evaluation of Obstructive Sleep Apnea in Relation to Research Activity and Socioeconomic Factors

**DOI:** 10.3390/ijerph17186785

**Published:** 2020-09-17

**Authors:** Rebekka K. Seeger-Zybok, Doris Klingelhöfer, David A. Groneberg

**Affiliations:** Institute of Occupational, Social and Environmental Medicine, Goethe University of Frankfurt, 60590 Frankfurt, Germany; klingelhoefer@med.uni-frankfurt.de (D.K.); groneberg@med.uni-frankfurt.de (D.A.G.)

**Keywords:** OSA, public health, epidemiology, risk factor, bibliometrics, prevalence, obesity

## Abstract

Obstructive Sleep Apnea is emerging as a global health epidemic, particularly due to the obesity pandemic. However, comprehensive prevalence data are still lacking and global OSA research has not yet been structurally evaluated. Using the latest comprehensive age/gender-specific BMI and obesity data, a global landscape estimating the risk/burden of OSA was created. Results were presented in relation to an in-depth analysis of OSA research and countries’ socioeconomic/scientific background. While the USA, Canada, and Japan are the highest publishing countries on OSA, Iceland, Greece, and Israel appeared at the forefront when relating the scientific output to socioeconomic parameters. Conversely, China, India, and Russia showed relatively low performances in these relations. Analysis of the estimated population at risk (EPR) of OSA showed the USA, China, India, and Brazil as the leading countries. Although the EPR and OSA research correlated strongly, major regional discrepancies between the estimated demand and actual research performances were identified, mainly in, but not limited to, developing nations. Our study highlights regional challenges/imbalances in the global activity on OSA and allows targeted measures to mitigate the burden of undiagnosed/untreated OSA. Furthermore, the inclusion of disadvantaged countries in international collaborations could stimulate local research efforts and provide valuable insights into the regional epidemiology of OSA.

## 1. Introduction

Obstructive sleep apnea (OSA) is the most common entity among sleep-related breathing disorders [1]. It is characterized by daytime sleepiness, fatigue, poor concentration, and depression [2,3,4]. Repetitive periods of pharyngeal collapse during sleep lead to intermittent disruptions of gas exchange and result in hypoxia and hypercapnia, causing reactive respiratory effort-related arousals, fragmented sleep, nocturnal sympathetic nervous system activation, elevated markers of oxidative stress, and inflammation [5,6]. This contributes to an increased risk of, e.g., cardiovascular, metabolic, and neurocognitive morbidities [2,7,8], and in the case of severe untreated OSA, to an elevated all-cause mortality independent of other risk factors [9]. Obesity, male gender, age, and craniofacial/upper airway abnormalities are the major risk factors for OSA [10,11]. Currently, OSA is emerging as a global health epidemic with an increasing prevalence mainly due to the ongoing obesity epidemic [12]. Based on the impact of OSA on morbidity and mortality, as well as its societal and workplace consequences, OSA constitutes a rising economic burden worldwide [10,13]. According to the American Academy of Sleep Medicine (Darien, Illinois, USA) in 2015, the annual costs of undiagnosed OSA were estimated to be 150 billion $ per year solely in the USA [14]. However, despite increasing recognition of OSA as a complex disorder, and the technical advancements in the recent years, the gold standards of diagnosis and effective treatment are still linked to complex and expensive technology, and continuous positive airway pressure therapy (CPAP) as a first-line treatment is still characterized by poor adherence [15,16]. Moreover, comprehensive prevalence data of OSA are still lacking for most countries [17], and despite a growing body of evidence of the high variability in the prevalence, diagnosis/treatment, and severity of OSA depending on age, sex, and race/ethnicity, the present management strategies generally consist of a quite rigid one-size-fits-all approach [18].

Given the socioeconomic strain of undiagnosed/untreated OSA [19], there is a tremendous need to find cost-effective treatment alternatives and to further enhance novel and personalized approaches reflecting the true burden and satisfying the individual needs of the heterogeneous world population. Thus, to uncover the countries and areas most affected by a mismatch between research activity and estimated burden, we here present an in-depth analysis of the global OSA research in relation to a global risk factor analysis of OSA and socioeconomic characters of the publishing countries. Using the NewQIS platform, created by the Institute of Occupational, Social and Environmental Medicine in Frankfurt/Germany (New Quality and Quantity Indices in Science), our study is the first to depict the global OSA research in terms of its quantitative and qualitative aspects, its international networks, and geographical and chronological developments [20]. In the absence of comprehensive global prevalence data, we determined the estimated country-specific risk- and burden of OSA based on risk factor evaluation and epidemiological data.

## 2. Materials and Methods

### 2.1. NewQIS Platform and Search Strategy

This study used the NewQIS platform methodologies to present the global OSA research architecture between 1900 to 2018 [21]. Founded in 2009, the platform aims to inform scientists, physicians, health-care policy makers, and funders with in-depth information on various research areas related to life sciences. It combines the use of visualization techniques such as density equalizing map projections (DEMPs) [22] and established scientometric and socioeconomic analysis tools.

For the data acquisition, we employed the Core Collection of the online database Web of Science (WoS; currently Clarivate Analytics). It offers the unique feature of comprehensive citation analysis by the means of the Journal Citation Report in combination with concurrent retrieval of supporting bibliographic data.

To get the best estimate of OSA-related publications, we used the entry-terms of the Medical Subjects Headings (MeSH) of the PubMed database to create a unique search term. The overall OSA research activity in the period from 1900 to 2013 and from 2014 to 2018 was determined by a “Topic” search using the search term: “Obstructive Sleep *pnea* OR OSAHS OR Upper Airway Resistance Sleep Apnea*”. The asterisk was used to include all different variants of spelling, the Boolean operator “OR” linked the terms to find different synonyms. The bibliometric metadata was saved in an MS Access database and analyzed based on different areas of interest. Here, we demonstrate quantitative and qualitative aspects of the global OSA research over time as well as the international network and geographical developments. Additionally, results were elucidated against the countries’ socioeconomic and scientific background as well as in relation to the countries’ estimated burden. To gain a better understanding of the development of OSA research over time and future prospects, some results were outlined comparatively before (1900 and 2013) and after 2014 (2014 to 2018).

Supporting data of the countries’ scientific- and socioeconomic background were retrieved from the International Monetary Fund (IMF; Washington D.C., USA), the science, technology and innovation report of the UNESCO Institute for Statistics (UIS.Stat; Montreal, Canada), the World Bank (Washington D. C., USA) [23,24] and the WoS InCites Essential Science Indicators [25].

### 2.2. Visualization with Density Equalizing Map Projections

The visualization of the number of publications, the number of citations, the country-specific citation rate, and the modified h-index was realized via DEMPs. They are based on an algorithm of Gastner and Newman generating a distorted depiction of the world map with the sizes of the countries determined by the distribution of a given variable [22].

### 2.3. Risk Factor Evaluation

In the absence of comprehensive global prevalence data, a global risk factor analysis of OSA was performed to gauge the countries’ estimated burden in relation to their actual research activity. Two surrogate parameters were determined to estimate the country-specific risk- and burden of OSA, the country-specific objective multivariable apnea risk index (OMI) [26,27] as well as the country-specific estimated population at risk (EPR) of OSA for countries with ≥300,000 inhabitants. Both parameters were calculated using the most recent available data for the global age- and gender-specific BMI and obesity values and were therefore defined for the year 2013.

#### 2.3.1. Objective Multivariable Apnea Risk Index

The OMI was calculated as: objective MAP index = e^x^/(1 + e^x^) with x = −8.160 + 0.163 × BMI + 0.032 * age + 1.27 * sex (sex: male = 1, female = 0) [26,27]. The country-specific BMI was determined using each country’s weighted mean BMI of the male population age 40 to ≤69 in 2013. The countries’ age- and gender-specific BMI data were obtained from the publication “Trends in adult body-mass index in 200 countries from 1975 to 2014”, generously provided by Majid Ezzati [28]. The weighted mean age of the countries’ male population, age 40 to ≤ 69 was calculated based on data from The World Bank [29]. Overall, the data enabled the identification of the OMI of 186 countries.

#### 2.3.2. Estimated Population at Risk

The country-specific EPR of OSA was determined by calculating each country’s weighted total number of obese, male inhabitants age 40 to ≤69 in 2013. The age- and gender-specific obesity data of the publication “Global, regional, and national prevalence of overweight and obesity in children and adults during 1980–2013”, reported and kindly provided by M. Ng [30], allowed for the calculation of the EPR of 186 countries.

## 3. Results

### 3.1. Progression of OSA Research and Contributing Subject Areas

**Figure 1:**
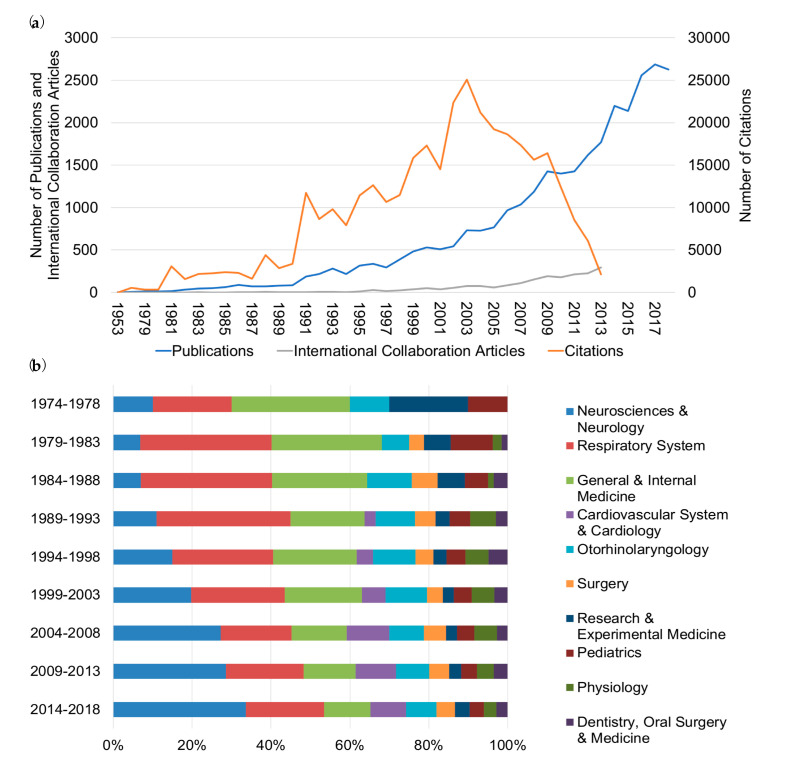
Progression of OSA research and contributing subject areas. (**a**) Total number of OSA-related publications per year between 1953 and 2018 (blue), total number of international collaboration articles per year between 1953 and 2013 (grey), and total number of citations per year between 1953 and 2013 (orange). (**b**) Relative percentages of the ten most productive subject areas publishing on OSA between 1974 and 2018 in intervals of 4 years.

Between 1900 and 2018, a total of 30,853 OSA publications (PUB) were identified, with only 2.1% (PUB = 643) being published before 1991 (Figure 1a; Table S1). From 1991 onwards, the output of publications increased exponentially from 186 to 2626 in 2018. This trend was paralleled by an upswing of international collaboration articles (ICA) and the citation count. The number of ICA rose from 1/year in 1983 to 295/year in 2013, and the citation count increased from 482/year in 1978 to its peak of 25,044/year in 2003 (Figure 1a).

The subsequent decrease in the citation count can be accounted for by the fact of less frequent citations/year of the older publications as well as the more recent publications still being in the process of reaching its peak of awareness within the scientific community. This so-called cited half-life (CHL) is a measure of citation survival reflecting the number of years, going back from the current year, that cover 50% of the citations in the present issue of the journal [31,32]. Between 1900 and 2018, OSA articles were published in 90 different subject areas, with the majority originating from Neurosciences and Neurology (N&N; PUB = 9345), followed by Respiratory System (RS; PUB = 6959), and General and Internal Medicine (G&IM; PUB = 4706) (Figure 1b; Table S2). From 1974 to 2018, we observed a significant increase of the contribution from N&N (from 10 to 34%) and Cardiovascular System and Cardiology (from 3 to 9%), to the detriment of G&IM and RS (Figure 1b).

### 3.2. Global OSA Research

**Figure 2:**
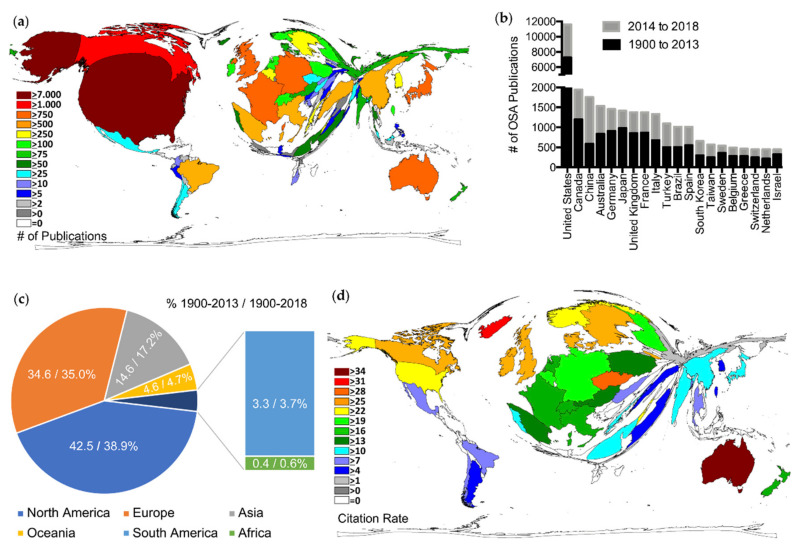
Global OSA research performance. (**a**) Density equalizing map projection of the number of OSA-related publications between 1900 and 2013. A distorted picture of the world map was generated, and the sizes of the countries were determined by their total number of OSA-related publications. (**b**) Top 20 countries in terms of total number of OSA publications between 1900 and 2018; black: publication count from 1900 to 2013; grey: additive publication count from 2014 to 2018. (**c**) Distribution of the global OSA publication output between 1900 and 2018 by continent. Numbers indicate percentage of continents for the period 1900–2013/percentage for the period 1900–2018 (% 1900–2013/% 1900–2018). Antarctica with 0 publications not displayed. (**d**) Density equalizing map projection of the OSA-related country-specific citation rate for the period 1900–2013. A distorted picture of the world map was generated, and the sizes of the countries were determined by their country-specific citation rate (only countries with ≥30 OSA-related articles).

Evaluation of the global OSA research activity from 1900 to 2013 unveiled 88 countries from every continent (except Antarctica) contributing to the overall OSA research, with the USA (PUB = 7272) being the clear leader, followed by Canada (PUB = 1200), Japan (PUB = 980), and Germany (PUB = 908) (Figure 2a).

During this period, the USA was also the central figure regarding the country-specific citations (CIT = 173,883) and modified h-index (HI = 158) and was followed by Canada (CIT = 33,337; HI = 89) and Australia (CIT = 28,896; HI = 72) (Figure S1 and Table S3).

Comparative analysis of the OSA activity before and after 2014 showed a rising global interest, with 33 more countries contributing to the global OSA research (Table S4). Furthermore, several countries exceeded their output during the period 2014–2018 in comparison to the period 1900–2013. Most strikingly, the period 2014–2018 resulted in 1172 publications for China compared to only 586 from 1900 to 2013, making the country now the third highest publishing country in the international research arena (Figure 2b; Table S4). On continent level, this resulted in an increased share for the Asian continent for the period from 1900 to 2018 (14.6% vs. 17.2%), to the detriment of North America (42.5% vs. 38.8%). The share of the remaining continents appeared relatively stable (Figure 2c).

To evaluate the significance and impact of countries’ OSA research within the scientific community, we assessed the country-specific average number of citations per article, citation rate (CR, only countries with ≥30 articles). Here, the DEMP of the country-specific CR for the period 1900–2013 differed significantly from the depiction of the publications, citations, and h-indices, with Australia (CR = 34), Iceland (CR = 32), and the Czech Republic (CR = 29) as leading countries (Figure 2d). In particular, Northern European countries like Iceland, Sweden (CR = 28), and Denmark (CR = 26) were represented more prominently, while the USA (CR = 24) and the most productive European countries, Japan, and China appeared less prominently (Figure 2d).

### 3.3. Global OSA Research Network

**Figure 3:**
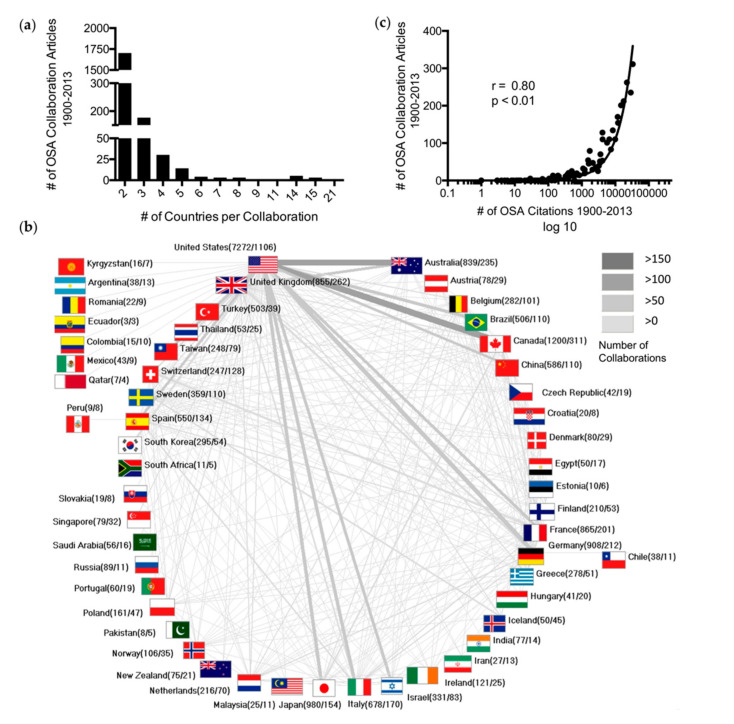
Global OSA research network. (**a**) Number of collaboration articles per number of collaborating countries (period 1900–2013). (**b**) Net diagram of the international OSA collaborations between 1900 and 2013. Line width and grey scale encode the number of collaborations. Digits in parentheses: country-specific number of publications/country-specific number of collaboration articles. (**c**) Correlation between the number of OSA-related international collaboration articles and the number of OSA-related citations between 1900 and 2013. The USA, vastly outnumbering all other countries, was removed in order to provide a better illustration of the remaining countries.

Analysis of the global OSA network (period 1900–2013) identified a total of 1941 ICA, with 87.6% (ICA = 1700) being authored in a bilateral collaboration (Figure 3a).

The USA was the center of the international OSA arena (ICA = 1106), and collaborated most frequently with Canada (n = 129), Australia (n = 113), and Germany (n = 84) (Figure 3b). Furthermore, a significant correlation (*p* < 0.001, r = 0.8) between the countries’ OSA citations and ICA was observed (Figure 3c).

### 3.4. Scientific and Socioeconomic Background

**Figure 4:**
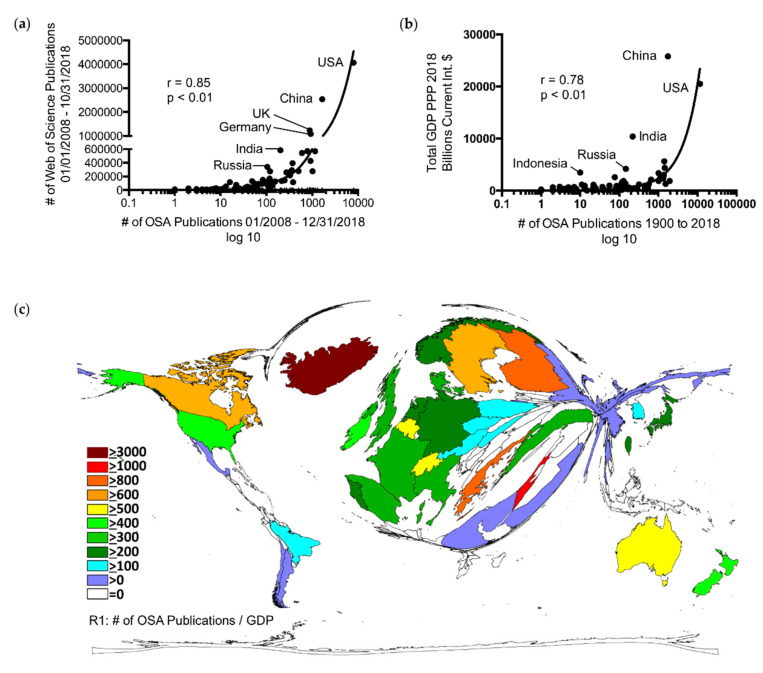
Global OSA research in comparison to scientific and socioeconomic background. (**a**) Comparison of the OSA-related publication output between 1900 and 2018 and the overall publication output of all journals listed in Web of Science (WoS) between January 2008 and October 2018 [25]. (**b**) Correlation between the country-specific OSA publication output between 1900 and 2018 and total Gross Domestic Product (GDP) in 2018 based on Purchasing Power Parity in Billions, current international dollar; source: International Monetary Fund (IMF; Washington D. C., USA) [33]. (**c**) Density equalizing map projection of the country-specific ratio R1 (OSA publication output between 1900 and 2013/total GDP in 2013); source of GDP in 2013: IMF [34]. A distorted picture of the world map was generated, and the sizes of the countries were determined by their respective ratio R1.

A significant correlation was revealed between the country-specific OSA publications (January 2008–31 December 2018) and the overall publications of all journals listed in WoS between January 2008 and 31 October 2018 (r = 0.85, *p* < 0.001) (Figure 4a) [25].

However, China, India, and Russia clearly ranked higher with respect to the overall scientific output as compared to their OSA output (Figure 4a).

Furthermore, the country-specific OSA parameter (PUB, CIT, HI, and ICA) correlated significantly (*p* < 0.001, respectively) with the economic parameters (Gross Domestic Product (GDP) and Research & Development Expenditure (R&D) in purchasing power parity (PPP) for normalization) (Figure 4b; Figures S2–S5) [24,33,34,35].

However, China, India, and Russia exhibited relatively low OSA performances compared to their economic situation (Figure 4b). Gauging of the country-specific investment in OSA research via the country-specific ratios R1 (OSA publications 1900–2018/total GDP) and R2 (OSA publications 1900–2018/total R&D) disclosed a dominance of countries with low absolute numbers of OSA publications (only countries with ≥30 articles). The country upfront was Iceland, followed by Greece and Israel (Figure 4c, Figure S6). On the contrary, we found a relatively low ranking of the USA and the most productive European countries, with China even appearing last in this comparison (Figure 4c; Table S5 and Figure S6).

### 3.5. Epidemiological Influences

**Figure 5:**
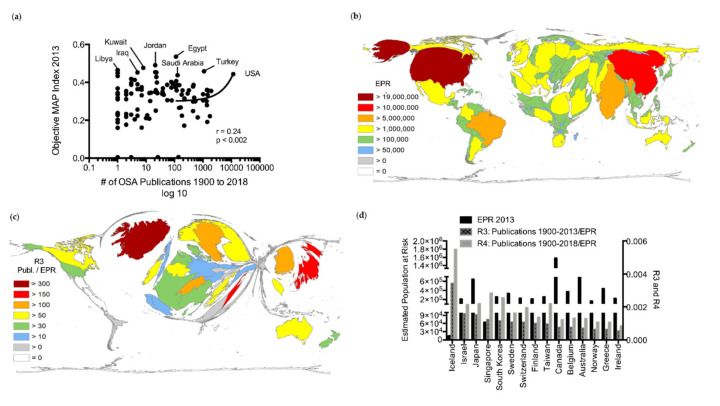
Epidemiological influences. (**a**) Correlation between the country-specific OSA publication output between 1900 and 2018 and objective multivariable apnea risk index (objective MAP Index) in 2013 (only countries ≥300,000 inhabitants) [26,27,28,29]. (**b**) Density equalizing map projection of the estimated population at risk of OSA in 2013 (total number of the countries’ obese, male population, age 40 to ≤69 in 2013; only countries with ≥300,000 inhabitants) [30]. A distorted picture of the world map was generated, and the sizes of the countries were determined by the distribution of their estimated population at risk (EPR) in 2013. (**c**) Density equalizing map projection of the ratio R3 (OSA publication output between 1900 and 2013/estimated population at risk per 100,000 in 2013 for countries with ≥300,000 inhabitants). A distorted picture of the world map was generated, and the sizes of the countries were determined by their respective ratio R3 [30]. (**d**) Comparison of the country-specific ratios R3 (OSA publication output 1900–2018/estimated population at risk) and R4 in relation to the estimated population at risk. Displayed are the top 15 countries in terms of country-specific ratio R3. Black: estimated population at risk in 2013; grey with black fill: country-specific ratio R3; grey: country-specific ratio R4.

As an indicator for the country-specific risk of OSA, we determined the OMI (Tables S6–S8) [26,27,28]. Noticeably, countries with only low research activities exhibited the highest OMIs, particularly Middle Eastern countries such as Egypt (OMI = 0.54), Jordan (OMI = 0.49), Kuwait (OMI = 0.48), and Libya (OMI = 0.47; Figure 5a; Table S8).

Furthermore, only a weak association was found between the OMI and the OSA output (r = 0.24, *p* = 0.002) (Figure 5a). In addition, to assess the countries’ estimated absolute burden of OSA, the country-specific EPR was determined (Table S9) [30]. Countries with the highest EPR were the USA (EPR = 1.9 × 10^7^), China (EPR = 1.2 × 10^7^), India (EPR = 8.0 × 10^6^), and Brazil (EPR = 5.81 × 10^6^) (Figure 5b). Although observing a significant correlation between the OSA publications and the EPR (r = 0.76, *p* < 0.001), China, India, Brazil, Russia, and Mexico displayed only relatively limited OSA performances (Figure S7a).

Additionally, when comparing the EPR to the most recent estimates by Benjafield et al. (country-specific total number of affected individuals with an AHI > 15/h) based on the extrapolation of data from existing prevalence studies (16 countries) [17], a significant correlation was revealed (r = 0.85, *p* < 0.001) (Figure S7b). Furthermore, a similar set of outliers (China, India, Brazil, and Russia) was displayed when correlating the countries’ total publication output generated in our study and the estimates by Benjafield et al. (r = 0.56, *p* < 0.001) (Figure S7c).

To gauge the countries’ OSA awareness/expertise in relation to their EPR and to estimate developments of the country-specific efforts in OSA research in the recent years, the country-specific ratios R3 (OSA publications from 1900–2013/EPR) and R4 (OSA publications 1900–2018/EPR) were evaluated (Figure 5c,d; Table S10). Several countries significantly increased their output from 2014 to 2018, most strikingly Singapore and South Korea, while Iceland remained in the leading position regarding R3 and R4 (Figure 5d; Table S10). On the contrary, comparative analysis of R3 and R4 showed a continuous mismatch of the estimated burden and actual research performance in countries with the highest EPRs, specifically USA, Brazil, China, Russia, and India (Figure 5c,d; Figure S7d and Table S10).

## 4. Discussion

Our study provides the first in-depth presentation of the global OSA research architecture in relation to the estimated risk- and burden of OSA, as well as to socioeconomic and scientific characters of the publishing countries. Beginning in 1965, with the first polysomnographic description of OSA [36], and with the first presentation of an effective treatment [continuous positive airway pressure therapy (CPAP)] in 1981 [37], the rapid scientific advances in the field of sleep medicine [38] are reflected in an exponential rise of the OSA publication activity after 1990/91. Similarly, following the recognition of OSA as a chronic, complex disease requiring multidisciplinary management in 1976 [39], an increasing variety of subject areas dedicated to OSA research were revealed. The improved perception of the sequelae and interplay between OSA and the cardiovascular system was reflected in a significant increase of articles originating from the subject area Cardiovascular System and Cardiology, which is in line with previous findings [40]. In accordance with previous bibliometric studies of various research areas [41,42], the USA was identified as the leading country within the international research arena. Analogously to the significant increase of publications co-authored in international collaboration throughout biomedical research [43], a strong rise in international collaborations on OSA was observed. In line with growing evidence of the importance of international collaborations for the countries’ scientific resonance [44], countries with well-established international OSA networks, in particular, displayed high citation counts and modified h-indices. On the contrary, low OSA publication continents such as Africa, major parts of South America, and Asia were rarely involved in international collaborations. Consequently, especially low-resource nations could benefit from sharing resources with the more experienced and well-equipped protagonists. It could further serve as an impetus for domestic research efforts in the respective countries as well as an opportunity to gain valuable insights into the regional epidemiology of OSA.

As expected, the countries’ OSA research correlated significantly with their overall scientific efforts [25], and the countries’ economic strength was identified as a critical factor in OSA output, quality, and international resonance. However, China, India, and Russia exhibited relatively low OSA performances in comparison to their economic strength and overall scientific performance indicative for a reduced priority to invest in OSA research. This may be attributed to the fact that sleep medicine is still a young discipline, and the understanding of OSA and its adverse outcomes as a major health problem has arisen only recently. Diagnosis and treatment of sleep disorders is technically complex and cost-intensive, which further explains why sleep disorders did not find their way to the top of the public health agendas in the most populous nations of the world, which already have overburdened health systems. Conversely, despite its low absolute publication output, Iceland appeared to have the highest investment in OSA research compared to its economic strength (R1 and R2). The Icelandic Sleep Research Society (Reykjavik, Iceland), already founded in 1991, focused early on enhancing sleep research and on actively fostering international collaborations, mainly with Sweden, Finland, and the USA [45]. High rankings in terms of the CR and h-index support its renowned position within the international OSA community.

Despite globally rising numbers of OSA patients and the impact of OSA on economies and health systems, comprehensive prevalence data are still lacking for many countries [17,46]. Furthermore, available studies have limitations due to methodological concerns, such as not-randomly selected populations, variable definitions of disease, as well as varying distributions of risk factors in the population being studied [2]; the most recent estimate of the global OSA prevalence is based on an extrapolation of data from only 16 countries [17]. Generally, obesity, male gender, and age are considered to be the strongest risk factors for OSA [47], and an increase of the body mass index (BMI) is linked to an increase of the prevalence, regardless of the underlying ethnicity [12]. Thus, we used the most recent globally available age-, gender-, and country-specific obesity, as well as BMI data to create comprehensive surrogate markers estimating a reliable global landscape of the risk- (OMI) and absolute burden (EPR) of OSA. Overall, our analysis revealed an estimated 118.59 million male individuals with a high risk of OSA. In comparison, an estimate of the WHO in 2007 reported approximately 100 million individuals (males and females) to be affected by sleep disordered breathing, and the most recent extrapolation of Benjafield et al. in 2019 stated that roughly 425 million individuals (males and females, age 30–69) suffered from moderate to severe OSA (AHI > 15/h) [17,48]. However, precise specification of the estimated burden remains highly challenging due to the paucity of global epidemiologic data and the high variance in the underlying phenotypes [49].

Within the group of countries with a high OMI, countries of the Middle East were strongly overrepresented; however, these countries exhibited only very poor to non-existent research activities. Similarly, the majority of countries with the highest EPR also had very weak OSA performances; these were predominantly developing countries [49]. Accordingly, the comparison of the countries’ OSA publication count to the most recent estimate of the number of affected individuals with an AHI > 15/h by Benjafield et al. revealed a similar mismatch regarding the estimated OSA burden and OSA research performance. Hence, this discrepancy stresses the scarcity of awareness, infrastructure, and trained human resources for sleep disorders, especially in developing countries [50]. Due to the global upsurge of noncommunicable diseases such as obesity and diabetes and their interrelations to OSA [51], those nations, already troubled by malnutrition and infectious diseases, are currently facing an additional strain on their public health systems [52]. Furthermore, while our data suggest a strong association between the economic strength and the OSA activity on country level, recent studies have also taken into account the complex interplay between the individual socioeconomic status (SES) and sleep disorders. A growing body of literature of different population subgroups [53] is elucidating the role of a low SES on sleep outcomes such as insomnia, excessive daytime sleepiness, and sleep efficiency [54,55]. However, to date, only limited studies have evaluated the associations between SES and OSA. The existing data, mostly generated in the USA, indicate a significant relation between low SES and increasing risk for OSA [56]. Looking forward, further research in this field, including longitudinal studies [57] and inclusion of different countries, may help gaining a deeper understanding of the complex impact of the SES on OSA severity and phenotype.

However, our study also revealed a large imbalance between the estimated burden and actual OSA performance (R3 and R4) for several of the developed nations highly active in OSA research. This is in line with recent studies showing that OSA still remains underdiagnosed/untreated even in highly industrialized nations due to a mismatch between the growing demand and actual capacity, causing long wait times [58,59,60,61]. Consequently, since costs linked to undiagnosed OSA outweigh by far the costs of treating OSA, this discrepancy further increases the economic burden for healthcare systems worldwide [19,62]. Overall, in light of the increasing recognition of the heterogeneity of OSA depending on age, sex, race/ethnicity, as well as socioeconomic factors [18,57], and given the fact that the majority of epidemiologic studies on OSA focus around middle-aged white men from industrial nations, future studies depicting the actual epidemiologic aspects and identifying the individual needs in terms of diagnosis and treatment of the respective phenotype are needed. For instance, growing evidence is showing that Asians suffer from greater severity of OSA despite lower BMI levels compared to Caucasians, mainly due to differences in adipose tissue distribution and craniofacial structure [63]. Consequently, the here-presented mismatch between scientific activity and estimated burden, particularly in Asian countries, might even be higher. In fact, the present one-size-fits-all approach in the diagnosis and management of OSA is currently being questioned, and efforts towards comprehensive personalized medicine including concepts such as genetics, biomarkers, and phenotypic approaches are being driven forward [18].

Finally, comparative analysis of the OSA research output before and after 2014 identified several countries with significantly growing investment in OSA research. China and India, in particular, were massively increasing their OSA publication output. However, these improvements only resulted in small effects in relation to their estimated OSA burden (R4) and further emphasize the persistent discrepancy between the estimated demand and actual OSA expertise.

## 5. Conclusions

Global OSA research has evolved over time; however, our findings demonstrate considerably differing scientific activities depending on country and region. While the global OSA activity strongly correlated with the countries’ overall scientific activity and financial strength, we unveiled major discrepancies between the estimated country-specific burden (demand) and actual scientific activity not limited to only low resource nations. Hence, the rising prevalence of OSA and the increasing evidence of its high variability in terms of age, sex, and race/ethnicity urges a rethinking of the current strategies for approaching OSA. To satisfy the needs of the heterogeneous world population and its diverse characteristics of OSA, novel and individual approaches are necessary to not only identify the true burden, but to also establish adequate approaches in the diagnosis and treatment of OSA. Furthermore, our study demonstrated that being integrated in the international scientific community positively affects a country’s scientific resonance/performance. Particularly low-resource nations, already double-burdened by malnutrition/infectious diseases and increasing noncommunicable risk factors such as obesity and diabetes, could benefit from being integrated in the international OSA research arena. Consequently, this could promote awareness of OSA in those disadvantaged nations, providing the ground and resources for establishing and facilitating OSA research efforts and leveraging health initiatives. Ultimately, this could gain novel and unique insights into the regional characteristics of OSA in terms of prevalence, management, and prognosis and could serve as basis for a global network enabling surveillance and registries.

## 6. Limitations

Based on the nature of our approach, our study has some limitations. Using the WoS as a data source carries the risk of an underrepresentation of non-English articles, since the WoS core collection is dominated by journals publishing in English. However, English is established as the universal language of science [64], and high-quality research is nearly exclusively published in English-language journals. Furthermore, use of citation counts as a measure of scientific quality might be biased based on the Matthew Effect, where publications by prestigious scientists are cited more often than articles by less recognized researchers [65].

In the absence of comprehensive global prevalence data, we chose to assess the countries’ risk/burden of OSA based on the major risk factors obesity, male gender, and age between 40 and 69. By determining the countries’ objective MAP index and the estimated population at risk, we did not aim to precisely reflect the countries’ risk/prevalence, but the overriding interest was in generating the most comprehensive and robust dataset, enabling an objective comparison across all countries worldwide, independent of limitations such as methodological concerns, not-randomly selected populations, or a lack of the country-specific expertise. Consequently, due to the limited age range, the inclusion of only the male gender, and the recent development of the obesity pandemic, the overall estimated risk and burden is presumably underestimated. Furthermore, although obesity is assumed to be the strongest risk factor for OSA in all ethnic groups, increasing evidence of significant differences in OSA prevalence and severity among different ethnicities mainly due to craniofacial factors and different distribution of adipose tissue [66] may contribute to an underestimation in certain ethnicities. Lastly, our data do not allow any conclusions to be drawn as to the underlying severity of OSA, since no scoring criteria of any kind were integrated in our risk estimation.

## Supplementary Materials

The following are available online at https://www.mdpi.com/1660-4601/17/18/6785/s1: Table S1: Progression of OSA research., Table S2: Subject areas contributing to OSA research., Table S3: Global OSA research performance from 1900–2013., Table S4: Trend—Development of the global OSA activity from 2014 to 2018., Table S5: Global OSA publications from 1900 to 2018 in relation to economic parameters., Table S6: Percentage and weighted mean age of male population age 40 to ≤ 69 in 2013., Table S7: Country-specific mean Body Mass Index (BMI) and weighted mean BMI of male population age 40 to ≤ 69 in 2013., Table S8: Objective Multivariable Apnea Risk Index (objective MAP index) of male population age 40 ≤ 69 in 2013., Table S9: Country-specific Estimated Population at Risk (EPR) for OSA in 2013., Table S10: Country-specific ratios R3 and R4. Figure S1: Global OSA-related citations and modified h-indices., Figure S2: Global OSA publication output in relation to research & development expenditure., Figure S3: Global OSA citation count in relation to economic parameters., Figure S4: Global OSA-related h-indices in relation to economic parameters., Figure S5: Economic background of international OSA collaborations., Figure S6: Ratio R1 in comparison to publication activity., Figure S7: Epidemiological influences.
