# Supplementary material for: Global Risk Factor Evaluation of Obstructive Sleep Apnea in Relation to Research Activity and Socioeconomic Factors
Source: Int J Environ Res Public Health. 2020 Sep 17;17(18):6785. doi: 10.3390/ijerph17186785 (PMC7559375; doi:10.3390/ijerph17186785)
Supplement: Supplementary file 1 [file ijerph-17-06785-s001.pdf]

Supplementary Information

Global Risk Factor Evaluation of Obstructive Sleep Apnea in Relation to Research Activity and Socioeconomic Factors

Rebekka K. Seeger-Zybok *, Doris Klingelhöfer and David A. Groneberg

Institute of Occupational, Social and Environmental Medicine, Goethe University of Frankfurt, 60590 Frankfurt, Germany; klingelhoefer@med.uni-frankfurt.de (D.K.); groneberg@med.uni-frankfurt.de (D.A.G.)

* Correspondence: rebekkaseeger@gmail.com; Tel.: +49-69-6301-6650 (R.K.S.-Z.)

Supplementary Tables

Table S1 - Progression of OSA research.

Evolution of OSA publications from 1900 to 2018 and evolution of OSA citations as well as collaboration articles from 1900 to 2013.

Year	# of Publications / Year	# of Citations / Year	# of Collaboration Articles / Year
1953	1	NA	0
1978	8	482	0
1979	12	311	0
1980	13	347	0
1981	16	3,086	0
1982	34	1,580	0
1983	47	2,167	1
1984	49	2,242	0
1985	61	2,398	0
1986	89	2,292	1
1987	73	1,592	1
1988	73	4,418	5
1989	79	2,837	2
1990	84	3,381	2
1991	185	11,744	3
1992	219	8,627	9
1993	282	9,810	7
1994	218	7,929	3
1995	315	11,416	13
1996	337	12,648	27
1997	294	10,643	14
1998	388	11,487	22
1999	481	15,855	36
2000	531	17,284	48
2001	507	14,501	37
2002	541	22,383	52
2003	733	25,044	75
2004	729	21,211	77
2005	766	19,250	59
2006	969	18,641	84
2007	1,034	17,339	108
2008	1,185	15,618	151

2009	1,427	16,418	193
2010	1,402	12,378	179
2011	1,425	8,529	212
2012	1,620	6,073	225
2013	1,768	2,126	295
2014	2,199	N/A	N/A
2015	2,139	N/A	N/A
2016	2,559	N/A	N/A
2017	2,686	N/A	N/A
2018	2,626	N/A	N/A

Table S2 - Subject areas contributing to OSA research.

Subject areas publishing on OSA between 1900 and 2013, sorted by number of OSA publications in descending order.

	Subject Area	# of OSA Publications	# of OSA Citations	Ranking regarding # of Citations	Citation Rate	Ranking regarding Citation Rate
1	Neurosciences & Neurology	4,720	68,299	3	14	19
2	Respiratory System	4,236	107,565	1	25	4
3	General & Internal Medicine	3,104	88,421	2	28	2
4	Otorhinolaryngology	1,813	23,998	6	13	22
5	Cardiovascular System & Cardiology	1,661	38,019	4	23	5
6	Physiology	994	26,776	5	27	3
7	Surgery	982	16,208	8	17	11
8	Paediatrics	864	18,712	7	22	7
9	Dentistry, Oral Surgery & Medicine	665	8,139	12	12	24
10	Research & Experimental Medicine	616	10,308	10	17	11
11	Sport Sciences	457	13,849	9	30	1
12	Endocrinology & Metabolism	410	8,742	11	21	8
13	Anaesthesiology	341	5,004	13	15	18
14	Psychiatry	299	4,273	14	14	19
15	Pharmacology & Pharmacy	252	2,238	18	9	33
16	Engineering	228	2,133	19	9	33
17	Urology & Nephrology	174	1,898	21	11	25
18	Gastroenterology & Hepatology	152	2,805	15	18	10
19	Nutrition & Dietetics	131	2,764	16	21	8

20	Public, Environmental & Occupational Health	124	2,121	20	17	11
21	Psychology	109	2,456	17	23	5
22	Radiology, Nuclear Medicine & Medical Imaging	108	1,511	23	14	19
23	Science & Technology - Other Topics	97	615	33	6	37
24	Biochemistry & Molecular Biology	95	1,658	22	17	11
25	Obstetrics & Gynaecology	92	798	30	9	33
26	Ophthalmology	80	766	32	10	30
27	Nursing	76	230	40	3	38
28	Geriatrics & Gerontology	75	850	28	11	25
29	Cell Biology	74	818	29	11	25
29	Health Care Sciences & Services	74	774	31	10	30
31	Immunology	72	1,140	24	16	16
31	Allergy	72	946	25	13	22
33	Life Sciences & Biomedicine - Other Topics	70	575	34	8	36
34	Genetics & Heredity	57	936	27	16	16
35	Haematology	54	941	26	17	11
36	Medical Informatics	53	532	35	10	30
37	Rehabilitation	44	500	36	11	25
38	Mathematical & Computational Biology	42	474	37	11	25
39	Biophysics	29	455	38	0	40
40	Pathology	27	207	41	0	40
41	Transplantation	26	187	44	0	40
42	Rheumatology	23	195	43	0	40

43	Medical Laboratory Technology	22	134	48	0	40
44	Computer Science	19	123	49	0	40
45	Toxicology	18	170	46	0	40
46	Orthopaedics	16	77	53	0	40
47	Behavioural Sciences	15	237	39	0	40
47	Veterinary Sciences	15	198	42	0	40
49	Reproductive Biology	12	171	45	0	40
49	Anatomy & Morphology	12	78	52	0	40
49	Dermatology	12	45	65	0	40
52	Oncology	10	53	59	0	40
52	Mathematics	10	39	67	0	40
52	Legal Medicine	10	37	68	0	40
52	Operations Research & Management Science	10	37	68	0	40
56	Infectious Diseases	9	170	46	0	40
56	Emergency Medicine	9	50	62	0	40
58	Biotechnology & Applied Microbiology	8	56	58	0	40
59	Medical Informatics	7	115	51	0	40
59	Physics	7	47	64	0	40
59	Instruments & Instrumentation	7	23	73	0	40
62	Integrative & Complementary Medicine	6	13	74	2	39
62	Substance Abuse	6	123	49	0	40
62	Tropical Medicine	6	9	77	0	40
65	Optics	5	64	56	0	40
65	Women Studies	5	61	57	0	40
65	Developmental Biology	5	51	61	0	40

65	Chemistry	5	48	63	0	40
65	Environmental Sciences & Ecology	5	4	82	0	40
65	Business & Economics	5	0	85	0	40
71	Mechanics	4	66	55	0	40
71	Transportation	4	40	66	0	40
71	Microbiology	4	34	70	0	40
71	Acoustics	4	31	71	0	40
71	Social Sciences - Other Topics	4	27	72	0	40
71	Education & Educational Research	4	5	80	0	40
71	Materials Science	4	2	83	0	40
78	History & Philosophy of Science	2	74	54	0	40
78	Microscopy	2	12	75	0	40
78	Audiology & Speech-Language Pathology	2	8	79	0	40
78	Electrochemistry	2	5	80	0	40
78	Water Resources	2	0	85	0	40
83	Zoology	1	52	60	0	40
83	Robotics	1	11	76	0	40
83	Spectroscopy	1	9	77	0	40
83	Virology	1	2	83	0	40
83	Linguistics	1	0	85	0	40
83	Telecommunications	1	0	85	0	40
83	Mathematical Methods In Social Sciences	1	0	85	0	40
83	Evolutionary Biology	1	0	85	0	40
83	Metallurgy & Metallurgical Engineering	1	0	85	0	40

	Food Science & Technology	0	0		0	
	Plant Sciences	0	0		0	
	Construction & Building Technology	0	0		0	
	Government & Law	0	0		0	
	Anthropology	0	0		0	

Table S3 - Global OSA research performance from 1900-2013.

Distribution of the global OSA related publications, citations, country-specific citation rate, and modified h-indices from 1900 to 2013, sorted by number of OSA publications in descending order.

	Country	# of OSA Publications	# of OSA Citations	Ranking regarding # of Citations	Citation Rate	Ranking regarding Citation Rate	Modified h-index	Ranking regarding Modified h-index
1	United States	7,272	173,883	1	24	14	158	1
2	Canada	1,200	33,337	2	28	7	89	2
3	Japan	980	12,077	7	12	33	51	9
4	Germany	908	17,732	5	20	20	62	5
5	France	865	14,845	6	17	22	61	6
6	United Kingdom	855	22,102	4	26	11	73	3
7	Australia	839	28,896	3	34	4	72	4
8	Italy	678	11,734	8	17	21	57	7
9	China	586	6,641	12	11	36	38	13
10	Spain	550	8,409	10	15	28	46	10
11	Brazil	506	4,160	14	8	48	32	17
12	Turkey	503	3,237	19	6	55	27	20
13	Sweden	359	9,926	9	28	8	56	8
14	Israel	331	7,398	11	22	16	46	10
15	South Korea	295	1,541	26	5	59	21	25
16	Belgium	282	5,679	13	20	18	41	12
17	Greece	278	3,486	18	13	32	34	16
18	Taiwan	248	1,729	24	7	51	24	23
19	Switzerland	247	4,138	16	17	23	36	14
20	Netherlands	216	3,609	17	17	24	29	18
21	Finland	210	4,146	15	20	19	36	14
22	Poland	161	2,145	22	13	30	22	24
23	Ireland	121	3,039	20	25	12	28	19
24	Norway	106	2,621	21	25	13	26	21
25	Russia	89	207	40	2	73	7	42
26	Denmark	80	2,086	23	26	10	26	21
27	Singapore	79	1,092	30	14	29	18	29
28	Austria	78	1,214	28	16	27	20	26
29	India	77	839	31	11	37	16	30
30	New Zealand	75	1,231	27	16	25	20	26
31	Portugal	60	709	32	12	35	12	32
32	Saudi Arabia	56	372	38	7	53	11	35
33	Thailand	53	502	35	9	43	13	31

34	Egypt	50	539	34	11	38	12	32
34	Iceland	50	1,614	25	32	5	19	28
36	Mexico	43	407	36	9	44	12	32
37	Czech Republic	42	1,210	29	29	6	11	35
38	Hungary	41	403	37	10	40	11	35
39	Chile	38	328	39	9	46	11	35
39	Argentina	38	194	42	5	61	11	35
41	Iran	27	138	44	5	60	7	42
42	Malaysia	25	127	45	5	62	6	45
43	Romania	22	47	53	2	75	5	47
44	Croatia	20	81	47	4	64	7	42
45	Slovakia	19	204	41	11	39	8	41
46	Kyrgyzstan	16	20	65	1	78	4	49
47	Colombia	15	33	57	2	74	3	56
48	South Africa	11	106	46	10	41	6	45
49	Estonia	10	607	33	61	1	10	40
50	Philippines	9	76	48	8	47	3	56
50	Slovenia	9	25	62	3	69	3	56
50	Peru	9	23	64	3	71	4	49
50	Lebanon	9	15	66	2	77	4	49
54	Pakistan	8	29	59	4	66	4	49
54	Lithuania	8	26	61	3	67	4	49
56	Qatar	7	67	50	10	42	3	56
56	Oman	7	40	55	6	58	5	47
58	Jordan	6	15	66	3	72	3	56
58	Nigeria	6	11	72	2	76	3	56
58	Bulgaria	6	157	43	26	9	4	49
61	Ukraine	5	33	57	7	54	2	64
61	Tunisia	5	3	78	1	82	2	64
61	United Arab Emirates	5	13	69	3	70	3	56
64	Serbia	4	27	60	7	52	3	56
64	Latvia	4	24	63	6	56	4	49
66	Uruguay	3	48	52	16	26	0	75
66	Indonesia	3	71	49	24	15	2	64
66	Ecuador	3	3	78	1	79	2	64
69	Cyprus	2	14	68	7	50	0	75
69	Moldova	2	0	83	0	83	1	69
69	Venezuela	2	0	83	0	83	1	69

69	Bolivia	2	42	54	21	17	2	64
73	Luxembourg	1	53	51	53	2	0	75
73	Malta	1	38	56	38	3	0	75
73	Morocco	1	13	69	13	31	0	75
73	Libya	1	12	71	12	34	0	75
73	Barbados	1	9	73	9	45	0	75
73	Bahrain	1	8	74	8	49	0	75
73	Grenada	1	6	75	6	56	0	75
73	Martinique	1	5	76	5	63	0	75
73	Albania	1	4	77	4	65	0	75
73	Reunion	1	3	78	3	68	0	75
73	Andorra	1	1	81	1	79	0	75
73	Syria	1	1	81	1	79	0	75
73	French Polynesia	1	0	83	0	83	1	69
73	Georgia	1	0	83	0	83	1	69
73	Kenya	1	0	83	0	83	1	69
73	Tanzania	1	0	83	0	83	1	69
	Afghanistan	0	0		0		0	
	Algeria	0	0		0		0	
	American Samoa	0	0		0		0	
	Angola	0	0		0		0	
	Anguilla	0	0		0		0	
	Antarctica	0	0		0		0	
	Antigua & Barbuda	0	0		0		0	
	Armenia	0	0		0		0	
	Aruba	0	0		0		0	
	Azerbaijan	0	0		0		0	
	Baker I.	0	0		0		0	
	Bangladesh	0	0		0		0	
	Belarus	0	0		0		0	
	Belize	0	0		0		0	
	Benin	0	0		0		0	
	Bermuda	0	0		0		0	
	Bhutan	0	0		0		0	
	Bosnia & Herzegovina	0	0		0		0	
	Botswana	0	0		0		0	
	Bouvet I.	0	0		0		0	

	British Indian Ocean Territory	0	0		0		0	
	British Virgin Is.	0	0		0		0	
	Brunei	0	0		0		0	
	Burkina Faso	0	0		0		0	
	Burundi	0	0		0		0	
	Cambodia	0	0		0		0	
	Cameroon	0	0		0		0	
	Cape Verde	0	0		0		0	
	Cayman Is.	0	0		0		0	
	Central African Republic	0	0		0		0	
	Chad	0	0		0		0	
	Christmas I.	0	0		0		0	
	Cocos Is.	0	0		0		0	
	Comoros	0	0		0		0	
	Congo	0	0		0		0	
	Congo, DRC	0	0		0		0	
	Cook Is.	0	0		0		0	
	Costa Rica	0	0		0		0	
	Cote dIvory	0	0		0		0	
	Cuba	0	0		0		0	
	Djibouti	0	0		0		0	
	Dominica	0	0		0		0	
	Dominican Republic	0	0		0		0	
	East Timor	0	0		0		0	
	El Salvador	0	0		0		0	
	Equatorial Guinea	0	0		0		0	
	Eritrea	0	0		0		0	
	Ethiopia	0	0		0		0	
	Falkland Is.	0	0		0		0	
	Faroe Is.	0	0		0		0	
	Fiji	0	0		0		0	
	French Guiana	0	0		0		0	
	French Southern & Antarctic Lands	0	0		0		0	
	Gabon	0	0		0		0	
	Gaza Strip	0	0		0		0	
	Ghana	0	0		0		0	

	Gibraltar	0	0		0		0	
	Glorioso Is.	0	0		0		0	
	Greenland	0	0		0		0	
	Guadeloupe	0	0		0		0	
	Guam	0	0		0		0	
	Guatemala	0	0		0		0	
	Guernsey	0	0		0		0	
	Guinea	0	0		0		0	
	Guinea-Bissau	0	0		0		0	
	Guyana	0	0		0		0	
	Haiti	0	0		0		0	
	Heard I. & McDonald Is.	0	0		0		0	
	Honduras	0	0		0		0	
	Howland I.	0	0		0		0	
	Iraq	0	0		0		0	
	Isle of Man	0	0		0		0	
	Jamaica	0	0		0		0	
	Jan Mayen	0	0		0		0	
	Jarvis I.	0	0		0		0	
	Jersey	0	0		0		0	
	Johnston Atoll	0	0		0		0	
	Juan De Nova I.	0	0		0		0	
	Kazakhstan	0	0		0		0	
	Kiribati	0	0		0		0	
	Kuwait	0	0		0		0	
	Laos	0	0		0		0	
	Lesotho	0	0		0		0	
	Liberia	0	0		0		0	
	Liechtenstein	0	0		0		0	
	Macedonia	0	0		0		0	
	Madagascar	0	0		0		0	
	Malawi	0	0		0		0	
	Maldives	0	0		0		0	
	Mali	0	0		0		0	
	Marshall Is.	0	0		0		0	
	Mauritania	0	0		0		0	
	Mauritius	0	0		0		0	
	Mayotte	0	0		0		0	

	Micronesia	0	0		0		0	
	Midway Is.	0	0		0		0	
	Monaco	0	0		0		0	
	Mongolia	0	0		0		0	
	Montserrat	0	0		0		0	
	Mozambique	0	0		0		0	
	Myanmar	0	0		0		0	
	Namibia	0	0		0		0	
	Nauru	0	0		0		0	
	Nepal	0	0		0		0	
	Netherlands Antilles	0	0		0		0	
	New Caledonia	0	0		0		0	
	Nicaragua	0	0		0		0	
	Niger	0	0		0		0	
	Niue	0	0		0		0	
	Norfolk I.	0	0		0		0	
	North Korea	0	0		0		0	
	Northern Mariana Is.	0	0		0		0	
	Palau	0	0		0		0	
	Panama	0	0		0		0	
	Papua New Guinea	0	0		0		0	
	Paracel Is.	0	0		0		0	
	Paraguay	0	0		0		0	
	Pitcairn Is.	0	0		0		0	
	Puerto Rico	0	0		0		0	
	Rwanda	0	0		0		0	
	Samoa	0	0		0		0	
	San Marino	0	0		0		0	
	Sao Tome & Principe	0	0		0		0	
	Senegal	0	0		0		0	
	Seychelles	0	0		0		0	
	Sierra Leone	0	0		0		0	
	Solomon Is.	0	0		0		0	
	Somalia	0	0		0		0	
	South Georgia & the South Sandwich Is.	0	0		0		0	

	Spratly Is.	0	0		0		0	
	Sri Lanka	0	0		0		0	
	St. Helena	0	0		0		0	
	St. Kitts & Nevis	0	0		0		0	
	St. Lucia	0	0		0		0	
	St. Pierre & Miquelon	0	0		0		0	
	St. Vincent & the Grenadines	0	0		0		0	
	Sudan	0	0		0		0	
	Suriname	0	0		0		0	
	Svalbard	0	0		0		0	
	Swaziland	0	0		0		0	
	Tajikistan	0	0		0		0	
	The Bahamas	0	0		0		0	
	The Gambia	0	0		0		0	
	Togo	0	0		0		0	
	Tokelau	0	0		0		0	
	Tonga	0	0		0		0	
	Trinidad & Tobago	0	0		0		0	
	Turkmenistan	0	0		0		0	
	Turks & Caicos Is.	0	0		0		0	
	Tuvalu	0	0		0		0	
	Uganda	0	0		0		0	
	Uzbekistan	0	0		0		0	
	Vanuatu	0	0		0		0	
	Vatican City	0	0		0		0	
	Vietnam	0	0		0		0	
	Virgin Is.	0	0		0		0	
	Wake I.	0	0		0		0	
	Wallis & Futuna	0	0		0		0	
	West Bank	0	0		0		0	
	Western Sahara	0	0		0		0	
	Yemen	0	0		0		0	
	Zambia	0	0		0		0	
	Zimbabwe	0	0		0		0	

Table S4 - Trend – Development of the global OSA activity from 2014 to 2018.

Comparison of the global OSA publication output between 1900-2013 and 1900-2018, sorted by publication output from 1900 to 2018.

	Country	# of OSA Publications 1900 to 2018	# of OSA Publications 1900 to 2013	Ranking regarding # of Publications 1900 to 2013	# of OSA Publications 2014 to 2018	% of # Publications 2014-2018 of Overall # of Publications
1	United States	11,611	7,272	1	4,339	37
2	Canada	1,947	1,200	2	747	38
3	China	1,758	586	9	1,172	67
4	Australia	1,534	839	7	695	45
5	Germany	1,456	908	4	548	38
6	Japan	1,413	980	3	433	31
7	United Kingdom	1,375	855	6	520	38
8	France	1,374	865	5	509	37
9	Italy	1,329	678	8	651	49
10	Turkey	1,093	503	12	590	54
11	Brazil	1,015	506	11	509	50
12	Spain	1,011	550	10	461	46
13	South Korea	654	295	15	359	55
14	Taiwan	567	248	18	319	56
15	Sweden	539	359	13	180	33
16	Belgium	492	282	16	210	43
17	Greece	466	278	17	188	40
18	Switzerland	450	247	19	203	45
18	Netherlands	450	216	20	234	52
20	Israel	448	331	14	117	26
21	Poland	321	161	22	160	50
22	Finland	289	210	21	79	27
23	Portugal	223	60	31	163	73
24	India	219	77	29	142	65
25	Ireland	191	121	23	70	37
26	Singapore	183	79	27	104	57
27	Norway	179	106	24	73	41
28	Denmark	164	80	26	84	51
29	Russia	147	89	25	58	39
30	Thailand	145	53	33	92	63
31	Austria	137	78	28	59	43
32	Saudi Arabia	128	56	32	72	56

33	Iran	119	27	41	92	77
34	New Zealand	118	75	30	43	36
35	Chile	114	38	39	76	67
36	Egypt	113	50	34	63	56
37	Romania	112	22	43	90	80
38	Czech Republic	108	42	37	66	61
39	Argentina	85	38	40	47	55
40	Iceland	80	50	35	30	38
41	Hungary	78	41	38	37	47
42	Mexico	77	43	36	34	44
43	Malaysia	56	25	42	31	55
44	Croatia	55	20	44	35	64
45	Slovakia	42	19	45	23	55
46	Tunisia	39	5	61	34	87
47	Serbia	38	4	64	34	89
48	Colombia	37	15	47	22	59
49	Bulgaria	26	6	58	20	77
50	Peru	25	9	50	16	64
51	Lebanon	24	9	51	15	63
52	South Africa	23	11	48	12	52
52	Lithuania	23	8	54	15	65
52	United Arab Emirates	23	5	62	18	78
55	Kyrgyzstan	22	16	46	6	27
55	Pakistan	22	8	55	14	64
57	Slovenia	21	9	52	12	57
57	Qatar	21	7	56	14	67
57	Jordan	21	6	59	15	71
60	Estonia	15	10	49	5	33
61	Oman	14	7	57	7	50
62	Philippines	13	9	53	4	31
62	Ukraine	13	5	63	8	62
64	Nigeria	11	6	60	5	45
65	Indonesia	10	3	66	7	70
66	Kuwait	8	-		8	-
67	Ecuador	7	3	67	4	57
67	Cyprus	7	2	69	5	71
67	Morocco	7	1	73	6	86
67	Vietnam	7	-		7	-
71	Latvia	6	4	65	2	33

72	Uruguay	5	3	68	2	40
72	Bolivia	5	2	70	3	60
72	Cameroon	5	-		5	-
72	Iraq	5	-		5	-
72	Sri Lanka	5	-		5	-
77	Moldova	4	2	71	2	50
77	Guadeloupe	4	-		4	-
77	Myanmar	4	-		4	-
80	Venezuela	3	2	72	1	33
80	Georgia	3	1	74	2	67
80	Kenya	3	1	75	2	67
80	Malta	3	1	76	2	67
80	Bangladesh	3	-		3	-
80	Belarus	3	-		3	-
80	Liechtenstein	3	-		3	-
87	Albania	2	1	77	1	50
87	Bahrain	2	1	78	1	50
87	French Polynesia	2	1	79	1	50
87	Grenada	2	1	80	1	50
87	Luxembourg	2	1	81	1	50
87	Tanzania	2	1	82	1	50
87	Algeria	2	-		2	-
87	Kazakhstan	2	-		2	-
87	Montenegro	2	-		2	-
87	Nepal	2	-		2	-
97	Andorra	1	1	83	-	-
97	Barbados	1	1	84	-	-
97	Libya	1	1	85	-	-
97	Martinique	1	1	86	-	-
97	Reunion	1	1	87	-	-
97	Syria	1	1	88	-	-
97	Armenia	1	-	-	1	-
97	Aruba	1	-	-	1	-
97	Benin	1	-	-	1	-
97	Bosnia & Herzegovina	1	-	-	1	-
97	Burkina Faso	1	-	-	1	-
97	Congo	1	-	-	1	-
97	Cuba	1	-	-	1	-
97	Jamaica	1	-	-	1	-

97	Macedonia	1	-	-	1	-
97	Malawi	1	-	-	1	-
97	Mozambique	1	-	-	1	-
97	Netherlands Antilles	1	-	-	1	-
97	New Caledonia	1	-	-	1	-
97	Paraguay	1	-	-	1	-
97	San Marino	1	-	-	1	-
97	St. Kitts & Nevis	1	-	-	1	-
97	Swaziland	1	-	-	1	-
97	Uzbekistan	1	-	-	1	-
97	Yemen	1	-	-	1	-
-	Afghanistan	-	-	-	-	
-	American Samoa	-	-	-	-	
-	Angola	-	-	-	-	
-	Anguilla	-	-	-	-	
-	Antarctica	-	-	-	-	
-	Antigua & Barbuda	-	-	-	-	
-	Azerbaijan	-	-	-	-	
-	Baker I.	-	-	-	-	
-	Belize	-	-	-	-	
-	Bermuda	-	-	-	-	
-	Bhutan	-	-	-	-	
-	Botswana	-	-	-	-	
-	Bouvet I.	-	-	-	-	
-	British Indian Ocean Territory	-	-	-	-	
-	British Virgin Is.	-	-	-	-	
-	Brunei	-	-	-	-	
-	Burundi	-	-	-	-	
-	Cambodia	-	-	-	-	
-	Cape Verde	-	-	-	-	
-	Cayman Is.	-	-	-	-	
-	Central African Republic	-	-	-	-	
-	Chad	-	-	-	-	
-	Christmas I.	-	-	-	-	
-	Cocos Is.	-	-	-	-	
-	Comoros	-	-	-	-	
-	Congo, DRC	-	-	-	-	
-	Cook Is.	-	-	-	-	
-	Costa Rica	-	-	-	-	

-	Cote dIvory	-	-	-	-	
-	Djibouti	-	-	-	-	
-	Dominica	-	-	-	-	
-	Dominican Republic	-	-	-	-	
-	East Timor	-	-	-	-	
-	El Salvador	-	-	-	-	
-	Equatorial Guinea	-	-	-	-	
-	Eritrea	-	-	-	-	
-	Ethiopia	-	-	-	-	
-	Falkland Is.	-	-	-	-	
-	Faroe Is.	-	-	-	-	
-	Fiji	-	-	-	-	
-	French Guiana	-	-	-	-	
-	French Southern & Antarctic Lands	-	-	-	-	
-	Gabon	-	-	-	-	
-	Gaza Strip	-	-	-	-	
-	Ghana	-	-	-	-	
-	Gibraltar	-	-	-	-	
-	Glorioso Is.	-	-	-	-	
-	Greenland	-	-	-	-	
-	Guam	-	-	-	-	
-	Guatemala	-	-	-	-	
-	Guernsey	-	-	-	-	
-	Guinea	-	-	-	-	
-	Guinea-Bissau	-	-	-	-	
-	Guyana	-	-	-	-	
-	Haiti	-	-	-	-	
-	Heard I. & McDonald Is.	-	-	-	-	
-	Honduras	-	-	-	-	
-	Howland I.	-	-	-	-	
-	Isle of Man	-	-	-	-	
-	Jan Mayen	-	-	-	-	
-	Jarvis I.	-	-	-	-	
-	Jersey	-	-	-	-	
-	Johnston Atoll	-	-	-	-	
-	Juan De Nova I.	-	-	-	-	
-	Kiribati	-	-	-	-	
-	Laos	-	-	-	-	
-	Lesotho	-	-	-	-	

-	Liberia	-	-	-	-	
-	Madagascar	-	-	-	-	
-	Maldives	-	-	-	-	
-	Mali	-	-	-	-	
-	Marshall Is.	-	-	-	-	
-	Mauritania	-	-	-	-	
-	Mauritius	-	-	-	-	
-	Mayotte	-	-	-	-	
-	Micronesia	-	-	-	-	
-	Midway Is.	-	-	-	-	
-	Monaco	-	-	-	-	
-	Mongolia	-	-	-	-	
-	Montserrat	-	-	-	-	
-	Namibia	-	-	-	-	
-	Nauru	-	-	-	-	
-	Nicaragua	-	-	-	-	
-	Niger	-	-	-	-	
-	Niue	-	-	-	-	
-	Norfolk I.	-	-	-	-	
-	North Korea	-	-	-	-	
-	Northern Mariana Is.	-	-	-	-	
-	Palau	-	-	-	-	
-	Panama	-	-	-	-	
-	Papua New Guinea	-	-	-	-	
-	Paracel Is.	-	-	-	-	
-	Pitcairn Is.	-	-	-	-	
-	Puerto Rico	-	-	-	-	
-	Rwanda	-	-	-	-	
-	Samoa	-	-	-	-	
-	Sao Tome & Principe	-	-	-	-	
-	Senegal	-	-	-	-	
-	Seychelles	-	-	-	-	
-	Sierra Leone	-	-	-	-	
-	Solomon Is.	-	-	-	-	
-	Somalia	-	-	-	-	
-	South Georgia & the South Sandwich Is.	-	-	-	-	
-	Spratly Is.	-	-	-	-	
-	St. Helena	-	-	-	-	
-	St. Lucia	-	-	-	-	

-	St. Pierre & Miquelon	-	-	-	-	
-	St. Vincent & the Grenadines	-	-	-	-	
-	Sudan	-	-	-	-	
-	Suriname	-	-	-	-	
-	Svalbard	-	-	-	-	
-	Tajikistan	-	-	-	-	
-	The Bahamas	-	-	-	-	
-	The Gambia	-	-	-	-	
-	Togo	-	-	-	-	
-	Tokelau	-	-	-	-	
-	Tonga	-	-	-	-	
-	Trinidad & Tobago	-	-	-	-	
-	Turkmenistan	-	-	-	-	
-	Turks & Caicos Is.	-	-	-	-	
-	Tuvalu	-	-	-	-	
-	Uganda	-	-	-	-	
-	Vanuatu	-	-	-	-	
-	Vatican City	-	-	-	-	
-	Virgin Is.	-	-	-	-	
-	Wake I.	-	-	-	-	
-	Wallis & Futuna	-	-	-	-	
-	West Bank	-	-	-	-	
-	Western Sahara	-	-	-	-	
-	Zambia	-	-	-	-	
-	Zimbabwe	-	-	-	-	

Table S5 - Global OSA publications from 1900 to 2018 in relation to economic parameters.

Global OSA publication output from 1900 to 2018 in comparison with the country-specific economic parameters, sorted by number of OSA publications in descending order (empty cells: no data available from respective source).

- Gross Domestic Product 2018 based on Purchasing Power Parity (PPP), Billions, Current Int. Prices (source International Monetary Fund, accessed 02/18/2019) [1].
- Research & Development (R&D) Expenditure 2016 (or latest year available) based on PPP, Current Int. Prizes (*1000) (source UNESCO, accessed 02/18/2019) [2].
- Ratio R1: Number of OSA Publications / Total GDP.
- Ratio R2: Number of OSA Publications / Total R&D Expenditure.

	Country	# of OSA Publications 1900 to 2018	GDP 2018	R&D expenditure 2016 or latest year available	R1: # of OSA Publications/GDP	Ranking regarding R1	R2: # of OSA Publications/R&D	Ranking regarding R2
1	United States	11,611	20,513	511,089,000	5.66E-01	15	2.27E-05	41
2	Canada	1,947	1,853	25,725,958	1.05E+00	7	7.57E-05	4
3	China	1,758	25,797	455,323,855	6.81E-02	63	3.86E-06	77
4	Australia	1,534	1,319	21,318,581	1.16E+00	4	7.20E-05	7
5	Germany	1,456	4,379	118,790,567	3.32E-01	30	1.23E-05	60
6	Japan	1,413	5,632	165,739,961	2.51E-01	41	8.53E-06	66
7	United Kingdom	1,375	3,034	47,798,459	4.53E-01	26	2.88E-05	33
8	France	1,374	2,969	62,351,298	4.63E-01	23	2.20E-05	42
9	Italy	1,329	2,398	29,882,917	5.54E-01	16	4.45E-05	20
10	Turkey	1,093	2,314	16,604,485	4.72E-01	21	6.58E-05	8
11	Brazil	1,015	3,371	41,104,129	3.01E-01	33	2.47E-05	39
12	Spain	1,011	1,868	20,075,670	5.41E-01	18	5.04E-05	14
13	South Korea	654	2,140	77,656,294	3.06E-01	32	8.42E-06	67
14	Taiwan	567	1,250	33,700,000	4.54E-01	25	1.68E-05	51
15	Sweden	539	543	15,958,208	9.93E-01	8	3.38E-05	25
16	Belgium	492	550	13,130,902	8.95E-01	10	3.75E-05	23
17	Greece	466	313	2,870,613	1.49E+00	2	1.62E-04	3
18	Switzerland	450	551	17,688,255	8.16E-01	11	2.54E-05	37
18	Netherlands	450	972	17,751,156	4.63E-01	24	2.54E-05	38
20	Israel	448	336	13,728,500	1.33E+00	3	3.26E-05	26
21	Poland	321	1,202	10,229,317	2.67E-01	38	3.14E-05	30
22	Finland	289	257	6,549,458	1.12E+00	6	4.41E-05	21
23	Portugal	223	329	4,013,883	6.78E-01	12	5.56E-05	11
24	India	219	10,401	50,118,735	2.11E-02	92	4.37E-06	74
25	Ireland	191	379	4,010,820	5.05E-01	20	4.76E-05	19
26	Singapore	183	556	10,104,351	3.29E-01	31	1.81E-05	49
27	Norway	179	398	6,315,245	4.49E-01	27	2.83E-05	34

28	Denmark	164	300	8,197,929	5.46E-01	17	2.00E-05	46
29	Russia	147	4,180	37,265,830	3.52E-02	75	3.94E-06	76
30	Thailand	145	1,323	6,911,887	1.10E-01	54	2.10E-05	44
31	Austria	137	464	13,675,331	2.95E-01	34	1.00E-05	62
32	Saudi Arabia	128	1,857	12,513,565	6.89E-02	62	1.02E-05	61
33	Iran	119	1,653	3,317,226	7.20E-02	59	3.59E-05	24
34	New Zealand	118	199	2,227,866	5.92E-01	14	5.30E-05	12
35	Chile	114	481	1,574,241	2.37E-01	43	7.24E-05	6
36	Egypt	113	1,297	7,562,293	8.71E-02	57	1.49E-05	57
37	Romania	112	514	2,246,981	2.18E-01	47	4.98E-05	16
38	Czech Republic	108	396	6,228,688	2.72E-01	36	1.73E-05	50
39	Argentina	85	919	5,567,084	9.25E-02	56	1.53E-05	55
40	Iceland	80	19	361,124	4.15E+00	1	2.22E-04	2
41	Hungary	78	308	3,196,716	2.53E-01	40	2.44E-05	40
42	Mexico	77	2,575	11,410,458	2.99E-02	83	6.75E-06	70
43	Malaysia	56	1,000	10,659,652	5.60E-02	66	5.25E-06	72
44	Croatia	55	107	847,436	5.12E-01	19	6.49E-05	9
45	Slovakia	42	191	1,316,307	2.20E-01	46	3.19E-05	27
46	Tunisia	39	144	794,749	2.70E-01	37	4.91E-05	18
47	Serbia	38	113	925,509	3.38E-01	29	4.11E-05	22
48	Colombia	37	749	1,867,997	4.94E-02	71	1.98E-05	47
49	Bulgaria	26	163	1,257,758	1.60E-01	51	2.07E-05	45
50	Peru	25	458	496,863	5.45E-02	68	5.03E-05	15
51	Lebanon	24	91		2.63E-01	39		-
52	Lithuania	23	97	729,813	2.37E-01	42	3.15E-05	29
52	South Africa	23	791	5,823,289	2.91E-02	84	3.95E-06	75
52	United Arab Emirates	23	733	6,634,105	3.14E-02	80	3.47E-06	78
55	Kyrgyzstan	22	24	25,023	9.03E-01	9	8.79E-04	1
55	Pakistan	22	1,148	2,334,545	1.92E-02	93	9.42E-06	63
57	Jordan	21	93	284,599	2.25E-01	45	7.38E-05	5
57	Qatar	21	357	1,288,404	5.89E-02	65	1.63E-05	53
57	Slovenia	21	76	1,381,589	2.76E-01	35	1.52E-05	56
60	Estonia	15	44	499,629	3.39E-01	28	3.00E-05	31
61	Oman	14	198	443,294	7.06E-02	60	3.16E-05	28
62	Philippines	13	956	886,535	1.36E-02	99	1.47E-05	58
62	Ukraine	13	392	1,710,701	3.32E-02	77	7.60E-06	68
64	Nigeria	11	1,169		9.41E-03	104		-
65	Indonesia	10	3,496	2,130,257	2.86E-03	111	4.69E-06	73

66	Kuwait	8	303	1,205,227	2.64E-02	86	6.64E-06	71
67	Cyprus	7	34	141,224	2.07E-01	48	4.96E-05	17
67	Ecuador	7	200	808,410	3.50E-02	76	8.66E-06	65
67	Vietnam	7	708	2,446,375	9.89E-03	103	2.86E-06	81
67	Morocco	7	315		2.22E-02	90		-
71	Latvia	6	57	225,164	1.05E-01	55	2.66E-05	36
72	Iraq	5	675	234,789	7.41E-03	106	2.13E-05	43
72	Uruguay	5	82	264,626	6.13E-02	64	1.89E-05	48
72	Bolivia	5	89		5.60E-02	67		-
72	Cameroon	5	95		5.26E-02	69		-
72	Sri Lanka	5	293		1.71E-02	94		-
77	Moldova	4	25	62,839	1.59E-01	52	6.37E-05	10
77	Myanmar	4	359		1.11E-02	101		-
77	Guadeloupe	4				-		-
80	Malta	3	21	101,841	1.44E-01	53	2.95E-05	32
80	Georgia	3	43	112,235	6.97E-02	61	2.67E-05	35
80	Venezuela	3	320	1,103,990	9.37E-03	105	2.72E-06	82
80	Kenya	3	177		1.69E-02	95		-
80	Belarus	3	191		1.57E-02	96		-
80	Bangladesh	3	758		3.96E-03	108		-
80	Liechtenstein	3				-		-
87	Montenegro	2	12	37,965	1.70E-01	50	5.27E-05	13
87	Tanzania	2	176	623,754	1.14E-02	100	3.21E-06	79
87	Kazakhstan	2	508	639,217	3.94E-03	109	3.13E-06	80
87	Luxembourg	2	66	750,806	3.03E-02	82	2.66E-06	83
87	Grenada	2	2		1.15E+00	5		-
87	Albania	2	38		5.22E-02	70		-
87	Bahrain	2	75		2.66E-02	85		-
87	Nepal	2	86		2.32E-02	89		-
87	Algeria	2	661		3.03E-03	110		-
87	French Polynesia	2				-		-
97	Armenia	1	31	60,173	3.25E-02	79	1.66E-05	52
97	Burkina Faso	1	39	65,026	2.58E-02	87	1.54E-05	54
97	Paraguay	1	95	78,576	1.05E-02	102	1.27E-05	59
97	Mozambique	1	39	112,790	2.55E-02	88	8.87E-06	64
97	Macedonia	1	32	134,305	3.10E-02	81	7.45E-06	69
97	Uzbekistan	1	240	459,045	4.17E-03	107	2.18E-06	84
97	Cuba	1		582,720		-	1.72E-06	85

97	St. Kitts & Nevis	1	2		6.14E-01	13		-
97	San Marino	1	2		4.67E-01	22		-
97	Aruba	1	4		2.33E-01	44		-
97	Barbados	1	5		1.88E-01	49		-
97	Swaziland	1	12		8.32E-02	58		-
97	Malawi	1	24		4.22E-02	72		-
97	Jamaica	1	27		3.71E-02	73		-
97	Benin	1	28		3.63E-02	74		-
97	Congo	1	31		3.26E-02	78		-
97	Bosnia & Herzegovina	1	47		2.11E-02	91		-
97	Libya	1	70		1.42E-02	97		-
97	Yemen	1	73		1.36E-02	98		-
97	Andorra	1				-		-
97	Martinique	1				-		-
97	Netherlands Antilles	1				-		-
97	New Caledonia	1				-		-
97	Reunion	1				-		-
97	Syria	1				-		-
-	Azerbaijan	-	178	348,358	0.00E+00	-	0.00E+00	-
-	Cambodia	-	70	64,421	0.00E+00	-	0.00E+00	-
-	Cape Verde	-	4	2,211	0.00E+00	-	0.00E+00	-
-	Chad	-	30	91,046	0.00E+00	-	0.00E+00	-
-	Congo, DRC	-	73	10,234	0.00E+00	-	0.00E+00	-
-	Costa Rica	-	89	411,998	0.00E+00	-	0.00E+00	-
-	El Salvador	-	54	68,205	0.00E+00	-	0.00E+00	-
-	Ethiopia	-	221	787,274	0.00E+00	-	0.00E+00	-
-	Guatemala	-	145	37,785	0.00E+00	-	0.00E+00	-
-	Honduras	-	49	6,209	0.00E+00	-	0.00E+00	-
-	Madagascar	-	43	5,626	0.00E+00	-	0.00E+00	-
-	Mauritius	-	30	38,856	0.00E+00	-	0.00E+00	-
-	Mongolia	-	43	68,167	0.00E+00	-	0.00E+00	-
-	Namibia	-	28	81,658	0.00E+00	-	0.00E+00	-
-	Nicaragua	-	36	34,604	0.00E+00	-	0.00E+00	-
-	Panama	-	111	47,761	0.00E+00	-	0.00E+00	-
-	Papua New Guinea	-	31	10,990	0.00E+00	-	0.00E+00	-

-	Puerto Rico	-	130	548,271	0.00E+00	-	0.00E+00	-
-	Senegal	-	60	275,580	0.00E+00	-	0.00E+00	-
-	Seychelles	-	3	5,961	0.00E+00	-	0.00E+00	-
-	Tajikistan	-	31	27,860	0.00E+00	-	0.00E+00	-
-	The Gambia	-	6	3,544	0.00E+00	-	0.00E+00	-
-	Togo	-	14	27,210	0.00E+00	-	0.00E+00	-
-	Trinidad & Tobago	-	44	41,642	0.00E+00	-	0.00E+00	-
-	Uganda	-	97	114,150	0.00E+00	-	0.00E+00	-
-	Afghanistan	-	73		0.00E+00	-		-
-	Angola	-	198		0.00E+00	-		-
-	Antigua & Barbuda	-	3		0.00E+00	-		-
-	Belize	-	3		0.00E+00	-		-
-	Bhutan	-	8		0.00E+00	-		-
-	Botswana	-	42		0.00E+00	-		-
-	Brunei	-	35		0.00E+00	-		-
-	Burundi	-	8		0.00E+00	-		-
-	Comoros	-	1		0.00E+00	-		-
-	Cote dIvory	-	107		0.00E+00	-		-
-	Djibouti	-	4		0.00E+00	-		-
-	Dominica	-	1		0.00E+00	-		-
-	Dominican Republic	-	188		0.00E+00	-		-
-	Equatorial Guinea	-	30		0.00E+00	-		-
-	Eritrea	-	10		0.00E+00	-		-
-	Fiji	-	9		0.00E+00	-		-
-	Gabon	-	38		0.00E+00	-		-
-	Ghana	-	146		0.00E+00	-		-
-	Guinea	-	30		0.00E+00	-		-
-	Guinea-Bissau	-	3		0.00E+00	-		-
-	Guyana	-	7		0.00E+00	-		-
-	Haiti	-	21		0.00E+00	-		-
-	Kiribati	-	0		0.00E+00	-		-
-	Laos	-	54		0.00E+00	-		-
-	Lesotho	-	7		0.00E+00	-		-
-	Liberia	-	6		0.00E+00	-		-
-	Maldives	-	7		0.00E+00	-		-
-	Mali	-	44		0.00E+00	-		-

-	Marshall Is.	-	0		0.00E+00	-			-
-	Mauritania	-	18		0.00E+00	-			-
-	Micronesia	-	0		0.00E+00	-			-
-	Nauru	-	0		0.00E+00	-			-
-	Niger	-	24		0.00E+00	-			-
-	Palau	-	0		0.00E+00	-			-
-	Rwanda	-	27		0.00E+00	-			-
-	Samoa	-	1		0.00E+00	-			-
-	Sao Tome & Principe	-	1		0.00E+00	-			-
-	Sierra Leone	-	12		0.00E+00	-			-
-	Solomon Is.	-	1		0.00E+00	-			-
-	Somalia	-	22		0.00E+00	-			-
-	St. Lucia	-	3		0.00E+00	-			-
-	St. Vincent & the Grenadines	-	1		0.00E+00	-			-
-	Sudan	-	177		0.00E+00	-			-
-	Suriname	-	9		0.00E+00	-			-
-	The Bahamas	-	13		0.00E+00	-			-
-	Tonga	-	1		0.00E+00	-			-
-	Turkmenistan	-	113		0.00E+00	-			-
-	Tuvalu	-	0		0.00E+00	-			-
-	Vanuatu	-	1		0.00E+00	-			-
-	Zambia	-	73		0.00E+00	-			-
-	Zimbabwe	-	36		0.00E+00	-			-
-	American Samoa	-				-			-
-	Anguilla	-				-			-
-	Antarctica	-				-			-
-	Baker I.	-				-			-
-	Bermuda	-				-			-
-	Bouvet I.	-				-			-
-	British Indian Ocean Territory	-				-			-
-	British Virgin Is.	-				-			-
-	Cayman Is.	-				-			-

-	Central African Republic	-				-		-
-	Christmas I.	-				-		-
-	Cocos Is.	-				-		-
-	Cook Is.	-				-		-
-	East Timor	-				-		-
-	Falkland Is.	-				-		-
-	Faroe Is.	-				-		-
-	French Guiana	-				-		-
-	French Southern & Antarctic Lands	-				-		-
-	Gaza Strip	-				-		-
-	Gibraltar	-				-		-
-	Glorioso Is.	-				-		-
-	Greenland	-				-		-
-	Guam	-				-		-
-	Guernsey	-				-		-
-	Heard I. & McDonald Is.	-				-		-
-	Howland I.	-				-		-
-	Isle of Man	-				-		-
-	Jan Mayen	-				-		-
-	Jarvis I.	-				-		-
-	Jersey	-				-		-
-	Johnston Atoll	-				-		-
-	Juan De Nova I.	-				-		-
-	Mayotte	-				-		-
-	Midway Is.	-				-		-
-	Monaco	-				-		-
-	Montserrat	-				-		-
-	Niue	-				-		-
-	Norfolk I.	-				-		-
-	North Korea	-				-		-
-	Northern Mariana Is.	-				-		-
-	Paracel Is.	-				-		-

-	Pitcairn Is.	-				-		-
-	South Georgia & the South Sandwich Is.	-				-		-
-	Spratly Is.	-				-		-
-	St. Helena	-				-		-
-	St. Pierre & Miquelon	-				-		-
-	Svalbard	-				-		-
-	Tokelau	-				-		-
-	Turks & Caicos Is.	-				-		-
-	Vatican City	-				-		-
-	Virgin Is.	-				-		-
-	Wake I.	-				-		-
-	Wallis & Futuna	-				-		-
-	West Bank	-				-		-
-	Western Sahara	-				-		-

Table S6 - Percentage and weighted mean age of male population age 40 to ≤ 69 in 2013.

Percentage and weighted mean age of the countries' male population age 40 to ≤ 69 in 2013 in alphabetical order of countries (empty cells: no data available from respective source). Source for age-group specific percentage of male population in 2013 was The World Bank; accessed 02/2017 [3].

Country	% of male population age 40 to 44	% of male population age 45 to 49	% of male population age 50 to 54	% of male population age 55 to 59	% of male population age 60 to 64	% of male population age 65 to 69	Weighted mean age male population, ages 40 to ≤ 69
Afghanistan	3.98	3.15	2.48	1.92	1.45	1.02	50.84
Albania	6.00	6.58	7.14	6.29	4.78	3.84	53.27
Algeria	5.98	5.15	4.34	3.63	2.82	1.84	51.51
American Samoa							
Andorra							
Angola	3.83	3.04	2.45	1.96	1.42	0.84	50.75
Anguilla							
Antarctica							
Antigua & Barbuda	8.16	8.04	5.74	4.63	3.07	1.84	50.72
Argentina	6.03	5.42	5.02	4.55	3.92	3.13	52.77
Armenia	5.39	5.94	7.11	6.23	4.09	2.46	52.81
Aruba	7.33	8.27	8.60	7.08	5.61	4.12	52.94
Australia	6.96	6.69	6.65	5.94	5.39	4.59	53.37
Austria	7.73	8.73	8.07	6.47	5.27	5.10	52.98
Azerbaijan	6.46	6.86	6.60	4.69	2.75	1.43	51.08
Bahrain	9.31	7.00	5.20	3.59	1.63	0.86	49.06
Baker I.							
Bangladesh	6.07	5.44	4.22	2.82	2.04	1.82	50.84
Barbados	7.22	7.15	7.22	6.40	5.26	4.06	53.01
Belarus	7.04	7.29	7.99	7.01	5.14	3.35	52.79
Belgium	7.16	7.51	7.42	6.62	5.86	4.80	53.38
Belize	5.70	4.88	3.98	2.90	2.10	1.45	50.85
Benin	4.24	3.46	2.72	2.11	1.55	1.11	50.88
Bermuda							
Bhutan	5.95	5.02	3.69	3.01	2.11	1.73	50.96
Bolivia	5.17	4.29	3.60	3.10	2.55	1.99	51.88
Bosnia & Herzegovina	7.07	7.36	8.02	7.19	5.85	3.83	53.13

Botswana	5.29	3.95	3.15	2.62	1.93	1.23	50.80
Bouvet I.							
Brazil	6.74	6.25	5.48	4.43	3.46	2.47	51.83
British Indian Ocean Territory							
British Virgin Is.							
Brunei	7.67	6.57	5.40	4.02	2.45	1.37	50.38
Bulgaria	7.60	7.04	7.06	6.97	6.77	5.72	53.87
Burkina Faso	3.82	2.99	2.24	1.63	1.17	0.86	50.39
Burundi	3.46	3.23	2.88	2.41	1.58	0.92	51.38
Cambodia	5.54	4.78	3.73	2.47	2.11	1.36	50.73
Cameroon	4.07	3.17	2.47	1.95	1.55	1.17	51.04
Canada	6.71	7.42	8.00	7.06	6.02	4.86	53.60
Cape Verde	4.98	4.55	3.81	2.61	1.63	0.75	50.26
Cayman Is.							
Central African Republic	4.10	3.29	2.68	2.17	1.70	1.32	51.36
Chad	3.49	2.70	2.20	1.80	1.37	0.96	51.10
Chile	7.22	7.03	6.26	5.39	4.26	3.09	52.26
China	8.95	8.45	6.61	5.75	5.12	3.21	51.91
Christmas I.							
Cocos Is.							
Colombia	6.60	6.43	5.38	4.33	3.35	2.29	51.69
Comoros	4.52	3.76	3.06	2.36	1.63	1.08	50.80
Congo	4.95	3.82	2.88	2.16	1.64	1.22	50.62
Congo, DRC	4.01	3.27	2.61	2.05	1.58	1.18	51.14
Cook Is.							
Costa Rica	6.40	6.54	5.93	4.65	3.62	2.91	52.21
Cote dIvory	4.34	3.68	3.13	2.58	1.98	1.42	51.55
Croatia	6.92	6.91	7.45	7.37	6.69	4.83	53.80
Cuba	9.25	9.33	7.75	5.48	5.08	3.93	51.95
Cyprus	6.42	6.28	6.14	5.52	4.78	3.88	53.15
Czech Republic	7.92	6.65	6.50	6.65	6.82	5.62	53.82
Denmark	7.17	7.58	6.93	6.11	6.28	6.22	53.91
Djibouti	5.27	4.65	3.82	2.63	1.91	1.59	51.00
Dominica							
Dominican Republic	5.80	5.21	4.55	3.72	2.82	2.04	51.72

East Timor							
Ecuador	6.03	5.22	4.41	3.72	2.88	2.05	51.66
Egypt	5.37	4.81	4.19	3.33	2.58	1.86	51.67
El Salvador	5.22	4.60	3.98	3.36	2.79	2.25	52.15
Equatorial Guinea	5.13	3.64	2.48	2.03	1.54	1.07	50.24
Eritrea							
Estonia	7.32	6.85	7.13	6.56	5.59	4.32	53.22
Ethiopia	4.13	3.09	2.44	2.05	1.67	1.30	51.30
Falkland Is.							
Faroe Is.							
Fiji	5.93	5.83	5.54	4.37	3.13	2.29	51.97
Finland	6.18	6.95	7.07	6.87	7.18	6.25	54.55
France	7.07	6.98	6.71	6.25	6.20	5.16	53.70
French Guiana							
French Polynesia	7.47	7.34	5.99	4.90	3.57	2.53	51.58
French Southern & Antarctic Lands							
Gabon	5.55	4.20	3.04	2.29	1.84	1.48	50.68
Gaza Strip							
Georgia	6.61	6.70	7.07	6.12	4.77	3.40	52.85
Germany	7.30	8.94	8.43	7.08	5.85	5.16	53.25
Ghana	4.76	3.90	3.05	2.31	1.77	1.36	50.99
Gibraltar							
Glorioso Is.							
Greece	8.00	7.67	7.00	6.36	5.48	4.78	53.02
Greenland							
Grenada	5.14	4.78	4.94	3.56	2.73	1.82	51.87
Guadeloupe							
Guam	6.78	6.90	6.24	4.90	4.08	2.94	52.22
Guatemala	4.14	3.25	2.67	2.33	1.87	1.32	51.52
Guernsey							
Guinea	4.16	3.49	2.97	2.47	1.86	1.23	51.41
Guinea-Bissau	4.20	3.26	2.67	2.25	1.78	1.14	51.21
Guyana	6.51	5.80	4.97	3.90	2.72	1.83	51.22
Haiti	4.56	4.17	3.51	2.84	2.15	1.53	51.58
Heard I. & McDonald Is.							

Honduras	5.00	4.03	3.17	2.56	1.94	1.35	51.01
Howland I.							
Hungary	7.98	6.56	6.33	7.39	6.67	4.67	53.54
Iceland	6.56	6.48	6.64	6.16	5.26	4.13	53.35
India	6.05	5.31	4.61	3.88	2.96	1.96	51.65
Indonesia	7.01	6.07	5.05	4.03	2.77	1.80	51.05
Iran	6.54	5.76	4.60	3.72	2.71	1.66	51.05
Iraq	4.88	3.56	2.30	1.99	1.70	1.06	50.46
Ireland	7.57	6.66	6.27	5.47	5.15	4.18	52.92
Isle of Man							
Israel	6.04	5.16	4.72	4.58	4.49	3.34	53.12
Italy	8.29	8.29	7.63	6.58	6.23	5.65	53.30
Jamaica	6.29	5.97	5.19	4.20	3.46	2.70	52.12
Jan Mayen							
Japan	7.91	6.73	6.28	6.41	7.38	7.10	54.38
Jarvis I.							
Jersey							
Johnston Atoll							
Jordan	5.94	4.65	3.28	2.23	1.67	1.35	50.19
Juan De Nova I.							
Kazakhstan	6.41	6.14	5.96	4.42	3.04	1.69	51.39
Kenya	4.24	3.07	2.32	1.80	1.34	0.89	50.39
Kiribati	4.98	5.11	4.01	2.80	2.00	1.28	50.90
Kuwait	10.86	8.06	5.18	3.70	1.88	0.86	48.76
Kyrgyzstan	5.61	5.45	4.80	3.48	2.23	1.04	50.76
Laos	4.92	4.11	3.37	2.66	1.97	1.32	51.08
Latvia	7.30	7.19	7.52	6.67	5.26	4.30	53.08
Lebanon	6.37	6.92	6.06	4.18	3.29	2.95	51.99
Lesotho	3.67	2.51	2.08	2.07	1.94	1.51	52.23
Liberia	4.44	3.55	2.83	2.20	1.63	1.21	50.94
Libya	7.53	5.44	3.48	2.51	1.90	1.41	49.77
Liechtenstein							
Lithuania	7.30	7.73	7.96	6.61	5.02	4.12	52.86
Luxembourg	8.22	8.53	7.47	6.20	5.10	4.02	52.44
Macedonia	7.23	7.16	6.82	6.55	5.43	3.75	52.96
Madagascar	4.45	3.57	2.85	2.26	1.62	1.01	50.76
Malawi	3.71	2.60	1.81	1.35	1.21	1.10	50.75

Malaysia	6.07	5.46	4.94	3.99	3.08	2.15	51.81
Maldives	6.04	4.85	3.83	2.59	1.50	1.18	50.05
Mali	3.79	2.74	2.05	1.68	1.32	0.94	50.74
Malta	6.34	6.17	7.09	7.09	6.99	6.14	54.59
Marshall Is.							
Martinique							
Mauritania	4.80	3.84	3.05	2.33	1.64	1.10	50.65
Mauritius	6.89	7.45	7.32	6.03	4.88	3.25	52.60
Mayotte							
Mexico	6.68	5.08	4.21	3.55	2.78	2.00	51.32
Micronesia	3.00	3.54	3.69	3.46	2.91	1.51	53.18
Midway Is.							
Moldova	6.31	6.50	7.26	6.62	4.93	2.53	52.72
Monaco							
Mongolia	6.82	5.83	4.66	3.01	1.94	1.31	50.16
Montserrat							
Morocco	5.70	5.57	5.36	4.48	3.18	2.09	52.02
Mozambique	3.59	2.77	2.38	1.98	1.55	1.13	51.44
Myanmar	6.57	5.68	4.79	4.05	2.99	1.84	51.37
Namibia	4.70	3.69	2.86	2.11	1.59	1.19	50.69
Nauru							
Nepal	5.09	4.47	3.83	3.28	2.84	2.05	52.11
Netherlands	7.32	7.89	7.52	6.72	6.39	5.63	53.67
Netherlands Antilles							
New Caledonia	7.78	7.08	6.30	4.86	3.81	3.20	51.91
New Zealand	6.68	6.87	6.84	5.95	5.35	4.58	53.40
Nicaragua	5.17	4.16	3.65	3.13	2.10	1.37	51.22
Niger	3.49	3.31	3.30	2.37	1.63	1.08	51.52
Nigeria	4.28	3.41	2.78	2.17	1.66	1.23	51.10
Niue							
Norfolk I.							
Northern Mariana Is.							
North Korea	9.49	7.90	6.29	5.16	2.82	3.29	51.11
Norway	7.61	7.32	6.62	6.09	5.78	5.16	53.37
Oman	7.56	4.89	3.55	2.40	1.23	0.65	48.75
Pakistan	4.97	4.33	3.61	2.73	2.04	1.73	51.41

Palau							
Panama	6.67	5.83	4.89	3.90	3.03	2.32	51.58
Papua New Guinea	5.29	4.33	3.51	2.76	2.00	1.30	50.89
Paracel Is.							
Paraguay	5.04	4.63	4.05	3.51	2.84	2.02	52.12
Peru	6.17	5.41	4.47	3.54	2.86	2.16	51.59
Philippines	5.83	5.16	4.28	3.38	2.44	1.61	51.17
Pitcairn Is.							
Poland	6.77	6.25	7.08	7.61	6.31	4.21	53.71
Portugal	7.80	7.31	7.13	6.49	6.05	5.34	53.46
Puerto Rico	6.55	6.32	5.94	5.29	4.73	4.18	53.19
Qatar	10.62	7.16	4.55	2.52	1.10	0.34	47.69
Reunion							
Romania	8.27	7.17	5.88	7.13	6.14	4.26	53.09
Russia	6.77	6.72	7.89	7.07	5.34	2.99	52.88
Rwanda	3.75	3.55	3.38	2.26	1.58	0.96	51.10
Samoa	5.58	5.01	4.11	3.37	2.34	1.60	51.25
San Marino							
Sao Tome & Principe	3.93	3.31	2.38	1.61	1.04	0.68	49.90
Saudi Arabia	9.29	7.00	5.16	3.34	2.03	1.09	49.33
Senegal	3.89	2.99	2.35	1.88	1.47	1.07	50.99
Serbia	6.75	6.39	6.46	7.09	7.09	4.81	54.05
Seychelles	8.07	7.43	6.74	5.29	3.37	2.31	51.30
Sierra Leone	4.31	3.34	2.62	2.03	1.61	1.22	50.99
Singapore	8.21	8.27	8.46	7.39	5.79	3.88	52.70
Slovakia	7.33	6.75	7.15	7.02	5.91	4.00	53.24
Slovenia	7.52	7.68	7.62	7.65	6.60	4.62	53.44
Solomon Is.	5.12	3.80	3.07	2.34	1.74	1.37	50.83
Somalia	3.86	3.16	2.61	2.16	1.68	1.18	51.38
South Africa	5.84	4.78	4.03	3.32	2.50	1.67	51.30
South Georgia & the South Sandwich Is.							
South Korea	8.95	8.62	8.19	6.85	4.86	3.87	52.20
Spain	8.80	8.15	7.49	6.30	5.29	4.68	52.63
Spratly Is.							
Sri Lanka	6.66	6.43	5.93	5.16	4.36	3.37	52.67

St. Helena							
St. Kitts & Nevis							
St. Lucia	7.17	7.08	6.03	4.55	3.53	2.70	51.72
St. Pierre & Miquelon							
St. Vincent & the Grenadines	6.48	6.60	6.33	4.58	3.16	2.17	51.64
Sudan	4.46	3.73	2.94	2.33	1.81	1.32	51.17
Suriname	6.96	6.49	5.74	3.91	2.85	2.07	51.18
Svalbard							
Swaziland	3.56	2.78	2.16	1.76	1.42	1.08	51.19
Sweden	7.03	7.02	6.40	5.92	6.04	6.04	53.95
Switzerland	7.73	8.47	7.78	6.43	5.65	5.19	53.13
Syria	4.70	4.08	3.43	2.67	1.96	1.29	51.17
Taiwan							
Tajikistan	4.98	4.40	3.74	2.84	1.83	1.04	50.74
Tanzania	4.06	3.10	2.40	1.85	1.42	1.12	50.86
Thailand	8.39	8.15	7.33	6.09	4.44	3.16	51.94
The Bahamas	7.45	6.96	6.45	4.93	3.74	2.66	51.77
The Gambia	3.65	3.10	2.38	1.92	1.42	1.03	51.05
Togo	4.55	3.52	2.74	2.13	1.62	1.17	50.82
Tokelau							
Tonga	5.30	4.10	3.66	3.10	1.90	1.63	51.26
Trinidad & Tobago	6.59	6.70	6.97	5.66	4.46	3.22	52.65
Tunisia	6.50	6.45	5.86	4.88	3.54	2.20	51.85
Turkey	6.68	5.79	4.94	4.06	3.18	2.40	51.72
Turkmenistan	5.92	5.30	4.46	3.46	2.18	1.21	50.74
Turks & Caicos Is.							
Tuvalu							
Uganda	3.56	2.51	1.74	1.31	1.02	0.76	50.17
Ukraine	7.03	6.89	7.33	6.70	5.49	3.68	53.05
United Arab Emirates	12.83	7.93	4.79	2.12	0.82	0.48	47.10
United Kingdom	6.85	7.31	6.87	5.88	5.63	5.30	53.59
United States	6.51	6.95	7.15	6.58	5.58	4.32	53.44
Uruguay	6.45	5.92	5.85	5.23	4.44	3.71	53.01
Uzbekistan	5.76	5.17	4.77	3.66	2.23	1.12	50.85

Vanuatu	4.93	4.33	3.59	2.69	2.13	1.61	51.37
Vatican City							
Venezuela	6.14	5.93	5.07	3.79	2.86	2.10	51.53
Vietnam	7.10	6.41	5.62	4.39	2.75	1.53	50.90
Virgin Is.	6.35	6.99	7.35	7.05	6.46	6.32	54.37
Wake I.							
Wallis & Futuna							
West Bank	4.42	3.64	2.73	1.88	1.52	1.22	50.73
Western Sahara							
Yemen	3.57	2.83	2.43	2.03	1.60	1.17	51.54
Zambia	3.84	2.67	1.90	1.37	1.07	0.86	50.18
Zimbabwe	3.71	2.55	1.90	1.50	1.14	0.85	50.43

Table S7 - Country-specific mean Body Mass Index (BMI) and weighted mean BMI of male population age 40 to ≤ 69 in 2013.

Country-specific mean Body Mass Index (BMI) and weighted mean BMI of male population age 40 to ≤ 69 in 2013 in alphabetical order of countries (empty cells: no data available from respective source). Source of the age- and gender-specific BMI in 2013 was the NCD Risk Factor Collaboration, The Lancet, April 2016 [4].

Country	Mean BMI ages 40≤44	Mean BMI ages 45≤49	Mean BMI ages 50≤54	Mean BMI ages 55≤59	Mean BMI ages 60≤64	Mean BMI ages 65≤69	Weighted mean BMI all age groups
Afghanistan	24.48261935	24.76289558	24.887268	24.866472	24.712106	24.43711	24.69048876
Albania	26.90915027	27.47294744	27.873852	28.111808	28.188223	28.137248	27.74627383
Algeria	27.90239398	28.34383922	28.465271	28.204924	27.501296	26.304541	27.97593028
American Samoa	37.58679965	37.84936565	37.690014	37.068628	35.945569	34.356132	
Andorra	27.27159478	27.69638204	27.924893	27.949098	27.761885	27.377725	
Angola	24.65007867	24.96192727	25.119003	25.126793	24.991656	24.722792	24.91415835
Anguilla							
Antarctica							
Antigua & Barbuda	28.60633134	29.08964063	29.353049	29.381563	29.161138	28.708875	29.03994333
Argentina	28.17316113	28.93017322	29.515999	29.893656	30.026345	29.89103	29.28903226
Armenia	27.47396603	28.56799218	29.53094	30.292858	30.784747	30.967677	29.42220131
Aruba							
Australia	27.26581445	27.86765031	28.337625	28.637842	28.73128	28.603212	28.18652445
Austria	24.96003773	25.66983744	26.272015	26.72733	26.997442	27.05357	26.15984642
Azerbaijan	28.78721976	29.56726424	30.123593	30.424745	30.440226	30.175153	29.77313863
Bahrain	25.64467001	25.53881967	25.289176	24.922187	24.466576	23.988151	25.33575376
Baker I.							
Bangladesh	22.34912586	22.25285428	21.994371	21.634998	21.236857	20.845	21.94551775
Barbados	30.15820931	30.441648	30.521643	30.374163	29.97627	29.347332	30.20596608
Belarus	27.3938728	28.39956513	29.242538	29.887135	30.298653	30.470958	29.10776527
Belgium	25.80258021	26.51683091	27.154566	27.678277	28.051054	28.243658	27.14113986
Belize	31.93246207	32.18514942	32.148905	31.793198	31.088241	30.049663	31.79882841
Benin	25.23367219	25.42972807	25.407618	25.168806	24.715661	24.056857	25.16193205
Bermuda	30.4602099	31.04747556	31.384251	31.445449	31.206947	30.688879	
Bhutan	25.05374487	25.10104115	24.964691	24.659214	24.200061	23.607109	24.7941045
Bolivia	28.61695005	28.90117221	28.963394	28.800509	28.410379	27.820515	28.66176318
Bosnia & Herzegovina	25.70811162	26.57422494	27.287734	27.818618	28.137083	28.213503	27.18369283

Botswana	27.18447008	27.71005235	28.016235	28.102412	27.968893	27.637802	27.68943072
Bouvet I.							
Brazil	27.43030699	27.89166663	28.184322	28.31219	28.278954	28.084989	27.96706835
British Indian Ocean Territory							
British Virgin Is.							
Brunei	28.25572517	28.37181657	28.239286	27.879758	27.315753	26.593957	28.05863882
Bulgaria	26.57635738	27.39482205	28.012576	28.412103	28.576468	28.502032	27.87022169
Burkina Faso	22.50525617	22.4513343	22.26425	21.987144	21.66419	21.329034	22.22682728
Burundi	21.61381322	21.57772497	21.457425	21.272784	21.044487	20.772417	21.40206533
Cambodia	22.72810001	22.83558701	22.773237	22.565717	22.238498	21.804388	22.62764078
Cameroon	25.96114587	26.07159867	25.964677	25.648008	25.131686	24.471398	25.73348352
Canada	27.21061801	27.76565575	28.187595	28.454959	28.546467	28.446794	28.07838256
Cape Verde	25.95172945	26.30152641	26.482552	26.49137	26.325425	25.993934	26.26067164
Cayman Is.							
Central African Republic	23.7757377	24.0914879	24.302664	24.406116	24.399544	24.277714	24.1388962
Chad	22.28782194	22.39806148	22.378716	22.257959	22.064809	21.813801	22.26257911
Chile	28.9117409	29.53253089	29.973032	30.193016	30.152499	29.82997	29.69490807
China	23.95191155	24.35189662	24.592595	24.667421	24.571465	24.317239	24.37397533
Christmas I.							
Cocos Is.							
Colombia	27.41453516	27.9606537	28.310899	28.446694	28.350391	28.028296	28.0257247
Comoros	26.54288742	26.5347218	26.276404	25.80424	25.155418	24.379127	26.10468005
Congo	24.98993294	25.12070675	25.033356	24.753955	24.309729	23.737479	24.83826246
Congo, DRC	22.86184046	23.02696917	23.073521	23.025182	22.906475	22.73121	22.95325369
Cook Is.	35.18307992	35.34243491	35.151437	34.590155	33.6397	32.352675	#DIV/0!
Costa Rica	28.79831235	29.32337692	29.637559	29.716648	29.536867	29.095426	29.33793883
Cote dIvory	24.95818947	25.06472062	24.981801	24.722697	24.301542	23.735307	24.77255372
Croatia	27.38954532	28.28403893	28.992289	29.479971	29.713616	29.685124	28.8871234
Cuba	26.85114876	27.22106167	27.412524	27.428298	27.271345	26.946371	27.18130571
Cyprus	26.55307081	27.53097957	28.389289	29.088846	29.591434	29.881576	28.33525945
Czech Republic	26.6375746	27.69917678	28.649568	29.446736	30.049576	30.440306	28.71566788
Denmark	24.86634597	25.25027413	25.538029	25.739323	25.864623	25.926457	25.50584713
Djibouti	25.33187405	25.66996421	25.845427	25.859927	25.71603	25.424475	25.62408207
Dominica	30.09054772	30.49616498	30.665928	30.584242	30.236464	29.647837	

Dominican Republic	28.35500658	28.73738015	28.891027	28.812825	28.500429	27.976268	28.59419245
East Timor							
Ecuador	28.58606138	29.0406663	29.295363	29.326603	29.111768	28.65863	28.99402781
Egypt	32.6148582	33.17706053	33.440928	33.369881	32.930728	32.20279	33.00891996
El Salvador	29.41133634	29.72785677	29.795569	29.607725	29.158566	28.478057	29.44903509
Equatorial Guinea	26.40789249	26.6050834	26.636979	26.495234	26.172389	25.674168	26.42783565
Eritrea	21.48374355	21.65840358	21.694642	21.621979	21.470786	21.252685	
Estonia	26.27051803	27.18069548	27.977508	28.608248	29.020828	29.176663	27.90330342
Ethiopia	21.13481515	21.19894502	21.177163	21.105707	21.021621	20.938772	21.12101298
Falkland Is.							
Faroe Is.							
Fiji	30.03822533	30.4943095	30.697337	30.618761	30.230525	29.534142	30.34456634
Finland	25.98857902	26.76639738	27.435757	27.972987	28.35491	28.56581	27.52855674
France	24.6162043	25.31707864	25.924007	26.415684	26.771384	26.970235	25.93046651
French Guiana							NA
French Polynesia	30.67826937	30.68363572	30.479696	30.041068	29.343337	28.402338	30.21339459
French Southern & Antarctic Lands							NA
Gabon	27.61807652	27.89208212	27.907165	27.672817	27.199423	26.519022	27.60488779
Gaza Strip							NA
Georgia	28.34421393	29.32847088	30.102194	30.619863	30.836923	30.743433	29.8724082
Germany	26.01986459	26.85495147	27.611155	28.242363	28.703249	28.963193	27.59846702
Ghana	26.01689113	26.20531367	26.178947	25.954892	25.552544	25.034138	25.95429005
Gibraltar							NA
Glorioso Is.							NA
Greece	27.53370968	28.44498378	29.201406	29.755906	30.061607	30.082989	29.03149284
Greenland	27.32391847	27.49783442	27.531719	27.429089	27.194731	26.860773	#DIV/0!
Grenada	29.75894832	30.1424375	30.291086	30.185078	29.80556	29.171475	29.97834202
Guadeloupe							NA
Guam							NA
Guatemala	28.27025773	28.57001875	28.646404	28.493405	28.105501	27.494128	28.34518672
Guernsey							NA
Guinea	23.59745348	23.83958413	23.979508	24.035601	24.02711	23.968787	23.86430931
Guinea-Bissau	24.5079025	24.82822305	24.999443	25.02471	24.908068	24.656198	24.79549638
Guyana	28.75135796	29.25359113	29.504439	29.508864	29.272764	28.833958	29.1858415

Haiti	24.93702316	24.95949845	24.791866	24.480144	24.071231	23.614095	24.63881984
Heard I. & McDonald Is.							NA
Honduras	28.75243348	29.05286603	29.079518	28.823453	28.276359	27.453063	28.73895533
Howland I.							NA
Hungary	26.91915324	27.8544129	28.647394	29.244855	29.594024	29.656298	28.55776279
Iceland	25.85578221	26.39025707	26.818977	27.124016	27.288432	27.310438	26.74206685
India	22.57193418	22.71237704	22.661413	22.458785	22.145554	21.761029	22.48575334
Indonesia	24.75546372	24.64205595	24.287207	23.751921	23.098446	22.39664	24.15874076
Iran	28.38918314	28.83508079	29.000716	28.884176	28.48543	27.854718	28.65327437
Iraq	30.70733283	31.28115039	31.616979	31.674231	31.413402	30.84336	31.18558318
Ireland	27.62363497	28.1189574	28.467816	28.659536	28.684813	28.568863	28.29425973
Isle of Man							NA
Israel	27.45927857	28.25031837	28.915911	29.419091	29.723539	29.812873	28.79920732
Italy	25.23856603	26.03619606	26.719371	27.252903	27.602672	27.750306	26.64675552
Jamaica	31.00954054	31.23114344	31.143644	30.775429	30.156232	29.368521	30.78112045
Jan Mayen							NA
Japan	22.13964262	22.53208954	22.820164	23.000971	23.073039	23.034159	22.75370546
Jarvis I.							NA
Jersey							NA
Johnston Atoll							NA
Jordan	31.08728935	32.0853794	32.823458	33.232069	33.243371	32.856535	32.19081071
Juan De Nova I.							NA
Kazakhstan	27.26136227	28.13510269	28.825439	29.297145	29.515918	29.473173	28.50072362
Kenya	25.05348198	25.17445365	25.113285	24.891943	24.533272	24.063842	24.95384774
Kiribati	32.80674099	32.74950715	32.366317	31.666695	30.66162	29.425924	32.11937083
Kuwait	31.60715582	32.21061838	32.584587	32.689156	32.486418	32.013429	32.1287923
Kyrgyzstan	27.51317693	28.39400783	29.121788	29.663952	29.988865	30.092522	28.76066696
Laos	23.5456566	23.74848179	23.782149	23.661954	23.40402	23.018449	23.59812896
Latvia	26.7935773	27.71685854	28.497439	29.119972	29.570115	29.858524	28.43413772
Lebanon	28.1650811	29.10271703	29.854358	30.379891	30.640194	30.629775	29.55573341
Lesotho	28.09872148	28.36024254	28.393353	28.195628	27.765584	27.12748	28.05229015
Liberia	25.94371445	26.08690382	26.001893	25.706895	25.221024	24.573628	25.77487913
Libya	30.90448344	31.7427627	32.256042	32.418699	32.20592	31.638706	31.6487443
Liechtenstein							NA
Lithuania	26.44396655	27.38859861	28.213485	28.889316	29.387249	29.689309	28.13963218
Luxembourg	26.12398511	26.74394946	27.238545	27.60374	27.836437	27.949855	27.1069401
Macedonia	27.14212907	27.64830754	27.945834	28.034611	27.915459	27.61063	27.70818144
Madagascar	21.3889518	21.36599694	21.235988	21.052041	20.868396	20.723992	21.2114296

Malawi	23.69320803	23.65033769	23.458263	23.1557	22.782357	22.374051	23.36906691
Malaysia	27.12378595	27.37146429	27.374314	27.140433	26.678635	26.012097	27.08069911
Maldives	27.29255462	27.837944	28.128245	28.180875	28.0142	27.669883	27.77664182
Mali	23.8773634	23.85408061	23.631666	23.243809	22.725058	22.104487	23.49188921
Malta	27.40282692	28.28852204	28.996176	29.499912	29.774774	29.822715	28.98673597
Marshall Is.	32.40709439	32.70370704	32.659015	32.278213	31.567506	30.587695	NA
Martinique							NA
Mauritania	27.84641845	28.09870555	28.088127	27.829876	27.340067	26.658421	27.8184075
Mauritius	26.81171752	27.28331731	27.547607	27.601419	27.442522	27.088249	27.30416448
Mayotte							NA
Mexico	29.5019494	29.9129426	30.094378	30.041216	29.748856	29.237134	29.77573505
Micronesia	32.59992981	32.79275896	32.746897	32.431717	31.817631	30.930592	32.37050762
Midway Is.							NA
Moldova	28.18126836	29.16114268	29.95092	30.514631	30.817277	30.857614	29.77493179
Monaco							NA
Mongolia	27.45593335	27.96078876	28.237291	28.273079	28.056734	27.600932	27.89724424
Montserrat							NA
Morocco	27.63243609	27.97300983	28.064839	27.907143	27.500189	26.870312	27.76252617
Mozambique	23.80194993	23.95377125	23.96074	23.836986	23.597488	23.252786	23.79694572
Myanmar	23.94748006	24.14579325	24.111217	23.860709	23.411929	22.776131	23.86290814
Namibia	26.34779405	26.70052965	26.834306	26.766472	26.515125	26.109454	26.56819538
Nauru	35.44494386	35.23933819	34.644963	33.664213	32.300548	30.627781	NA
Nepal	22.85082812	22.89630702	22.771951	22.516177	22.168497	21.762544	22.60199338
Netherlands	25.40586673	25.97712567	26.477828	26.89532	27.217686	27.439907	26.50567747
Netherlands Antilles							NA
New Caledonia							NA
New Zealand	28.46805747	28.83331354	29.077654	29.189758	29.157581	28.960164	28.9344382
Nicaragua	29.26235132	29.75798883	29.972416	29.861082	29.378632	28.475533	29.55281561
Niger	23.030926	22.96449974	22.707878	22.298346	21.774007	21.160887	22.5644969
Nigeria	24.77653651	24.93593277	24.893936	24.67402	24.301122	23.815955	24.69121539
Niue	34.60372016	34.64156547	34.441813	33.980218	33.234688	32.274952	NA
Norfolk I.							NA
Northern Mariana Is.							NA
North Korea	24.46923712	24.76347722	24.907573	24.908493	24.77406	24.513287	24.7082084
Norway	26.53798771	27.15379589	27.656494	28.034887	28.277886	28.374685	27.58944714
Oman	27.75492456	27.83598743	27.642199	27.200442	26.53905	25.719823	27.54990795
Pakistan	25.67989092	25.82061499	25.706475	25.37366	24.860362	24.238187	25.45878388

Palau	30.91431711	31.19524262	31.25293	31.051668	30.556762	29.778783	#DIV/0!
Panama	28.74603345	29.05860647	29.135607	28.970838	28.558753	27.91747	28.82541074
Papua New Guinea	26.57819212	26.48694917	26.213271	25.751794	25.098058	24.259679	26.06062112
Paracel Is.							NA
Paraguay	27.68073883	28.1267178	28.368432	28.395893	28.199999	27.795272	28.09128256
Peru	28.00078683	28.33936822	28.459182	28.35116	28.007438	27.451876	28.16158194
Philippines	24.39447656	24.39766777	24.182556	23.798975	23.297508	22.724015	24.03045148
Pitcairn Is.							NA
Poland	26.33080604	27.29546641	28.137527	28.812275	29.27526	29.48797	28.15034228
Portugal	25.64920932	26.38885653	27.032739	27.554677	27.929432	28.146465	27.01421859
Puerto Rico	29.66192049	30.17730239	30.446997	30.454971	30.185855	29.655892	30.10325501
Qatar	31.35287416	31.93398868	32.257989	32.29037	31.99752	31.394084	31.78511754
Reunion							NA
Romania	27.54756535	28.34241671	28.926409	29.266855	29.331578	29.106008	28.67121838
Russia	27.54002149	28.51587382	29.279171	29.765837	29.913453	29.705651	29.03968226
Rwanda	23.05357774	22.83437214	22.49367	22.070391	21.604302	21.122302	22.47047576
Samoa	35.47353403	35.90484808	36.0537	35.875286	35.326351	34.451067	35.65142043
San Marino							NA
Sao Tome & Principe	26.44202118	26.8108717	26.980573	26.957364	26.748415	26.376964	26.72043205
Saudi Arabia	30.81899736	31.25184144	31.390112	31.228627	30.764214	30.061971	31.04853232
Senegal	24.79634802	25.21024611	25.431891	25.466081	25.318348	24.995831	25.16022914
Serbia	26.07544468	27.06246387	27.899122	28.52099	28.864412	28.882113	27.85567872
Seychelles	29.21838244	29.86566348	30.249947	30.361841	30.192886	29.770782	29.89199988
Sierra Leone	24.18310896	24.22423629	24.105982	23.855311	23.500046	23.065121	23.97230943
Singapore	23.72877267	24.15720457	24.438538	24.590354	24.630743	24.567676	24.30951362
Slovakia	25.82596066	26.78434072	27.631864	28.327616	28.831585	29.120995	27.60506397
Slovenia	27.47297873	27.94962447	28.214062	28.264181	28.098783	27.739788	27.97001092
Solomon Is.	28.12993039	28.23641084	28.086009	27.673925	26.996292	26.073601	27.80892994
Somalia	24.3067102	24.59984613	24.74527	24.751168	24.626621	24.381733	24.5564561
South Africa	30.56736951	31.11600423	31.410878	31.426249	31.138032	30.582425	31.03368126
South Georgia & the South Sandwich Is.							NA
South Korea	23.37810719	23.8557788	24.232949	24.49287	24.619497	24.589311	24.09111222
Spain	25.22056218	26.14526628	26.997376	27.71892	28.252948	28.55812	26.89687908
Spratly Is.							NA
Sri Lanka	24.49942899	24.62764594	24.533861	24.267256	23.877906	23.416467	24.29481226
St. Helena							NA

St. Kitts & Nevis	31.57395658	32.07070652	32.341648	32.340626	32.0225	31.393738	NA
St. Lucia	31.2023853	31.55742332	31.675821	31.550257	31.174924	30.602955	31.37103909
St. Pierre & Miquelon							NA
St. Vincent & the Grenadines	29.14148041	29.6492321	29.929606	29.964139	29.735336	29.260907	29.62746824
Sudan	26.8068184	27.16817895	27.320543	27.263621	26.998035	26.542225	27.04326482
Suriname	28.78147943	29.20589635	29.40071	29.369909	29.118478	28.684082	29.11590399
Svalbard							NA
Swaziland	30.04383934	30.62039658	30.960599	31.042686	30.845888	30.39139	30.58103199
Sweden	25.42670439	25.95766088	26.439605	26.851812	27.172794	27.353547	26.48846362
Switzerland	24.00619082	24.58312804	25.080621	25.490031	25.803759	26.019464	25.05800672
Syria	30.46364862	31.2360262	31.741494	31.94892	31.828165	31.395676	31.31190479
Taiwan	23.88284525	24.45270066	24.914979	25.256622	25.464448	25.503018	NA
Tajikistan	26.64959081	27.43439958	28.043792	28.442381	28.59569	28.490295	27.67066115
Tanzania	24.61632819	24.71170824	24.628636	24.38484	23.999622	23.508987	24.4571265
Thailand	25.96992219	26.16762284	26.118549	25.835461	25.33239	24.64266	25.83300285
The Bahamas	29.99265516	30.55010495	30.864319	30.913226	30.675695	30.171008	30.52294791
The Gambia	25.29424918	25.50783775	25.520456	25.355721	25.038254	24.601758	25.31209529
Togo	25.10870823	25.31117568	25.326339	25.160954	24.822647	24.323822	25.11111575
Tokelau	35.45831406	35.5314948	35.266184	34.634124	33.608121	32.234049	NA
Tonga	35.91207358	36.05300921	35.818984	35.188262	34.140183	32.730865	35.37568063
Trinidad & Tobago	29.78598617	30.29241146	30.53461	30.503309	30.190203	29.626166	30.20155912
Tunisia	28.32202372	28.85915113	29.140412	29.135885	28.816818	28.189557	28.78731145
Turkey	30.02050009	30.91535289	31.53678	31.848348	31.813913	31.426331	31.09896827
Turkmenistan	26.97943055	27.75826014	28.3797	28.81895	29.052124	29.078251	28.03579753
Turks & Caicos Is.							NA
Tuvalu	33.55712843	34.03807802	34.166426	33.904645	33.216138	32.126387	NA
Uganda	23.49974155	23.54542612	23.474836	23.308238	23.066779	22.764997	23.39133827
Ukraine	27.19179797	27.75001744	28.111274	28.264236	28.198494	27.926067	27.8922573
United Arab Emirates	31.10683093	31.48510192	31.539553	31.262471	30.6476	29.746135	31.25786767
United Kingdom	27.54737949	27.97784982	28.303441	28.523389	28.635805	28.61568	28.23106343
United States	29.68181405	30.23654047	30.614841	30.782393	30.705162	30.372997	30.39528623
Uruguay	28.00583123	28.73509079	29.287709	29.656478	29.835355	29.85282	29.12680121
Uzbekistan	27.83049744	28.68181271	29.355387	29.81102	30.009484	29.941825	28.98169663

Vanuatu	28.05007139	28.32810391	28.35089	28.108254	27.590943	26.814909	28.02306247
Vatican City							#DIV/0!
Venezuela	27.85837063	28.22621107	28.385999	28.337521	28.081154	27.634309	28.1225049
Vietnam	22.13207774	22.3024695	22.314451	22.189157	21.94882	21.606739	22.170151
Virgin Is.							NA
Wake I.							NA
Wallis & Futuna							NA
West Bank							NA
Western Sahara							NA
Yemen	24.82301021	25.02774146	25.02154	24.830183	24.48058	24.008511	24.79208635
Zambia	24.14876691	24.52135817	24.753873	24.833527	24.747248	24.456095	24.48923666
Zimbabwe	25.89742143	26.23958359	26.433822	26.486719	26.406625	26.233014	26.20979698

Table S8 - Objective Multivariable Apnea Risk Index (objective MAP index) of male population age 40 ≤ 69 in 2013.

Objective multivariable apnea risk index (objective MAP index) of male population ages 40 ≤ 69 in 2013, sorted by objective MAP index in descending order (empty cells: no data available from respective source).

- Objective MAP index as: objective MAP index = $e^x / (1+e^x)$ with x= - 8.160+0.163*BMI + 0.032 * Age + 1.27 * Sex; (sex: male = 1, female = 0).
- Sources for objective MAP index: Maislin et al. "A survey screen for prediction of apnea", Sleep 1995; Khajeh-Mehrizi A. et al. "Diagnostic Accuracy of the Multivariable Apnea Prediction (MAP) Index as a Screening Tool for Obstructive Sleep Apnea", JSS 2016 [5,6].
- BMI as: weighted mean BMI of male population ages 40 to ≤ 69.

Age as: weighted mean age of male population ages 40 to ≤ 69.

	Country	Objective MAP Index	# of OSA Publications 1900 to 2018	Ranking regarding # of Publications	x of objective MAP Index	e^x of objective MAP Index	$1+e^x$ of objective MAP Index
1	Samoa	6.37E-01	0	-	5.61E-01	1.75E+00	2.75E+00
2	Tonga	6.26E-01	0	-	5.17E-01	1.68E+00	2.68E+00
3	Egypt	5.36E-01	113	36	1.44E-01	1.15E+00	2.15E+00
4	Micronesia	5.22E-01	0	-	8.81E-02	1.09E+00	2.09E+00
5	Kiribati	4.94E-01	0	-	-2.56E-02	9.75E-01	1.97E+00
6	Jordan	4.91E-01	21	57	-3.69E-02	9.64E-01	1.96E+00
7	Belize	4.80E-01	0	-	-7.97E-02	9.23E-01	1.92E+00
8	Kuwait	4.77E-01	8	66	-9.25E-02	9.12E-01	1.91E+00
9	St. Lucia	4.70E-01	0	-	-1.21E-01	8.86E-01	1.89E+00
10	Libya	4.65E-01	1	97	-1.39E-01	8.71E-01	1.87E+00
11	Syria	4.63E-01	1	97	-1.49E-01	8.62E-01	1.86E+00
12	Turkey	4.59E-01	1,093	10	-1.66E-01	8.47E-01	1.85E+00
13	Qatar	4.54E-01	21	57	-1.83E-01	8.33E-01	1.83E+00
14	South Africa	4.53E-01	23	52	-1.90E-01	8.27E-01	1.83E+00
15	Iraq	4.52E-01	5	72	-1.92E-01	8.25E-01	1.83E+00
16	Jamaica	4.49E-01	1	97	-2.05E-01	8.15E-01	1.81E+00
17	United States	4.44E-01	11,611	1	-2.25E-01	7.98E-01	1.80E+00
18	Saudi Arabia	4.38E-01	128	32	-2.51E-01	7.78E-01	1.78E+00
19	The Bahamas	4.36E-01	0	-	-2.58E-01	7.73E-01	1.77E+00
20	Swaziland	4.34E-01	1	97	-2.67E-01	7.65E-01	1.77E+00
21	Barbados	4.33E-01	1	97	-2.70E-01	7.63E-01	1.76E+00
22	Fiji	4.30E-01	0	-	-2.81E-01	7.55E-01	1.76E+00
23	Puerto Rico	4.30E-01	0	-	-2.81E-01	7.55E-01	1.76E+00
24	Trinidad & Tobago	4.30E-01	0	-	-2.82E-01	7.54E-01	1.75E+00
25	United Arab Emirates	4.29E-01	23	52	-2.88E-01	7.50E-01	1.75E+00
26	French Polynesia	4.22E-01	2	87	-3.15E-01	7.30E-01	1.73E+00

27	Georgia	4.18E-01	3	80	-3.30E-01	7.19E-01	1.72E+00
28	Grenada	4.15E-01	2	87	-3.44E-01	7.09E-01	1.71E+00
29	Moldova	4.13E-01	4	77	-3.50E-01	7.05E-01	1.70E+00
30	Seychelles	4.07E-01	0	-	-3.76E-01	6.87E-01	1.69E+00
31	Chile	4.07E-01	114	35	-3.77E-01	6.86E-01	1.69E+00
32	Mexico	4.03E-01	77	42	-3.94E-01	6.74E-01	1.67E+00
33	Azerbaijan	4.01E-01	0	-	-4.02E-01	6.69E-01	1.67E+00
34	Armenia	4.00E-01	1	97	-4.04E-01	6.68E-01	1.67E+00
35	St. Vincent & the Grenadines	3.99E-01	0	-	-4.08E-01	6.65E-01	1.66E+00
36	Lebanon	3.99E-01	24	51	-4.09E-01	6.65E-01	1.66E+00
37	Malta	3.97E-01	3	80	-4.18E-01	6.58E-01	1.66E+00
38	El Salvador	3.96E-01	0	-	-4.21E-01	6.56E-01	1.66E+00
39	Argentina	3.95E-01	85	39	-4.27E-01	6.52E-01	1.65E+00
40	Nicaragua	3.93E-01	0	-	-4.34E-01	6.48E-01	1.65E+00
41	Costa Rica	3.92E-01	0	-	-4.37E-01	6.46E-01	1.65E+00
42	Uruguay	3.90E-01	5	72	-4.46E-01	6.40E-01	1.64E+00
43	Belarus	3.88E-01	3	80	-4.56E-01	6.34E-01	1.63E+00
44	Croatia	3.87E-01	55	44	-4.60E-01	6.31E-01	1.63E+00
45	Greece	3.87E-01	466	17	-4.61E-01	6.31E-01	1.63E+00
46	Russia	3.86E-01	147	29	-4.65E-01	6.28E-01	1.63E+00
47	New Zealand	3.86E-01	118	34	-4.65E-01	6.28E-01	1.63E+00
48	Czech Republic	3.81E-01	108	38	-4.87E-01	6.14E-01	1.61E+00
49	Guyana	3.79E-01	0	-	-4.94E-01	6.10E-01	1.61E+00
50	Israel	3.78E-01	448	20	-4.96E-01	6.09E-01	1.61E+00
51	Suriname	3.76E-01	0	-	-5.06E-01	6.03E-01	1.60E+00
52	Ecuador	3.75E-01	7	67	-5.11E-01	6.00E-01	1.60E+00
53	Romania	3.73E-01	112	37	-5.18E-01	5.96E-01	1.60E+00
54	Hungary	3.72E-01	78	41	-5.22E-01	5.94E-01	1.59E+00
55	Antigua & Barbuda	3.70E-01	0	-	-5.34E-01	5.87E-01	1.59E+00
56	Tunisia	3.69E-01	39	46	-5.39E-01	5.84E-01	1.58E+00
57	Uzbekistan	3.68E-01	1	97	-5.39E-01	5.83E-01	1.58E+00
58	Panama	3.68E-01	0	-	-5.41E-01	5.82E-01	1.58E+00
59	Latvia	3.64E-01	6	71	-5.57E-01	5.73E-01	1.57E+00
60	Bolivia	3.64E-01	5	72	-5.58E-01	5.72E-01	1.57E+00
61	Cyprus	3.61E-01	7	67	-5.71E-01	5.65E-01	1.57E+00
62	Honduras	3.61E-01	0	-	-5.73E-01	5.64E-01	1.56E+00
63	United Kingdom	3.60E-01	1,375	7	-5.73E-01	5.64E-01	1.56E+00
64	Dominican Republic	3.60E-01	0	-	-5.74E-01	5.63E-01	1.56E+00

65	Kyrgyzstan	3.59E-01	22	55	-5.78E-01	5.61E-01	1.56E+00
66	Poland	3.58E-01	321	21	-5.83E-01	5.58E-01	1.56E+00
67	Ireland	3.58E-01	191	25	-5.85E-01	5.57E-01	1.56E+00
68	Iran	3.58E-01	119	33	-5.86E-01	5.57E-01	1.56E+00
69	Australia	3.57E-01	1,534	4	-5.88E-01	5.56E-01	1.56E+00
70	Canada	3.55E-01	1,947	2	-5.98E-01	5.50E-01	1.55E+00
71	Kazakhstan	3.54E-01	2	87	-6.00E-01	5.49E-01	1.55E+00
72	Lithuania	3.52E-01	23	52	-6.12E-01	5.42E-01	1.54E+00
73	Serbia	3.50E-01	38	47	-6.20E-01	5.38E-01	1.54E+00
74	Slovenia	3.50E-01	21	57	-6.21E-01	5.37E-01	1.54E+00
75	Guatemala	3.50E-01	0	-	-6.21E-01	5.37E-01	1.54E+00
76	Bulgaria	3.49E-01	26	49	-6.23E-01	5.36E-01	1.54E+00
77	Estonia	3.46E-01	15	60	-6.39E-01	5.28E-01	1.53E+00
78	Paraguay	3.44E-01	1	97	-6.43E-01	5.26E-01	1.53E+00
79	Ukraine	3.44E-01	13	62	-6.46E-01	5.24E-01	1.52E+00
80	Lesotho	3.44E-01	0	-	-6.46E-01	5.24E-01	1.52E+00
81	Peru	3.43E-01	25	50	-6.49E-01	5.23E-01	1.52E+00
82	Venezuela	3.41E-01	3	80	-6.57E-01	5.18E-01	1.52E+00
83	Finland	3.41E-01	289	22	-6.57E-01	5.18E-01	1.52E+00
84	Albania	3.40E-01	2	87	-6.63E-01	5.15E-01	1.52E+00
85	Colombia	3.39E-01	37	48	-6.68E-01	5.13E-01	1.51E+00
86	Brazil	3.38E-01	1,015	11	-6.73E-01	5.10E-01	1.51E+00
87	Vanuatu	3.37E-01	0	-	-6.78E-01	5.07E-01	1.51E+00
88	Macedonia	3.36E-01	1	97	-6.79E-01	5.07E-01	1.51E+00
89	Algeria	3.36E-01	2	87	-6.82E-01	5.06E-01	1.51E+00
90	Norway	3.35E-01	179	27	-6.85E-01	5.04E-01	1.50E+00
91	Slovakia	3.35E-01	42	45	-6.87E-01	5.03E-01	1.50E+00
92	Germany	3.35E-01	1,456	5	-6.87E-01	5.03E-01	1.50E+00
93	Turkmenistan	3.33E-01	0	-	-6.97E-01	4.98E-01	1.50E+00
94	Morocco	3.32E-01	7	67	-7.00E-01	4.97E-01	1.50E+00
95	Brunei	3.31E-01	0	-	-7.04E-01	4.95E-01	1.49E+00
96	Solomon Is.	3.25E-01	0	-	-7.31E-01	4.82E-01	1.48E+00
97	Mauritania	3.24E-01	0	-	-7.35E-01	4.80E-01	1.48E+00
98	Mongolia	3.24E-01	0	-	-7.38E-01	4.78E-01	1.48E+00
99	Botswana	3.21E-01	0	-	-7.51E-01	4.72E-01	1.47E+00
100	Tajikistan	3.19E-01	0	-	-7.56E-01	4.69E-01	1.47E+00
101	Mauritius	3.19E-01	0	-	-7.56E-01	4.69E-01	1.47E+00
102	Belgium	3.19E-01	492	16	-7.58E-01	4.69E-01	1.47E+00
103	Bosnia & Herzegovina	3.19E-01	1	97	-7.59E-01	4.68E-01	1.47E+00

104	Maldives	3.18E-01	0	-	-7.61E-01	4.67E-01	1.47E+00
105	Gabon	3.17E-01	0	-	-7.69E-01	4.64E-01	1.46E+00
106	Portugal	3.15E-01	223	23	-7.76E-01	4.60E-01	1.46E+00
107	Luxembourg	3.11E-01	2	87	-7.93E-01	4.52E-01	1.45E+00
108	Cuba	3.11E-01	1	97	-7.97E-01	4.51E-01	1.45E+00
109	Malaysia	3.06E-01	56	43	-8.18E-01	4.41E-01	1.44E+00
110	Spain	3.05E-01	1,011	12	-8.21E-01	4.40E-01	1.44E+00
111	Iceland	3.05E-01	80	40	-8.24E-01	4.39E-01	1.44E+00
112	Oman	3.02E-01	14	61	-8.39E-01	4.32E-01	1.43E+00
113	Italy	3.01E-01	1,329	9	-8.41E-01	4.31E-01	1.43E+00
114	Sudan	3.01E-01	0	-	-8.44E-01	4.30E-01	1.43E+00
115	Sweden	3.00E-01	539	15	-8.46E-01	4.29E-01	1.43E+00
116	Netherlands	2.99E-01	450	18	-8.52E-01	4.27E-01	1.43E+00
117	Austria	2.83E-01	137	31	-9.31E-01	3.94E-01	1.39E+00
118	Namibia	2.81E-01	0	-	-9.37E-01	3.92E-01	1.39E+00
119	Sao Tome & Principe	2.81E-01	0	-	-9.38E-01	3.91E-01	1.39E+00
120	France	2.80E-01	1,374	8	-9.45E-01	3.89E-01	1.39E+00
121	Equatorial Guinea	2.74E-01	0	-	-9.74E-01	3.77E-01	1.38E+00
122	Cape Verde	2.69E-01	0	-	-1.00E+00	3.67E-01	1.37E+00
123	Zimbabwe	2.68E-01	0	-	-1.00E+00	3.66E-01	1.37E+00
124	Denmark	2.68E-01	164	28	-1.01E+00	3.65E-01	1.37E+00
125	Comoros	2.67E-01	0	-	-1.01E+00	3.64E-01	1.36E+00
126	Papua New Guinea	2.66E-01	0	-	-1.01E+00	3.63E-01	1.36E+00
127	Thailand	2.66E-01	145	30	-1.02E+00	3.62E-01	1.36E+00
128	Ghana	2.63E-01	0	-	-1.03E+00	3.58E-01	1.36E+00
129	Liberia	2.58E-01	0	-	-1.06E+00	3.47E-01	1.35E+00
130	Cameroon	2.57E-01	5	72	-1.06E+00	3.46E-01	1.35E+00
131	Djibouti	2.53E-01	0	-	-1.08E+00	3.39E-01	1.34E+00
132	Pakistan	2.51E-01	22	55	-1.10E+00	3.35E-01	1.33E+00
133	Switzerland	2.49E-01	450	18	-1.11E+00	3.31E-01	1.33E+00
134	The Gambia	2.44E-01	0	-	-1.13E+00	3.23E-01	1.32E+00
135	Senegal	2.39E-01	0	-	-1.16E+00	3.14E-01	1.31E+00
136	Benin	2.39E-01	1	97	-1.16E+00	3.13E-01	1.31E+00
137	Togo	2.37E-01	0	-	-1.17E+00	3.10E-01	1.31E+00
138	Bahrain	2.33E-01	2	87	-1.19E+00	3.04E-01	1.30E+00
139	Yemen	2.32E-01	1	97	-1.20E+00	3.01E-01	1.30E+00
140	Cote dIvory	2.31E-01	0	-	-1.20E+00	3.00E-01	1.30E+00
141	Angola	2.31E-01	0	-	-1.20E+00	3.00E-01	1.30E+00
142	Guinea-Bissau	2.30E-01	0	-	-1.21E+00	2.98E-01	1.30E+00

143	Kenya	2.30E-01	3	80	-1.21E+00	2.98E-01	1.30E+00
144	Bhutan	2.28E-01	0	-	-1.22E+00	2.96E-01	1.30E+00
145	Congo	2.28E-01	1	97	-1.22E+00	2.95E-01	1.29E+00
146	Haiti	2.27E-01	0	-	-1.22E+00	2.94E-01	1.29E+00
147	North Korea	2.27E-01	0	-	-1.23E+00	2.93E-01	1.29E+00
148	Nigeria	2.26E-01	11	64	-1.23E+00	2.92E-01	1.29E+00
149	Afghanistan	2.25E-01	0	-	-1.24E+00	2.90E-01	1.29E+00
150	Singapore	2.24E-01	183	26	-1.24E+00	2.89E-01	1.29E+00
151	Somalia	2.24E-01	0	-	-1.24E+00	2.88E-01	1.29E+00
152	Sri Lanka	2.24E-01	5	72	-1.24E+00	2.88E-01	1.29E+00
153	China	2.22E-01	1,758	3	-1.26E+00	2.85E-01	1.28E+00
154	Tanzania	2.18E-01	2	87	-1.28E+00	2.79E-01	1.28E+00
155	Zambia	2.15E-01	0	-	-1.29E+00	2.75E-01	1.27E+00
156	South Korea	2.15E-01	654	13	-1.29E+00	2.75E-01	1.27E+00
157	Central African Republic	2.12E-01	0	-	-1.31E+00	2.69E-01	1.27E+00
158	Indonesia	2.11E-01	10	65	-1.32E+00	2.68E-01	1.27E+00
159	Philippines	2.08E-01	13	62	-1.34E+00	2.63E-01	1.26E+00
160	Sierra Leone	2.06E-01	0	-	-1.35E+00	2.59E-01	1.26E+00
161	Guinea	2.05E-01	0	-	-1.36E+00	2.58E-01	1.26E+00
162	Myanmar	2.05E-01	4	77	-1.36E+00	2.58E-01	1.26E+00
163	Mozambique	2.03E-01	1	97	-1.37E+00	2.55E-01	1.26E+00
164	Laos	1.96E-01	0	-	-1.41E+00	2.44E-01	1.24E+00
165	Mali	1.92E-01	0	-	-1.44E+00	2.38E-01	1.24E+00
166	Japan	1.91E-01	1,413	6	-1.44E+00	2.37E-01	1.24E+00
167	Malawi	1.89E-01	1	97	-1.46E+00	2.33E-01	1.23E+00
168	Uganda	1.87E-01	0	-	-1.47E+00	2.29E-01	1.23E+00
169	Congo, DRC	1.81E-01	0	-	-1.51E+00	2.20E-01	1.22E+00
170	Nepal	1.77E-01	2	87	-1.54E+00	2.15E-01	1.21E+00
171	Niger	1.73E-01	0	-	-1.56E+00	2.09E-01	1.21E+00
172	India	1.72E-01	219	24	-1.57E+00	2.08E-01	1.21E+00
173	Cambodia	1.71E-01	0	-	-1.58E+00	2.06E-01	1.21E+00
174	Rwanda	1.69E-01	0	-	-1.59E+00	2.04E-01	1.20E+00
175	Chad	1.64E-01	0	-	-1.63E+00	1.97E-01	1.20E+00
176	Vietnam	1.61E-01	7	67	-1.65E+00	1.93E-01	1.19E+00
177	Burkina Faso	1.60E-01	1	97	-1.65E+00	1.91E-01	1.19E+00
178	Bangladesh	1.56E-01	3	80	-1.69E+00	1.85E-01	1.19E+00
179	Burundi	1.47E-01	0	-	-1.76E+00	1.72E-01	1.17E+00
180	Ethiopia	1.41E-01	0	-	-1.81E+00	1.64E-01	1.16E+00

181	Madagascar	1.41E-01	0	-	-1.81E+00	1.64E-01	1.16E+00
182	Virgin Is.	5.77E-03	0	-	-5.15E+00	5.80E-03	1.01E+00
183	Aruba	5.51E-03	1	97	-5.20E+00	5.54E-03	1.01E+00
184	Guam	5.38E-03	0	-	-5.22E+00	5.41E-03	1.01E+00
185	New Caledonia	5.33E-03	1	97	-5.23E+00	5.36E-03	1.01E+00
186	West Bank	5.14E-03	0	-	-5.27E+00	5.16E-03	1.01E+00
-	Taiwan		567	14			
-	Guadeloupe		4	77			
-	Liechtenstein		3	80			
-	Montenegro		2	87			
-	St. Kitts & Nevis		1	97			
-	San Marino		1	97			
-	Reunion		1	97			
-	Netherlands Antilles		1	97			
-	Martinique		1	97			
-	Andorra		1	97			
-	Western Sahara		0	-			
-	Wallis & Futuna		0	-			
-	Wake I.		0	-			
-	Vatican City		0	-			
-	Tuvalu		0	-			
-	Turks & Caicos Is.		0	-			
-	Tokelau		0	-			
-	Svalbard		0	-			
-	St. Pierre & Miquelon		0	-			
-	St. Helena		0	-			
-	Spratly Is.		0	-			
-	South Georgia & the South Sandwich Is.		0	-			
-	Pitcairn Is.		0	-			
-	Paracel Is.		0	-			
-	Palau		0	-			
-	Northern Mariana Is.		0	-			
-	Norfolk I.		0	-			
-	Niue		0	-			
-	Nauru		0	-			
-	Montserrat		0	-			
-	Monaco		0	-			
-	Midway Is.		0	-			

-	Mayotte		0	-			
-	Marshall Is.		0	-			
-	Juan De Nova I.		0	-			
-	Johnston Atoll		0	-			
-	Jersey		0	-			
-	Jarvis I.		0	-			
-	Jan Mayen		0	-			
-	Isle of Man		0	-			
-	Howland I.		0	-			
-	Heard I. & McDonald Is.		0	-			
-	Guernsey		0	-			
-	Greenland		0	-			
-	Glorioso Is.		0	-			
-	Gibraltar		0	-			
-	Gaza Strip		0	-			
-	French Southern & Antarctic Lands		0	-			
-	French Guiana		0	-			
-	Faroe Is.		0	-			
-	Falkland Is.		0	-			
-	Eritrea		0	-			
-	East Timor		0	-			
-	Dominica		0	-			
-	Cook Is.		0	-			
-	Cocos Is.		0	-			
-	Christmas I.		0	-			
-	Cayman Is.		0	-			
-	British Virgin Is.		0	-			
-	British Indian Ocean Territory		0	-			
-	Bouvet I.		0	-			
-	Bermuda		0	-			
-	Baker I.		0	-			
-	Antarctica		0	-			
-	Anguilla		0	-			
-	American Samoa		0	-			

Table S9 - Country-specific estimated population at risk (EPR) of OSA in 2013.

Country-specific estimated population at risk (EPR) for OSA in 2013: country-specific total number of obese males age 40 to ≤ 69, sorted by country-specific estimated population at risk in descending order (empty cells: no data available from respective source).
Source for age- and gender-specific obesity data in 2013: Ng, M. et al., The Lancet, Aug. 2014 [7].

	Country	EPR 2013	# of OSA Publications 1900-2018	Ranking regarding # of OSA Publications 1900-2018	Mean # Male Population Ages 40≤44 in 2013	Mean # Male Population Ages 44≤49 in 2013	Mean # Male Population Ages 50≤54 in 2013	Mean # Male Population Ages 55≤59 in 2013	Mean # Male Population Ages 60≤64 in 2013	Mean # Male Population Ages 65≤69 in 2013
1	United States	1.95E+07	11,611	1	3.62E+06	3.85E+06	3.84E+06	3.20E+06	2.94E+06	2.00E+06
2	China	1.21E+07	1,758	3	3.71E+06	2.89E+06	2.07E+06	1.45E+06	1.23E+06	7.24E+05
3	India	8.00E+06	219	24	1.85E+06	1.70E+06	1.56E+06	1.28E+06	9.89E+05	6.11E+05
4	Brazil	5.81E+06	1,015	11	1.48E+06	1.32E+06	1.22E+06	8.75E+05	5.63E+05	3.42E+05
5	Russia	4.79E+06	147	29	7.93E+05	8.95E+05	1.05E+06	1.00E+06	7.91E+05	2.58E+05
6	Mexico	4.27E+06	77	42	1.13E+06	1.15E+06	7.15E+05	5.59E+05	4.43E+05	2.74E+05
7	Germany	4.07E+06	1,456	5	6.80E+05	8.33E+05	7.78E+05	7.45E+05	6.48E+05	3.88E+05
8	United Kingdom	3.62E+06	1,375	7	6.22E+05	7.22E+05	6.40E+05	5.34E+05	6.59E+05	4.45E+05
9	Egypt	2.91E+06	113	36	6.87E+05	6.50E+05	5.82E+05	4.48E+05	3.21E+05	2.18E+05
10	Turkey	2.67E+06	1,093	10	6.04E+05	6.01E+05	5.19E+05	4.45E+05	2.84E+05	2.21E+05
11	Indonesia	2.02E+06	10	65	5.70E+05	5.51E+05	3.94E+05	3.08E+05	1.24E+05	7.51E+04
12	France	1.95E+06	1,374	8	3.53E+05	3.45E+05	3.26E+05	3.45E+05	3.73E+05	2.10E+05
13	Spain	1.87E+06	1,011	12	3.64E+05	3.97E+05	3.40E+05	2.93E+05	2.76E+05	2.03E+05
14	Saudi Arabia	1.71E+06	128	32	6.30E+05	4.54E+05	2.77E+05	2.08E+05	1.00E+05	4.33E+04

15	Pakistan	1.61E+06	22	55	3.29E+05	3.18E+05	3.83E+05	2.54E+05	1.84E+05	1.47E+05
16	Nigeria	1.60E+06	11	64	4.36E+05	3.69E+05	3.20E+05	2.24E+05	1.56E+05	9.73E+04
17	Iran	1.59E+06	119	33	4.12E+05	3.88E+05	3.05E+05	2.38E+05	1.57E+05	9.37E+04
18	Canada	1.58E+06	1,947	2	2.57E+05	3.26E+05	3.24E+05	2.66E+05	2.37E+05	1.75E+05
19	Poland	1.57E+06	321	21	2.23E+05	2.24E+05	3.25E+05	3.61E+05	2.88E+05	1.50E+05
20	Italy	1.46E+06	1,329	9	2.30E+05	2.73E+05	2.60E+05	2.21E+05	2.82E+05	1.90E+05
21	Argentina	1.35E+06	85	39	2.85E+05	2.45E+05	2.53E+05	2.32E+05	1.92E+05	1.46E+05
22	Ukraine	1.28E+06	13	62	1.69E+05	2.40E+05	2.99E+05	2.84E+05	1.93E+05	9.30E+04
23	Australia	1.19E+06	1,534	4	2.21E+05	2.40E+05	2.30E+05	1.95E+05	1.87E+05	1.17E+05
24	Colombia	1.11E+06	37	48	2.50E+05	2.56E+05	2.29E+05	1.76E+05	1.18E+05	7.96E+04
25	South Africa	1.06E+06	23	52	2.26E+05	2.30E+05	2.14E+05	1.90E+05	1.34E+05	6.69E+04
26	Thailand	9.85E+05	145	30	2.37E+05	2.35E+05	2.06E+05	1.57E+05	9.52E+04	5.56E+04
27	Algeria	9.48E+05	2	87	2.23E+05	2.18E+05	1.88E+05	1.49E+05	1.07E+05	6.37E+04
28	Romania	9.20E+05	112	37	1.66E+05	1.54E+05	1.45E+05	1.84E+05	1.64E+05	1.07E+05
29	Venezuela	8.70E+05	3	80	2.08E+05	2.17E+05	1.73E+05	1.27E+05	8.71E+04	5.73E+04
30	Iraq	7.81E+05	5	72	2.32E+05	1.99E+05	1.29E+05	1.01E+05	7.83E+04	4.15E+04
31	Peru	7.23E+05	25	50	1.96E+05	1.62E+05	1.19E+05	1.01E+05	8.50E+04	6.01E+04
32	Chile	7.09E+05	114	35	1.67E+05	1.37E+05	1.60E+05	1.18E+05	7.12E+04	5.55E+04
33	Morocco	6.62E+05	7	67	1.27E+05	1.64E+05	1.61E+05	1.10E+05	6.91E+04	3.13E+04
34	Japan	6.46E+05	1,413	6	1.46E+05	1.17E+05	1.16E+05	6.84E+04	8.99E+04	1.09E+05

35	Philippines	6.35E+05	13	62	2.09E+05	1.49E+05	9.46E+04	7.80E+04	6.81E+04	3.69E+04
36	United Arab Emirates	6.33E+05	23	52	2.83E+05	1.78E+05	1.04E+05	4.77E+04	1.55E+04	4.94E+03
37	Uzbekistan	6.09E+05	1	97	1.55E+05	1.41E+05	1.33E+05	9.68E+04	5.43E+04	2.81E+04
38	Netherlands	5.63E+05	450	18	1.01E+05	1.04E+05	9.54E+04	8.28E+04	1.11E+05	6.96E+04
39	Malaysia	5.51E+05	56	43	1.25E+05	1.27E+05	1.09E+05	8.77E+04	6.35E+04	3.86E+04
40	Sudan	4.68E+05	0	-	1.34E+05	1.16E+05	9.12E+04	5.97E+04	4.19E+04	2.64E+04
41	Myanmar	4.65E+05	4	77	1.01E+05	1.11E+05	9.23E+04	7.87E+04	5.21E+04	2.93E+04
42	Syria	4.54E+05	1	97	1.05E+05	1.08E+05	9.25E+04	7.13E+04	4.97E+04	2.75E+04
43	Greece	4.39E+05	466	17	8.23E+04	9.07E+04	8.57E+04	6.34E+04	6.39E+04	5.34E+04
44	Ecuador	4.36E+05	7	67	9.95E+04	9.25E+04	8.41E+04	5.96E+04	4.85E+04	5.23E+04
45	Hungary	4.30E+05	78	41	6.70E+04	5.90E+04	8.20E+04	1.05E+05	6.60E+04	5.06E+04
46	Czech Republic	4.22E+05	108	38	5.71E+04	7.28E+04	6.45E+04	7.66E+04	9.07E+04	5.98E+04
47	Cuba	4.07E+05	1	97	9.04E+04	9.61E+04	8.30E+04	5.70E+04	4.91E+04	3.09E+04
48	Serbia	4.06E+05	38	47	5.53E+04	6.82E+04	6.48E+04	8.07E+04	7.82E+04	5.88E+04
49	Bangladesh	4.05E+05	3	80	1.06E+05	1.12E+05	8.46E+04	4.90E+04	3.23E+04	2.06E+04
50	Portugal	4.01E+05	223	23	6.69E+04	8.06E+04	7.05E+04	7.44E+04	6.13E+04	4.70E+04
51	Belgium	3.75E+05	492	16	5.06E+04	6.79E+04	7.48E+04	6.60E+04	7.31E+04	4.26E+04
52	Belarus	3.69E+05	3	80	5.91E+04	7.19E+04	9.01E+04	6.76E+04	5.89E+04	2.15E+04
53	Sweden	3.34E+05	539	15	6.06E+04	5.82E+04	6.22E+04	4.95E+04	4.65E+04	5.67E+04
54	Kazakhstan	3.11E+05	2	87	5.67E+04	7.97E+04	9.26E+04	4.11E+04	2.39E+04	1.67E+04

55	Austria	3.07E+05	137	31	3.59E+04	6.60E+04	6.68E+04	5.12E+04	4.75E+04	3.93E+04
56	Jordan	2.71E+05	21	57	8.48E+04	6.93E+04	4.80E+04	2.95E+04	2.18E+04	1.79E+04
57	Taiwan	2.59E+05	567	14	7.75E+04	8.22E+04	4.05E+04	2.33E+04	2.35E+04	1.18E+04
58	South Korea	2.57E+05	654	13	7.65E+04	5.00E+04	4.24E+04	4.23E+04	2.59E+04	1.94E+04
59	Kuwait	2.40E+05	8	66	9.02E+04	6.24E+04	4.31E+04	2.56E+04	1.18E+04	7.30E+03
60	New Zealand	2.40E+05	118	34	4.44E+04	4.96E+04	4.54E+04	3.86E+04	3.57E+04	2.65E+04
61	Bulgaria	2.39E+05	26	49	4.20E+04	3.35E+04	3.95E+04	4.75E+04	4.72E+04	2.93E+04
62	Switzerland	2.30E+05	450	18	4.18E+04	4.34E+04	4.11E+04	3.61E+04	3.41E+04	3.38E+04
63	Ireland	2.27E+05	191	25	4.66E+04	4.44E+04	4.36E+04	3.71E+04	3.32E+04	2.23E+04
64	Lebanon	2.26E+05	24	51	4.25E+04	5.94E+04	5.32E+04	3.21E+04	2.25E+04	1.62E+04
65	Afghanistan	2.18E+05	0	-	6.24E+04	5.07E+04	4.15E+04	3.08E+04	1.99E+04	1.22E+04
66	Dominican Republic	2.16E+05	0	-	6.01E+04	4.56E+04	4.47E+04	3.03E+04	1.92E+04	1.59E+04
67	Puerto Rico	2.13E+05	0	-	4.42E+04	4.30E+04	4.01E+04	3.46E+04	2.87E+04	2.26E+04
68	Tunisia	2.11E+05	39	46	5.79E+04	5.64E+04	4.88E+04	2.91E+04	1.20E+04	7.24E+03
69	Finland	2.11E+05	289	22	2.95E+04	4.15E+04	4.31E+04	3.76E+04	3.96E+04	2.00E+04
70	Cameroon	2.10E+05	5	72	5.81E+04	4.90E+04	3.82E+04	2.87E+04	2.21E+04	1.43E+04
71	Israel	2.10E+05	448	20	3.72E+04	3.25E+04	3.44E+04	3.63E+04	3.97E+04	2.98E+04
72	Ethiopia	2.01E+05	0	-	6.39E+04	4.33E+04	3.00E+04	2.02E+04	2.45E+04	1.94E+04
73	Libya	1.99E+05	1	97	6.17E+04	4.91E+04	3.53E+04	2.58E+04	1.67E+04	1.06E+04
74	Ghana	1.94E+05	0	-	4.74E+04	4.33E+04	3.87E+04	2.44E+04	2.52E+04	1.53E+04

75	Denmark	1.91E+05	164	28	3.23E+04	3.39E+04	3.39E+04	2.63E+04	3.41E+04	3.04E+04
76	Congo, DRC	1.87E+05	0	-	4.76E+04	5.03E+04	3.93E+04	1.89E+04	1.49E+04	1.62E+04
77	Kenya	1.84E+05	3	80	5.18E+04	3.97E+04	3.21E+04	2.75E+04	2.07E+04	1.22E+04
78	Azerbaijan	1.83E+05	0	-	3.60E+04	3.75E+04	4.39E+04	3.70E+04	2.14E+04	7.28E+03
79	Qatar	1.82E+05	21	57	7.79E+04	4.82E+04	3.01E+04	1.66E+04	6.23E+03	2.53E+03
80	Guatemala	1.81E+05	0	-	5.56E+04	4.69E+04	3.18E+04	2.75E+04	1.30E+04	6.10E+03
81	Paraguay	1.75E+05	1	97	4.17E+04	3.90E+04	3.30E+04	2.76E+04	2.07E+04	1.35E+04
82	Moldova	1.73E+05	4	77	2.47E+04	3.16E+04	3.61E+04	3.23E+04	3.25E+04	1.54E+04
83	Bolivia	1.68E+05	5	72	4.34E+04	3.65E+04	2.90E+04	2.45E+04	2.01E+04	1.48E+04
84	Norway	1.66E+05	179	27	4.21E+04	3.53E+04	2.65E+04	2.13E+04	2.44E+04	1.59E+04
85	Croatia	1.63E+05	55	44	2.62E+04	2.50E+04	3.55E+04	2.73E+04	3.16E+04	1.71E+04
86	Slovakia	1.63E+05	42	45	2.42E+04	2.50E+04	3.00E+04	3.74E+04	2.81E+04	1.79E+04
87	Bosnia & Herzegovina	1.54E+05	1	97	2.40E+04	2.50E+04	2.79E+04	3.29E+04	2.64E+04	1.76E+04
88	Oman	1.49E+05	14	61	5.23E+04	3.86E+04	2.47E+04	1.81E+04	1.02E+04	5.20E+03
89	Nepal	1.40E+05	2	87	3.30E+04	3.26E+04	2.82E+04	1.92E+04	1.58E+04	1.16E+04
90	Costa Rica	1.38E+05	0	-	2.32E+04	3.19E+04	2.78E+04	2.36E+04	1.78E+04	1.40E+04
91	El Salvador	1.38E+05	0	-	3.24E+04	3.05E+04	2.56E+04	2.05E+04	1.59E+04	1.27E+04
92	Honduras	1.35E+05	0	-	3.55E+04	3.14E+04	2.50E+04	2.00E+04	1.41E+04	8.62E+03
93	Vietnam	1.34E+05	7	67	4.00E+04	3.39E+04	2.46E+04	1.65E+04	9.03E+03	1.04E+04
94	Cote dIvory	1.32E+05	0	-	3.23E+04	2.72E+04	3.48E+04	2.17E+04	1.17E+04	4.32E+03

95	Tanzania	1.31E+05	2	87	3.83E+04	3.05E+04	2.42E+04	1.77E+04	1.25E+04	8.23E+03
96	Sri Lanka	1.27E+05	5	72	3.03E+04	2.42E+04	2.11E+04	2.19E+04	1.99E+04	9.73E+03
97	Georgia	1.27E+05	3	80	2.57E+04	2.62E+04	2.80E+04	2.36E+04	1.41E+04	9.13E+03
98	Turkmenistan	1.23E+05	0	-	3.06E+04	2.93E+04	2.63E+04	1.94E+04	1.11E+04	5.72E+03
99	Nicaragua	1.18E+05	0	-	3.18E+04	2.69E+04	2.30E+04	1.90E+04	1.09E+04	6.23E+03
100	Angola	1.16E+05	0	-	3.14E+04	2.78E+04	2.12E+04	1.55E+04	1.30E+04	7.15E+03
101	Uruguay	1.16E+05	5	72	2.04E+04	2.10E+04	2.21E+04	2.20E+04	1.77E+04	1.28E+04
102	Yemen	1.16E+05	1	97	2.99E+04	2.41E+04	2.14E+04	1.89E+04	1.36E+04	7.99E+03
103	Mozambique	1.12E+05	1	97	2.68E+04	2.43E+04	2.20E+04	1.74E+04	1.28E+04	9.18E+03
104	Mali	1.12E+05	0	-	3.43E+04	2.56E+04	1.92E+04	1.53E+04	1.12E+04	6.81E+03
105	Benin	1.09E+05	1	97	3.03E+04	2.60E+04	2.11E+04	1.53E+04	1.04E+04	5.70E+03
106	Kyrgyzstan	9.51E+04	22	55	2.22E+04	2.25E+04	2.23E+04	1.56E+04	8.81E+03	3.81E+03
107	Lithuania	9.44E+04	23	52	1.23E+04	1.86E+04	2.44E+04	1.41E+04	1.38E+04	1.12E+04
108	Slovenia	8.80E+04	21	57	1.44E+04	1.18E+04	1.76E+04	1.89E+04	1.54E+04	9.90E+03
109	Latvia	8.46E+04	6	71	1.36E+04	1.71E+04	1.95E+04	1.46E+04	1.19E+04	7.91E+03
110	Armenia	8.40E+04	1	97	1.46E+04	1.67E+04	2.06E+04	1.68E+04	1.02E+04	5.02E+03
111	Bahrain	8.24E+04	2	87	3.04E+04	2.29E+04	1.48E+04	8.83E+03	3.46E+03	1.89E+03
112	Chad	8.19E+04	0	-	2.37E+04	1.86E+04	1.53E+04	1.17E+04	8.26E+03	4.34E+03
113	Albania	7.59E+04	2	87	1.35E+04	1.68E+04	1.49E+04	1.29E+04	1.02E+04	7.59E+03
114	Zambia	6.50E+04	0	-	2.11E+04	1.47E+04	1.07E+04	7.69E+03	6.18E+03	4.71E+03

115	Singapore	6.44E+04	183	26	1.32E+04	1.42E+04	1.37E+04	1.09E+04	8.67E+03	3.60E+03
116	Haiti	6.37E+04	0	-	1.65E+04	1.49E+04	1.24E+04	9.50E+03	6.54E+03	3.88E+03
117	Jamaica	6.19E+04	1	97	1.49E+04	1.37E+04	1.15E+04	9.43E+03	7.20E+03	5.13E+03
118	Niger	6.14E+04	0	-	8.48E+03	1.43E+04	1.54E+04	1.16E+04	7.35E+03	4.24E+03
119	Mongolia	5.89E+04	0	-	1.67E+04	1.55E+04	1.26E+04	7.46E+03	4.47E+03	2.19E+03
120	Liberia	5.82E+04	0	-	1.70E+04	1.38E+04	1.10E+04	7.92E+03	5.39E+03	3.18E+03
121	Madagascar	5.72E+04	0	-	1.58E+04	1.30E+04	1.06E+04	8.52E+03	5.91E+03	3.39E+03
122	Uganda	5.66E+04	0	-	2.00E+04	1.28E+04	7.40E+03	7.00E+03	5.32E+03	4.05E+03
123	Burkina Faso	5.58E+04	1	97	1.67E+04	1.35E+04	1.04E+04	7.28E+03	4.80E+03	3.11E+03
124	Estonia	5.31E+04	15	60	1.00E+04	9.55E+03	1.07E+04	9.21E+03	8.88E+03	4.70E+03
125	Trinidad & Tobago	5.18E+04	0	-	1.10E+04	1.00E+04	1.04E+04	9.48E+03	7.17E+03	3.89E+03
126	Zimbabwe	5.12E+04	0	-	1.14E+04	1.14E+04	1.01E+04	9.48E+03	5.31E+03	3.59E+03
127	Papua New Guinea	4.73E+04	0	-	1.45E+04	1.11E+04	8.80E+03	6.42E+03	4.23E+03	2.24E+03
128	Mauritania	4.62E+04	0	-	1.19E+04	1.01E+04	8.39E+03	7.74E+03	5.25E+03	2.81E+03
129	Senegal	4.50E+04	0	-	1.19E+04	9.59E+03	8.16E+03	7.02E+03	5.15E+03	3.16E+03
130	Panama	4.41E+04	0	-	1.08E+04	1.05E+04	8.36E+03	6.64E+03	4.60E+03	3.23E+03
131	Guinea	4.31E+04	0	-	1.10E+04	9.52E+03	8.57E+03	6.96E+03	4.59E+03	2.50E+03
132	Tajikistan	3.95E+04	0	-	9.60E+03	9.79E+03	9.12E+03	6.15E+03	3.19E+03	1.63E+03
133	Northern Mariana Is.	3.76E+04	0	-						
134	Malawi	3.64E+04	1	97	1.12E+04	6.86E+03	5.31E+03	3.06E+03	2.51E+03	7.47E+03

135	Sierra Leone	3.30E+04	0	-	9.21E+03	7.49E+03	6.11E+03	4.64E+03	3.44E+03	2.10E+03
136	Cambodia	2.94E+04	0	-	8.41E+03	7.26E+03	5.51E+03	3.71E+03	2.85E+03	1.66E+03
137	Mauritius	2.91E+04	0	-	5.98E+03	6.36E+03	6.22E+03	4.86E+03	3.54E+03	2.11E+03
138	Cyprus	2.86E+04	7	67	4.73E+03	5.45E+03	5.75E+03	5.42E+03	4.39E+03	2.88E+03
139	Luxembourg	2.69E+04	2	87	4.84E+03	6.06E+03	5.14E+03	4.61E+03	3.74E+03	2.46E+03
140	Fiji	2.67E+04	0	-	4.99E+03	5.94E+03	5.73E+03	4.74E+03	3.11E+03	2.18E+03
141	Congo	2.57E+04	1	97	6.77E+03	7.08E+03	4.32E+03	2.66E+03	3.13E+03	1.73E+03
142	Gabon	2.46E+04	0	-	6.60E+03	5.36E+03	4.22E+03	3.41E+03	2.86E+03	2.11E+03
143	Somalia	2.21E+04	0	-	5.83E+03	5.06E+03	3.65E+03	3.37E+03	2.61E+03	1.56E+03
144	Malta	2.10E+04	3	80	3.40E+03	3.45E+03	4.01E+03	3.68E+03	3.40E+03	3.03E+03
145	Botswana	2.02E+04	0	-	5.64E+03	4.97E+03	4.06E+03	2.67E+03	1.76E+03	1.06E+03
146	Central African Republic	1.94E+04	0	-	5.12E+03	4.42E+03	3.52E+03	2.75E+03	2.23E+03	1.39E+03
147	Namibia	1.91E+04	0	-	5.25E+03	4.02E+03	3.90E+03	3.19E+03	1.84E+03	9.50E+02
148	Togo	1.79E+04	0	-	5.14E+03	4.11E+03	3.25E+03	2.44E+03	1.78E+03	1.20E+03
149	Laos	1.79E+04	0	-	6.29E+03	4.34E+03	2.55E+03	2.66E+03	1.44E+03	6.22E+02
150	Suriname	1.75E+04	0	-	4.46E+03	4.21E+03	3.69E+03	2.48E+03	1.64E+03	9.93E+02
151	Eritrea	1.70E+04	0	-	5.56E+03	3.31E+03	2.59E+03	2.58E+03	1.92E+03	1.08E+03
152	Iceland	1.46E+04	80	40	2.56E+03	2.77E+03	2.92E+03	2.52E+03	2.24E+03	1.58E+03
153	Solomon Is.	1.45E+04	0	-	4.37E+03	3.90E+03	3.08E+03	1.54E+03	1.00E+03	6.46E+02
154	Swaziland	1.42E+04	1	97	2.95E+03	3.18E+03	2.69E+03	2.27E+03	1.79E+03	1.36E+03

155	Guyana	1.38E+04	0	-	3.28E+03	3.43E+03	2.98E+03	2.12E+03	1.24E+03	7.01E+02
156	The Bahamas	1.35E+04	0	-	3.30E+03	3.02E+03	2.82E+03	2.09E+03	1.44E+03	8.71E+02
157	The Gambia	1.30E+04	0	-	3.59E+03	3.13E+03	2.38E+03	1.84E+03	1.26E+03	7.91E+02
158	Barbados	1.26E+04	1	97	2.54E+03	2.57E+03	2.59E+03	2.20E+03	1.65E+03	1.02E+03
159	Equatorial Guinea	1.12E+04	0	-	2.54E+03	2.93E+03	2.46E+03	1.69E+03	1.08E+03	5.07E+02
160	Lesotho	1.05E+04	0	-	3.11E+03	1.88E+03	1.75E+03	1.45E+03	1.37E+03	9.74E+02
161	Rwanda	1.02E+04	0	-	2.05E+03	2.03E+03	2.55E+03	1.74E+03	1.02E+03	7.76E+02
162	Djibouti	1.01E+04	0	-	2.67E+03	2.36E+03	1.89E+03	1.37E+03	1.01E+03	7.49E+02
163	Guam	9.91E+03	0	-	2.14E+03	2.27E+03	2.09E+03	1.53E+03	1.17E+03	7.15E+02
164	Samoa	9.17E+03	0	-	2.38E+03	2.19E+03	1.79E+03	1.39E+03	8.86E+02	5.34E+02
165	Belize	8.36E+03	0	-	2.37E+03	1.99E+03	1.64E+03	1.18E+03	7.45E+02	4.26E+02
166	Guinea-Bissau	7.61E+03	0	-	1.90E+03	1.64E+03	1.47E+03	1.23E+03	9.18E+02	4.56E+02
167	Virgin Is.	6.20E+03	0	-	1.03E+03	1.12E+03	1.19E+03	1.11E+03	9.40E+02	8.03E+02
168	American Samoa	5.46E+03	0	-	1.65E+03	1.33E+03	9.86E+02	7.30E+02	5.07E+02	2.63E+02
169	Tonga	5.17E+03	0	-	1.64E+03	1.04E+03	9.08E+02	7.89E+02	4.50E+02	3.34E+02
170	Burundi	5.14E+03	0	-	1.18E+03	1.17E+03	1.03E+03	8.97E+02	5.64E+02	2.95E+02
171	Maldives	4.81E+03	0	-	1.13E+03	1.30E+03	1.08E+03	7.07E+02	3.94E+02	2.10E+02
172	Bhutan	4.77E+03	0	-	1.30E+03	1.13E+03	8.54E+02	6.93E+02	4.50E+02	3.51E+02
173	Vanuatu	4.50E+03	0	-	1.11E+03	1.16E+03	1.02E+03	5.74E+02	4.05E+02	2.28E+02
174	Kiribati	4.25E+03	0	-	1.13E+03	1.13E+03	8.77E+02	5.54E+02	3.64E+02	2.03E+02

175	Cape Verde	3.92E+03	0	-	1.14E+03	1.00E+03	7.90E+02	5.12E+02	3.29E+02	1.47E+02
176	Brunei	3.87E+03	0	-	1.18E+03	9.08E+02	7.46E+02	5.48E+02	3.24E+02	1.71E+02
177	Comoros	3.40E+03	0	-	9.99E+02	8.30E+02	6.59E+02	4.59E+02	2.96E+02	1.53E+02
178	Seychelles	3.23E+03	0	-	1.08E+03	7.23E+02	6.36E+02	4.46E+02	2.56E+02	9.46E+01
179	Marshall Is.	2.53E+03	0	-	7.11E+02	6.57E+02	5.31E+02	3.15E+02	1.98E+02	1.17E+02
180	Greenland	2.44E+03	0	-	4.31E+02	6.13E+02	5.34E+02	3.99E+02	2.77E+02	1.82E+02
181	Andorra	2.34E+03	1	97	4.09E+02	4.50E+02	4.22E+02	3.95E+02	4.04E+02	2.60E+02
182	Antigua & Barbuda	2.25E+03	0	-	6.00E+02	5.89E+02	4.23E+02	3.33E+02	2.02E+02	1.03E+02
183	Bermuda	1.94E+03	0	-	4.69E+02	4.41E+02	3.86E+02	2.90E+02	2.18E+02	1.39E+02
184	Grenada	1.91E+03	2	87	4.42E+02	4.12E+02	4.23E+02	3.00E+02	2.11E+02	1.23E+02
185	Dominica	1.41E+03	0	-	3.35E+02	3.24E+02	2.81E+02	2.08E+02	1.58E+02	1.01E+02
186	Sao Tome & Principe	1.35E+03	0	-	3.98E+02	3.23E+02	2.95E+02	1.74E+02	1.02E+02	5.66E+01
-	St. Kitts & Nevis		1	97						
-	San Marino		1	97						
-	Reunion		1	97						
-	New Caledonia		1	97						
-	Netherlands Antilles		1	97						
-	Martinique		1	97						
-	Macedonia		1	97						
-	Aruba		1	97						
-	Montenegro		2	87						
-	French Polynesia		2	87						
-	Liechtenstein		3	80						
-	Guadeloupe		4	77						
-	Western Sahara		0	-						
-	West Bank		0	-						
-	Wallis & Futuna		0	-						

-	Wake I.		0	-						
-	Vatican City		0	-						
-	Tuvalu		0	-						
-	Turks & Caicos Is.		0	-						
-	Tokelau		0	-						
-	Svalbard		0	-						
-	St. Vincent & the Grenadines		0	-						
-	St. Pierre & Miquelon		0	-						
-	St. Lucia		0	-						
-	St. Helena		0	-						
-	Spratly Is.		0	-						
-	South Georgia & the South Sandwich Is.		0	-						
-	Pitcairn Is.		0	-						
-	Paracel Is.		0	-						
-	Palau		0	-						
-	North Korea		0	-	1.08E+04	9.11E+03	6.76E+03	5.18E+03	2.66E+03	3.09E+03
-	Norfolk I.		0	-						
-	Niue		0	-						
-	Nauru		0	-						
-	Montserrat		0	-						
-	Monaco		0	-						
-	Midway Is.		0	-						
-	Micronesia		0	-						
-	Mayotte		0	-						
-	Juan De Nova I.		0	-						
-	Johnston Atoll		0	-						
-	Jersey		0	-						
-	Jarvis I.		0	-						
-	Jan Mayen		0	-						
-	Isle of Man		0	-						
-	Howland I.		0	-						
-	Heard I. & McDonald Is.		0	-						
-	Guernsey		0	-						

-	Glorioso Is.		0	-							
-	Gibraltar		0	-							
-	Gaza Strip		0	-							
-	French Southern & Antarctic Lands		0	-							
-	French Guiana		0	-							
-	Faroe Is.		0	-							
-	Falkland Is.		0	-							
-	East Timor		0	-							
-	Cook Is.		0	-							
-	Cocos Is.		0	-							
-	Christmas I.		0	-							
-	Cayman Is.		0	-							
-	British Virgin Is.		0	-							
-	British Indian Ocean Territory		0	-							
-	Bouvet I.		0	-							
-	Baker I.		0	-							
-	Antarctica		0	-							
-	Anguilla		0	-							

Table S10 - Country-specific ratios R3 and R4.

Country-specific ratios R3 (Number of OSA publications 1900-2013 / total number of obese males age 40 to ≤ 69 in 2013) and R4 (Number of OSA publications 1900-2018 / total number of obese males age 40 to ≤ 69 in 2013) sorted by estimated population at risk (EPR) in descending order.

	Country	EPR 2013	R3: # of OSA Publications 1900 to 2018/EPR	Ranking regarding R3	R4: # of OSA Publications 1900-2013/EPR	Ranking regarding R4
1	United States	1.95E+07	5.97E-04	21	3.74E-04	22
2	China	1.21E+07	1.46E-04	44	4.85E-05	44
3	India	8.00E+06	2.74E-05	73	9.63E-06	72
4	Brazil	5.81E+06	1.75E-04	41	8.71E-05	38
5	Russia	4.79E+06	3.07E-05	70	1.86E-05	59
6	Mexico	4.27E+06	1.80E-05	81	1.01E-05	70
7	Germany	4.07E+06	3.58E-04	29	2.23E-04	27
8	United Kingdom	3.62E+06	3.80E-04	28	2.36E-04	26
9	Egypt	2.91E+06	3.89E-05	65	1.72E-05	60
10	Turkey	2.67E+06	4.09E-04	27	1.88E-04	29
11	Indonesia	2.02E+06	4.95E-06	104	1.48E-06	85
12	France	1.95E+06	7.04E-04	20	4.43E-04	18
13	Spain	1.87E+06	5.40E-04	23	2.94E-04	24
14	Saudi Arabia	1.71E+06	7.48E-05	55	3.27E-05	52
15	Pakistan	1.61E+06	1.36E-05	88	4.96E-06	79
16	Nigeria	1.60E+06	6.87E-06	98	3.74E-06	81
17	Iran	1.59E+06	7.47E-05	56	1.69E-05	61
18	Canada	1.58E+06	1.23E-03	12	7.58E-04	10
19	Poland	1.57E+06	2.04E-04	38	1.02E-04	34
20	Italy	1.46E+06	9.13E-04	16	4.66E-04	17
21	Argentina	1.35E+06	6.28E-05	60	2.81E-05	53
22	Ukraine	1.28E+06	1.02E-05	91	3.91E-06	80
23	Australia	1.19E+06	1.29E-03	11	7.04E-04	12
24	Colombia	1.11E+06	3.34E-05	68	1.35E-05	63
25	South Africa	1.06E+06	2.17E-05	79	1.04E-05	69
26	Thailand	9.85E+05	1.47E-04	43	5.38E-05	42
27	Algeria	9.48E+05	2.11E-06	108	0.00E+00	-
28	Romania	9.20E+05	1.22E-04	46	2.39E-05	56
29	Venezuela	8.70E+05	3.45E-06	105	2.30E-06	82
30	Iraq	7.81E+05	6.40E-06	101	0.00E+00	-
31	Peru	7.23E+05	3.46E-05	67	1.25E-05	65
32	Chile	7.09E+05	1.61E-04	42	5.36E-05	43

33	Morocco	6.62E+05	1.06E-05	90	1.51E-06	84
34	Japan	6.46E+05	2.19E-03	5	1.52E-03	3
35	Philippines	6.35E+05	2.05E-05	80	1.42E-05	62
36	United Arab Emirates	6.33E+05	3.63E-05	66	7.90E-06	73
37	Uzbekistan	6.09E+05	1.64E-06	109	0.00E+00	-
38	Netherlands	5.63E+05	7.99E-04	19	3.83E-04	21
39	Malaysia	5.51E+05	1.02E-04	50	4.54E-05	48
40	Sudan	4.68E+05	0.00E+00	-	0.00E+00	-
41	Myanmar	4.65E+05	8.60E-06	95	0.00E+00	-
42	Syria	4.54E+05	2.20E-06	107	2.20E-06	83
43	Greece	4.39E+05	1.06E-03	14	6.33E-04	14
44	Ecuador	4.36E+05	1.60E-05	85	6.87E-06	76
45	Hungary	4.30E+05	1.82E-04	40	9.54E-05	37
46	Czech Republic	4.22E+05	2.56E-04	33	9.96E-05	36
47	Cuba	4.07E+05	2.46E-06	106	0.00E+00	-
48	Serbia	4.06E+05	9.36E-05	52	9.86E-06	71
49	Bangladesh	4.05E+05	7.42E-06	97	0.00E+00	-
50	Portugal	4.01E+05	5.57E-04	22	1.50E-04	31
51	Belgium	3.75E+05	1.31E-03	10	7.52E-04	11
52	Belarus	3.69E+05	8.13E-06	96	0.00E+00	-
53	Sweden	3.34E+05	1.62E-03	8	1.08E-03	6
54	Kazakhstan	3.11E+05	6.44E-06	100	0.00E+00	-
55	Austria	3.07E+05	4.47E-04	25	2.54E-04	25
56	Jordan	2.71E+05	7.74E-05	54	2.21E-05	58
57	Taiwan	2.59E+05	2.19E-03	4	9.58E-04	9
58	South Korea	2.57E+05	2.55E-03	3	1.15E-03	5
59	Kuwait	2.40E+05	3.33E-05	69	0.00E+00	-
60	New Zealand	2.40E+05	4.91E-04	24	3.12E-04	23
61	Bulgaria	2.39E+05	1.09E-04	48	2.51E-05	55
62	Switzerland	2.30E+05	1.95E-03	7	1.07E-03	7
63	Ireland	2.27E+05	8.41E-04	18	5.33E-04	15
64	Lebanon	2.26E+05	1.06E-04	49	3.98E-05	49
65	Afghanistan	2.18E+05	0.00E+00	-	0.00E+00	-
66	Dominican Republic	2.16E+05	0.00E+00	-	0.00E+00	-
67	Puerto Rico	2.13E+05	0.00E+00	-	0.00E+00	-
68	Tunisia	2.11E+05	1.84E-04	39	2.36E-05	57
69	Finland	2.11E+05	1.37E-03	9	9.94E-04	8
70	Cameroon	2.10E+05	2.38E-05	76	0.00E+00	-
71	Israel	2.10E+05	2.13E-03	6	1.58E-03	2

72	Ethiopia	2.01E+05	0.00E+00	-	0.00E+00	-
73	Libya	1.99E+05	5.02E-06	103	5.02E-06	78
74	Ghana	1.94E+05	0.00E+00	-	0.00E+00	-
75	Denmark	1.91E+05	8.58E-04	17	4.19E-04	20
76	Congo, DRC	1.87E+05	0.00E+00	-	0.00E+00	-
77	Kenya	1.84E+05	1.63E-05	83	5.44E-06	77
78	Azerbaijan	1.83E+05	0.00E+00	-	0.00E+00	-
79	Qatar	1.82E+05	1.16E-04	47	3.86E-05	50
80	Guatemala	1.81E+05	0.00E+00	-	0.00E+00	-
81	Paraguay	1.75E+05	5.70E-06	102	0.00E+00	-
82	Moldova	1.73E+05	2.32E-05	78	1.16E-05	68
83	Bolivia	1.68E+05	2.97E-05	71	1.19E-05	67
84	Norway	1.66E+05	1.08E-03	13	6.40E-04	13
85	Croatia	1.63E+05	3.38E-04	30	1.23E-04	32
86	Slovakia	1.63E+05	2.58E-04	32	1.17E-04	33
87	Bosnia & Herzegovina	1.54E+05	6.50E-06	99	0.00E+00	-
88	Oman	1.49E+05	9.39E-05	51	4.70E-05	47
89	Nepal	1.40E+05	1.42E-05	87	0.00E+00	-
90	Costa Rica	1.38E+05	0.00E+00	-	0.00E+00	-
91	El Salvador	1.38E+05	0.00E+00	-	0.00E+00	-
92	Honduras	1.35E+05	0.00E+00	-	0.00E+00	-
93	Vietnam	1.34E+05	5.21E-05	61	0.00E+00	-
94	Cote dIvory	1.32E+05	0.00E+00	-	0.00E+00	-
95	Tanzania	1.31E+05	1.52E-05	86	7.61E-06	75
96	Sri Lanka	1.27E+05	3.93E-05	63	0.00E+00	-
97	Georgia	1.27E+05	2.37E-05	77	7.89E-06	74
98	Turkmenistan	1.23E+05	0.00E+00	-	0.00E+00	-
99	Nicaragua	1.18E+05	0.00E+00	-	0.00E+00	-
100	Angola	1.16E+05	0.00E+00	-	0.00E+00	-
101	Uruguay	1.16E+05	4.31E-05	62	2.59E-05	54
102	Yemen	1.16E+05	8.63E-06	94	0.00E+00	-
103	Mozambique	1.12E+05	8.89E-06	93	0.00E+00	-
104	Mali	1.12E+05	0.00E+00	-	0.00E+00	-
105	Benin	1.09E+05	9.19E-06	92	0.00E+00	-
106	Kyrgyzstan	9.51E+04	2.31E-04	37	1.68E-04	30
107	Lithuania	9.44E+04	2.44E-04	35	8.47E-05	39
108	Slovenia	8.80E+04	2.39E-04	36	1.02E-04	35
109	Latvia	8.46E+04	7.09E-05	58	4.73E-05	46
110	Armenia	8.40E+04	1.19E-05	89	0.00E+00	-

111	Bahrain	8.24E+04	2.43E-05	75	1.21E-05	66
112	Chad	8.19E+04	0.00E+00	-	0.00E+00	-
113	Albania	7.59E+04	2.64E-05	74	1.32E-05	64
114	Zambia	6.50E+04	0.00E+00	-	0.00E+00	-
115	Singapore	6.44E+04	2.84E-03	2	1.23E-03	4
116	Haiti	6.37E+04	0.00E+00	-	0.00E+00	-
117	Jamaica	6.19E+04	1.62E-05	84	0.00E+00	-
118	Niger	6.14E+04	0.00E+00	-	0.00E+00	-
119	Mongolia	5.89E+04	0.00E+00	-	0.00E+00	-
120	Liberia	5.82E+04	0.00E+00	-	0.00E+00	-
121	Madagascar	5.72E+04	0.00E+00	-	0.00E+00	-
122	Uganda	5.66E+04	0.00E+00	-	0.00E+00	-
123	Burkina Faso	5.58E+04	1.79E-05	82	0.00E+00	-
124	Estonia	5.31E+04	2.82E-04	31	1.88E-04	28
125	Trinidad & Tobago	5.18E+04	0.00E+00	-	0.00E+00	-
126	Zimbabwe	5.12E+04	0.00E+00	-	0.00E+00	-
127	Papua New Guinea	4.73E+04	0.00E+00	-	0.00E+00	-
128	Mauritania	4.62E+04	0.00E+00	-	0.00E+00	-
129	Senegal	4.50E+04	0.00E+00	-	0.00E+00	-
130	Panama	4.41E+04	0.00E+00	-	0.00E+00	-
131	Guinea	4.31E+04	0.00E+00	-	0.00E+00	-
132	Tajikistan	3.95E+04	0.00E+00	-	0.00E+00	-
133	Northern Mariana Is.	3.76E+04	0.00E+00	-	0.00E+00	-
134	Malawi	3.64E+04	2.75E-05	72	0.00E+00	-
135	Sierra Leone	3.30E+04	0.00E+00	-	0.00E+00	-
136	Cambodia	2.94E+04	0.00E+00	-	0.00E+00	-
137	Mauritius	2.91E+04	0.00E+00	-	0.00E+00	-
138	Cyprus	2.86E+04	2.45E-04	34	6.99E-05	41
139	Luxembourg	2.69E+04	7.45E-05	57	3.72E-05	51
140	Fiji	2.67E+04	0.00E+00	-	0.00E+00	-
141	Congo	2.57E+04	3.89E-05	64	0.00E+00	-
142	Gabon	2.46E+04	0.00E+00	-	0.00E+00	-
143	Somalia	2.21E+04	0.00E+00	-	0.00E+00	-
144	Malta	2.10E+04	1.43E-04	45	4.77E-05	45
145	Botswana	2.02E+04	0.00E+00	-	0.00E+00	-
146	Central African Republic	1.94E+04	0.00E+00	-	0.00E+00	-
147	Namibia	1.91E+04	0.00E+00	-	0.00E+00	-
148	Togo	1.79E+04	0.00E+00	-	0.00E+00	-
149	Laos	1.79E+04	0.00E+00	-	0.00E+00	-

150	Suriname	1.75E+04	0.00E+00	-	0.00E+00	-
151	Eritrea	1.70E+04	0.00E+00	-	0.00E+00	-
152	Iceland	1.46E+04	5.49E-03	1	3.43E-03	1
153	Solomon Is.	1.45E+04	0.00E+00	-	0.00E+00	-
154	Swaziland	1.42E+04	7.02E-05	59	0.00E+00	-
155	Guyana	1.38E+04	0.00E+00	-	0.00E+00	-
156	The Bahamas	1.35E+04	0.00E+00	-	0.00E+00	-
157	The Gambia	1.30E+04	0.00E+00	-	0.00E+00	-
158	Barbados	1.26E+04	7.95E-05	53	7.95E-05	40
159	Equatorial Guinea	1.12E+04	0.00E+00	-	0.00E+00	-
160	Lesotho	1.05E+04	0.00E+00	-	0.00E+00	-
161	Rwanda	1.02E+04	0.00E+00	-	0.00E+00	-
162	Djibouti	1.01E+04	0.00E+00	-	0.00E+00	-
163	Guam	9.91E+03	0.00E+00	-	0.00E+00	-
164	Samoa	9.17E+03	0.00E+00	-	0.00E+00	-
165	Belize	8.36E+03	0.00E+00	-	0.00E+00	-
166	Guinea-Bissau	7.61E+03	0.00E+00	-	0.00E+00	-
167	Virgin Is.	6.20E+03	0.00E+00	-	0.00E+00	-
168	American Samoa	5.46E+03	0.00E+00	-	0.00E+00	-
169	Tonga	5.17E+03	0.00E+00	-	0.00E+00	-
170	Burundi	5.14E+03	0.00E+00	-	0.00E+00	-
171	Maldives	4.81E+03	0.00E+00	-	0.00E+00	-
172	Bhutan	4.77E+03	0.00E+00	-	0.00E+00	-
173	Vanuatu	4.50E+03	0.00E+00	-	0.00E+00	-
174	Kiribati	4.25E+03	0.00E+00	-	0.00E+00	-
175	Cape Verde	3.92E+03	0.00E+00	-	0.00E+00	-
176	Brunei	3.87E+03	0.00E+00	-	0.00E+00	-
177	Comoros	3.40E+03	0.00E+00	-	0.00E+00	-
178	Seychelles	3.23E+03	0.00E+00	-	0.00E+00	-
179	Marshall Is.	2.53E+03	0.00E+00	-	0.00E+00	-
180	Greenland	2.44E+03	0.00E+00	-	0.00E+00	-
181	Andorra	2.34E+03	4.27E-04	26	4.27E-04	19
182	Antigua & Barbuda	2.25E+03	0.00E+00	-	0.00E+00	-
183	Bermuda	1.94E+03	0.00E+00	-	0.00E+00	-
184	Grenada	1.91E+03	1.05E-03	15	5.23E-04	16
185	Dominica	1.41E+03	0.00E+00	-	0.00E+00	-
186	Sao Tome & Principe	1.35E+03	0.00E+00	-	0.00E+00	-
-	Guadeloupe			-		-
-	Liechtenstein			-		-

-	French Polynesia			-		-	
-	Montenegro			-		-	
-	Aruba			-		-	
-	Macedonia			-		-	
-	Martinique			-		-	
-	Netherlands Antilles			-		-	
-	New Caledonia			-		-	
-	Reunion			-		-	
-	San Marino			-		-	
-	St. Kitts & Nevis			-		-	
-	Anguilla			-		-	
-	Antarctica			-		-	
-	Baker I.			-		-	
-	Bouvet I.			-		-	
-	British Indian Ocean Territory			-		-	
-	British Virgin Is.			-		-	
-	Cayman Is.			-		-	
-	Christmas I.			-		-	
-	Cocos Is.			-		-	
-	Cook Is.			-		-	
-	East Timor			-		-	
-	Falkland Is.			-		-	
-	Faroe Is.			-		-	
-	French Guiana			-		-	
-	French Southern & Antarctic Lands			-		-	
-	Gaza Strip			-		-	
-	Gibraltar			-		-	
-	Glorioso Is.			-		-	
-	Guernsey			-		-	
-	Heard I. & McDonald Is.			-		-	
-	Howland I.			-		-	
-	Isle of Man			-		-	
-	Jan Mayen			-		-	
-	Jarvis I.			-		-	
-	Jersey			-		-	
-	Johnston Atoll			-		-	
-	Juan De Nova I.			-		-	
-	Mayotte			-		-	

-	Micronesia			-		-
-	Midway Is.			-		-
-	Monaco			-		-
-	Montserrat			-		-
-	Nauru			-		-
-	Niue			-		-
-	Norfolk I.			-		-
-	North Korea			-		-
-	Palau			-		-
-	Paracel Is.			-		-
-	Pitcairn Is.			-		-
-	South Georgia & the South Sandwich Is.			-		-
-	Spratly Is.			-		-
-	St. Helena			-		-
-	St. Lucia			-		-
-	St. Pierre & Miquelon			-		-
-	St. Vincent & the Grenadines			-		-
-	Svalbard			-		-
-	Tokelau			-		-
-	Turks & Caicos Is.			-		-
-	Tuvalu			-		-
-	Vatican City			-		-
-	Wake I.			-		-
-	Wallis & Futuna			-		-
-	West Bank			-		-
-	Western Sahara			-		-

Supplementary Figures

Figure S1 - Global OSA related citations and modified h-indices.

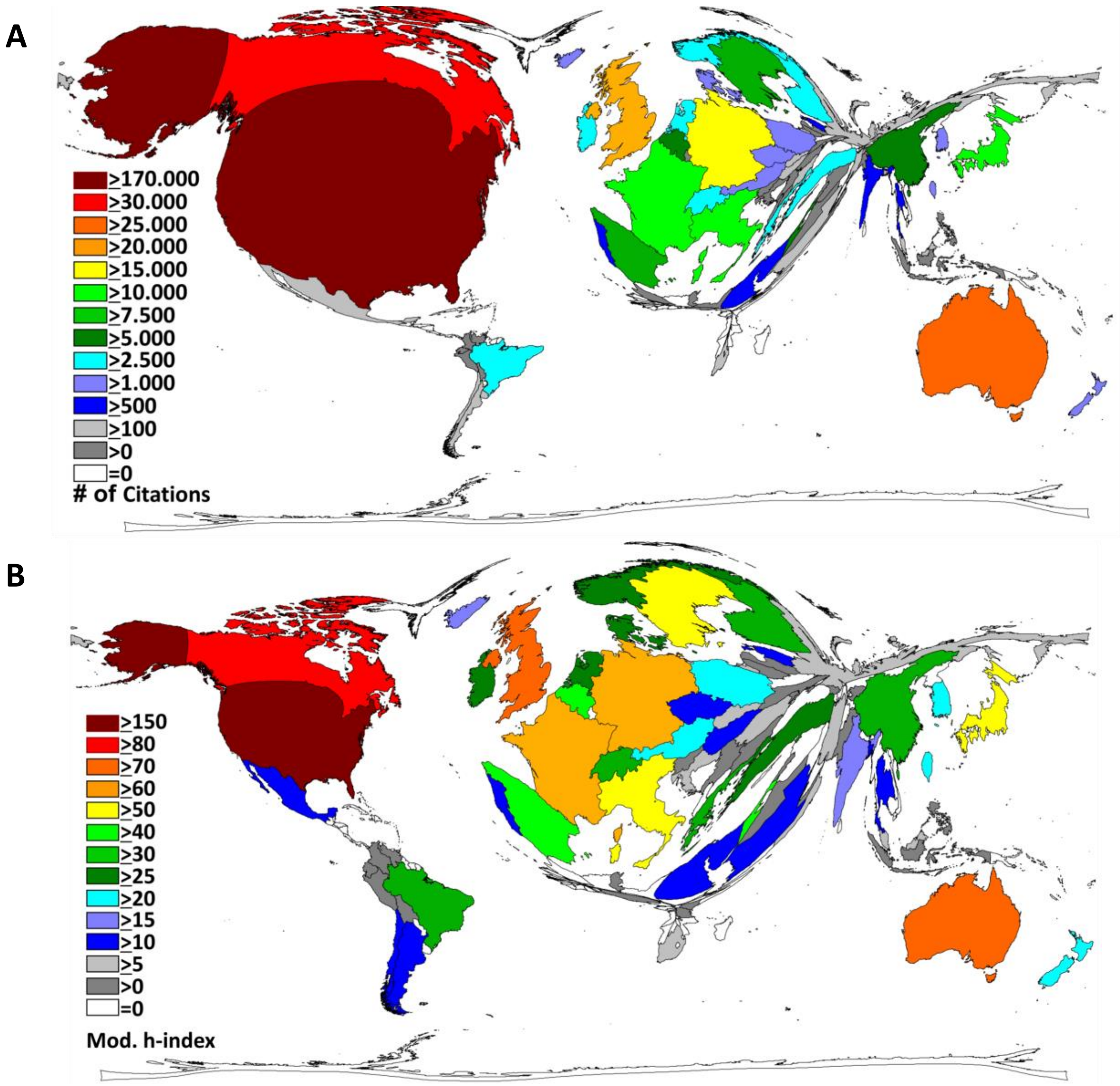

Density Equalizing Map Projections (DEMP) of the country-specific OSA related citations and modified h-indices. A distorted depiction of the world map was generated and the sizes of the countries were determined by the distribution of A) the total number of OSA citations for the period 1900-2013, and B) the modified h-index for the period 1900-2013. Colours are indicating the range of the actual values as illustrated in the respective legends.

Figure S2 - Global OSA publication output in relation to Research & Development expenditure.

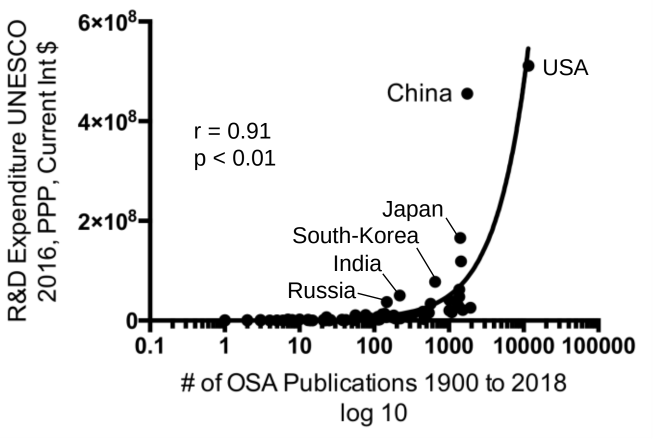

Correlation between the country-specific OSA publication count from 1900 to 2018 and Research & Development (R&D) Expenditure in 2016 (or latest year available) based on Purchasing Power Parity (PPP) in current international Dollar (*1000); source: UNESCO [2].

Figure S3 - Global OSA citation count in relation to economic parameters.

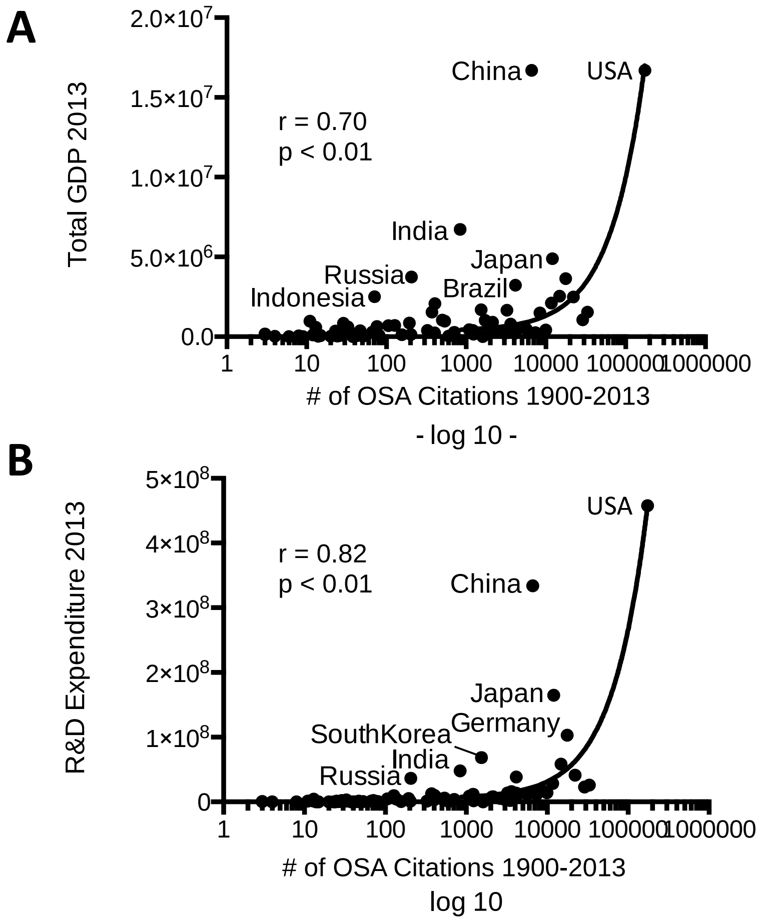

A) Correlation between the country-specific OSA citation count from 1900 to 2013 and the Gross Domestic Product (GDP) in 2013 based on Purchasing Power Parity (PPP) in current international Dollar (Billions); source: International Monetary Fund (IMF) [8].

B) Correlation between the country-specific OSA citation count from 1900 to 2013 and the Research and Development expenditure (R&D) in 2013 based on Purchasing Power Parity (PPP) in current international Dollar (*1000); source: UNESCO [2].

Figure S4 - Global OSA related h-indices in relation to economic parameters.

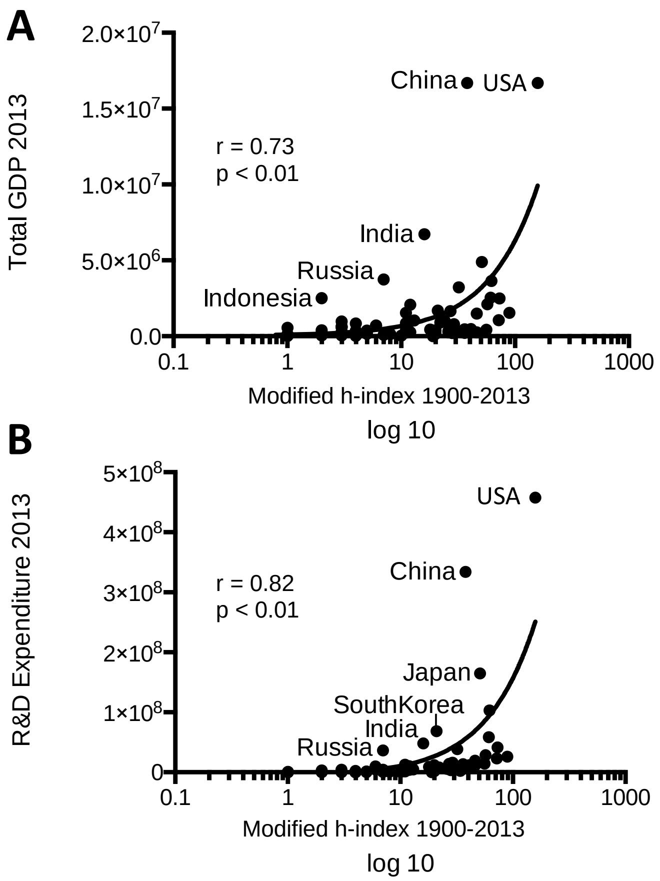

A) Correlation between the countries' OSA-specific modified h-indices (period 1900 to 2013) and Gross Domestic Product (GDP) in 2013 based on Purchasing Power Parity (PPP) in current international Dollar (Billions); source: International Monetary Fund (IMF) [8].

B) Correlation between the countries' OSA-specific modified h-indices (period 1900 to 2013) and Research and Development Expenditure (R&D) in 2013 based on Purchasing Power Parity (PPP) in current international Dollar (*1000); source: UNESCO [2].

Figure S5 - Economic background of international OSA collaborations.

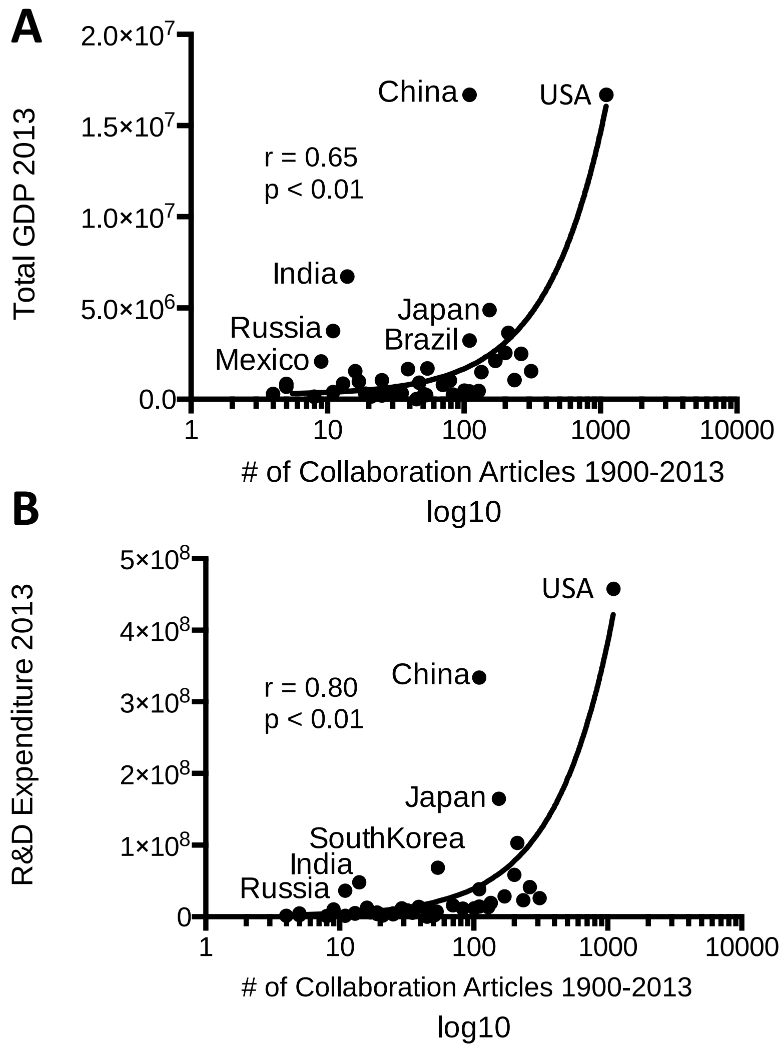

A) Correlation between the country-specific international OSA collaboration articles from 1900 to 2013 and Gross Domestic Product (GDP) in 2013 based on Purchasing Power Parity (PPP) in current international Dollar (Billions); source: International Monetary Fund (IMF) [8].

B) Correlation between the country-specific international OSA collaboration articles from 1900 to 2013 and their Research and Development Expenditure (R&D) in 2013 based on Purchasing Power Parity (PPP) in current international Dollar (*1000); source: UNESCO [2].

Figure S6 - Ratio R1 in comparison to publication activity.

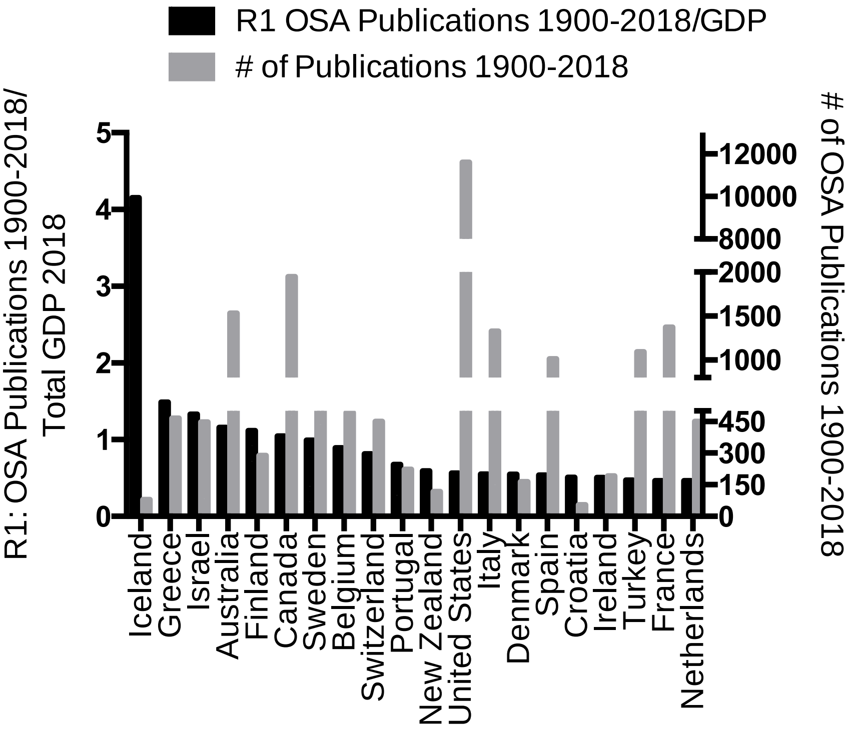

Displayed is the comparison of ratio R1 (OSA related publication output between 1900 and 2018/total Gross Domestic Product in 2018; only countries with ≥ 30 publications) and the OSA related publication output between 1900 and 2018 (top 20 countries in terms of R1). Source of the Gross Domestic Product in 2018 based on Purchasing Power Parity (PPP) in current international Dollar (Billions) was the International Monetary Fund [1].

Figure S7 - Epidemiological influences.

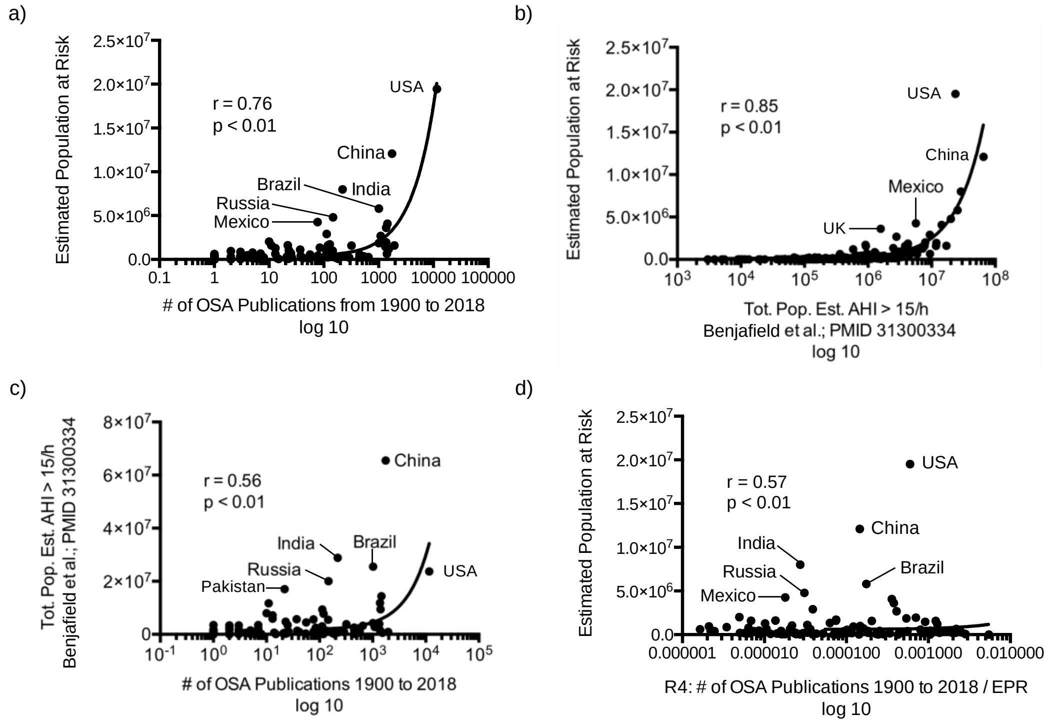

a) Correlation between the country-specific OSA publication output between 1900 and 2018 and the country-specific estimated population at risk (EPR).
b) Correlation between the country-specific estimated population at risk (EPR) and the estimated number of affected individuals with an AHI > 15/h, Benjafield et al. 2019 [9].
c) Correlation between the estimated number of affected individuals with an AHI > 15/h, Benjafield et al. 2019 [9] and the country-specific number of OSA publications from 1900 to 2018.
d) Correlation between the country-specific ratio R4 (OSA publication output 1900-2018/estimated population at risk) and the estimated population at risk in 2013.

References

1 International Monetary Fund (IMF). GDP 2018, Current Prices, based on Purchasing Power Parity; Billions. 2018.http://www.imf.org/external/pubs/ft/weo/2017/01/weodata/weorept.aspx?pr.x=52&pr.y=9&sy=2013&ey=2013&scsm=1&ssd=1&sort=country&ds=%2C&br=1&c=512%2C672%2C914%2C946%2C612%2C137%2C614%2C546%2C311%2C962%2C213%2C674%2C911%2C676%2C193%2C548%2C122%2C556%2C912%2C6 (accessed 3 Jan 2017).

2 UNESCO. Science, technology and innovation: Gross Domestic Expenditure on Research and Development 2016 (or latest year available) in '000, current PPP. 2017.http://data.uis.unesco.org/# (accessed 2 Feb 2019).

3 The World Bank. Male Population 2013 (% of total population). 2017.https://data.worldbank.org/indicator/SP.POP.4044.MA.5Y?end=2016&start=2010 (accessed 2 Feb 2017).

4 NCD Risk Factor Collaboration. Trends in adult body-mass index in 200 countries from 1975 to 2014: a pooled analysis of 1698 population-based measurement studies with 19.2 million participants. *Lancet* 2016;**387**:1377–96. doi:10.1016/S0140-6736(16)30054-X

5 Khajeh-Mehrizi Aminian, O. A. Diagnostic Accuracy of the Multivariable Apnea Prediction (MAP) Index as a Screening Tool for Obstructive Sleep Apnea. *JSS* 2016;**1**.

6 Maislin G, Pack AI, Kribbs NB, *et al.* A survey screen for prediction of apnea. *Sleep* 1995;**18**:158–66.https://www.ncbi.nlm.nih.gov/pubmed/7610311

7 Ng M, Fleming T, Robinson M, *et al.* Global, regional, and national prevalence of overweight and obesity in children and adults during 1980-2013: a systematic analysis for the Global Burden of Disease Study 2013. *Lancet* 2014;**384**:766–81. doi:10.1016/S0140-6736(14)60460-8

8 International Monetary Fund (IMF). World Economic Outlook Database, April 2017, Gross domestic product in 2013 based on purchasing-power-parity (PPP) valuation of country GDP, Current international dollar, Billions. 2017.http://www.imf.org/external/pubs/ft/weo/2017/01/weodata/weorept.aspx?pr.x=52&pr.y=9&sy=2013&ey=2013&scsm=1&ssd=1&sort=country&ds=%2C&b

r=1&c=512%2C672%2C914%2C946%2C612%2C137%2C614%2C546%2C311%2C962%2C213%2C674%2C911%2C676%2C193%2C548%2C122%2C556%2C912%2C6 (accessed 3 Jan 2017).

9 Benjafield A V, Ayas NT, Eastwood PR, *et al.* Estimation of the global prevalence and burden of obstructive sleep apnoea: a literature-based analysis. *Lancet Respir Med* 2019;**7**:687–98. doi:10.1016/S2213-2600(19)30198-5
